# Author Correction: Colonoscopy and fecal immunochemical testing versus usual care in diagnostic colorectal cancer screening: the SCREESCO randomized controlled trial

**DOI:** 10.1038/s41591-026-04702-1

**Published:** 2026-09-10

**Authors:** Marcus Westerberg, Jonas F. Ludvigsson, Chris Metcalfe, Ulf Strömberg, Johannes Blom, Lars Engstrand, Mikael Hellström, Christian Löwbeer, Robert Steele, Lars Holmberg, Anna Forsberg

**Affiliations:** 1 Department of Surgical Sciences, Uppsala University, Uppsala, Sweden; 2 Department of Medical Epidemiology and Biostatistics, Karolinska Institutet, Stockholm, Sweden; 3 Department of Pediatrics, Örebro University Hospital, Örebro, Sweden; 4 Population Health Sciences, Bristol Medical School, University of Bristol, Bristol, UK; 5 School of Public Health and Community Medicine, Institute of Medicine, Sahlgrenska Academy at University of Gothenburg, Gothenburg, Sweden; 6 Department of Surgery, Södersjukhuset, Stockholm, Sweden; 7 Department of Clinical Science and Education, Karolinska Institutet, Stockholm, Sweden; 8 Centre for Translational Microbiome Research, Department of Microbiology, Tumor and Cell Biology, Karolinska Institutet, Solna, Sweden; 9 Department of Radiology, Institute of Clinical Sciences at Sahlgrenska Academy, Gothenburg University, Gothenburg, Sweden; 10 Department of Radiology, Sahlgrenska University Hospital, Gothenburg, Sweden; 11 Department of Laboratory Medicine, Division of Clinical Chemistry, Karolinska Institutet, Stockholm, Sweden; 12 Department of Clinical Chemistry, SYNLAB Sverige, Täby, Sweden; 13 Department of Surgery, Population Health and Genomics, School of Medicine, University of Dundee, Ninewells Hospital, Dundee, UK; 14 Translational Oncology & Urology Research (TOUR), School of Cancer and Pharmaceutical Sciences, King’s College London, London, UK; 15 Division of Clinical Epidemiology, Department of Medicine K2, Solna, Karolinska Institutet, Stockholm, Sweden

**Keywords:** Cancer screening, Colorectal cancer, Cancer screening, Colorectal cancer

Correction to: *Nature Medicine* https://doi.org/10.1038/s41591-026-04225-9, published online 20 February 2026.

In the version of the article initially published, in the first paragraph of the “Diagnostic yield of screening and usual care” section, the sentence “The corresponding cumulative incidence proportions at the end of follow-up of CRC were 0.69% (95% CI: 0.66–0.71%) in the primary colonoscopy arm, 0.72% (95% CI: 0.71–0.73%) in the control arm, 0.61% (95% CI: 0.60–0.63%) in the FIT×2 arm and 0.73% (95% CI: 0.72–0.75%) in the FIT×2 control arm” should have read “The corresponding cumulative incidence proportions at the end of follow-up of CRC were 0.69% (95% CI: 0.56-0.81%) in the primary colonoscopy arm, 0.72% (95% CI: 0.66-0.78%) in the control arm, 0.61% (95% CI: 0.54-0.68%) in the FIT×2 arm and 0.73% (95% CI: 0.67-0.79%) in the FIT×2 control arm”. This correction has been made in the HTML and PDF versions of the article.

