# Supplementary material for: Colonoscopy and fecal immunochemical testing versus usual care in diagnostic colorectal cancer screening: the SCREESCO randomized controlled trial
Source: Nat Med. 2026 Feb 20;32(4):1278–85. doi: 10.1038/s41591-026-04225-9 (PMC13099434; doi:10.1038/s41591-026-04225-9)
Supplement: Supplementary file 1 — Supplementary Tables 1–5 and Note 1 (statistical analysis plans and protocols). [file 41591_2026_4225_MOESM1_ESM.pdf]

nature medicine

Supplementary information https://doi.org/10.1038/s41591-026-04225-9

Colonoscopy and fecal immunochemical testing versus usual care in diagnostic colorectal cancer screening: the SCREESCO randomized controlled trial

In the format provided by the authors and unedited

Table of contents

Supplementary Table 1. 2
Supplementary Table 2. 3
Supplementary Table 3 6
Supplementary Table 3 (continued) 7
Supplementary Table 4. 4
Supplementary Table 4 (continued) 5
Supplementary Table 5. 8
Supplementary Note 1 (Study protocols and statistical analysis plans) 9

Supplementary Table 1. Baseline characteristics (at date of randomization) and outcomes during the intervention phase separately in participants and non-participants of the intervention arms.

	Primary colonoscopy arm				FITx2 arm			
	Participant		Non-participant		Participant		Non-participant	
	N	(%)	N	(%)	N	(%)	N	(%)
**N individuals**	10679	(100.0)	20434	(100.0)	33383	(100.0)	26884	(100.0)
**Sex**								
Men	5507	(51.6)	10043	(49.1)	15519	(46.5)	14609	(54.3)
Women	5172	(48.4)	10391	(50.9)	17864	(53.5)	12275	(45.7)
**Year of randomization**								
2014	2384	(22.3)	4313	(21.1)	11523	(34.5)	8568	(31.9)
2015	2281	(21.4)	4415	(21.6)	11026	(33.0)	9062	(33.7)
2016	2297	(21.5)	4396	(21.5)	10834	(32.5)	9254	(34.4)
2017	1922	(18.0)	3589	(17.6)	0	(0.00)	0	(0.00)
2018	1795	(16.8)	3721	(18.2)	0	(0.00)	0	(0.00)
**Health care region of residence**								
North	1156	(10.8)	1857	(9.1)	3313	(9.9)	2470	(9.2)
Central	2994	(28.0)	5812	(28.4)	9783	(29.3)	7661	(28.5)
Southeast	1480	(13.9)	2940	(14.4)	4619	(13.8)	3865	(14.4)
South	2214	(20.7)	4560	(22.3)	6887	(20.6)	6106	(22.7)
West	2835	(26.5)	5265	(25.8)	8781	(26.3)	6782	(25.2)
**Educational level**								
Low	1555	(14.6)	4149	(20.3)	5331	(16.0)	6238	(23.2)
Intermediate	5002	(46.8)	10225	(50.0)	15770	(47.2)	13484	(50.2)
High	4108	(38.5)	5985	(29.3)	12243	(36.7)	7010	(26.1)
Missing	14	(0.13)	75	(0.37)	39	(0.12)	152	(0.57)
**Country of birth**								
Sweden	9611	(90.0)	17765	(86.9)	29415	(88.1)	23739	(88.3)
Other	1068	(10.0)	2669	(13.1)	3968	(11.9)	3145	(11.7)
**Charlson comorbidity index**								
0	8699	(81.5)	16139	(79.0)	26911	(80.6)	21218	(78.9)
1	947	(8.9)	2004	(9.8)	3188	(9.5)	2661	(9.9)
2	791	(7.4)	1525	(7.5)	2409	(7.2)	1971	(7.3)
≥3	242	(2.3)	766	(3.7)	875	(2.6)	1034	(3.8)
**Drug comorbidity index****								
<0	1467	(13.7)	2124	(10.4)	4565	(13.7)	2715	(10.1)
0 to 0.22	3905	(36.6)	7864	(38.5)	12241	(36.7)	10614	(39.5)
0.23 to 0.91	2862	(26.8)	5005	(24.5)	8908	(26.7)	6461	(24.0)
≥0.92	2445	(22.9)	5441	(26.6)	7669	(23.0)	7094	(26.4)
**Any prior cardiovascular or gastrointestinal event**								
No	9939	(93.1)	18767	(91.8)	31053	(93.0)	24671	(91.8)
Yes	740	(6.9)	1667	(8.2)	2330	(7.0)	2213	(8.2)
Cardiovascular event	574	(5.4)	1361	(6.7)	1815	(5.4)	1866	(6.9)
Gastrointestinal event	195	(1.8)	370	(1.8)	613	(1.8)	439	(1.6)
**Events occurring during the intervention phase**								
CRC (any)	57	(0.53)	103	(0.50)	170	(0.51)	160	(0.60)
CRC Stage I-II	38	(0.36)	49	(0.24)	111	(0.33)	70	(0.26)
CRC Stage III-IV	17	(0.16)	53	(0.26)	53	(0.16)	86	(0.32)
Cardiovascular event	636	(6.0)	1475	(7.2)	2315	(6.9)	2586	(9.6)
Gastrointestinal event	129	(1.2)	313	(1.5)	655	(2.0)	538	(2.0)
Death from any cause	105	(0.98)	720	(3.5)	503	(1.5)	1486	(5.5)

Individuals in the primary colonoscopy arm were considered to be participants if they underwent a screening colonoscopy while individuals in the FITx2 arm were considered participants if they returned a FIT in any of the two rounds.

Supplementary Table 2. Stage according to SCREESCO vs The Swedish Colorectal Cancer Quality Register combined with The Swedish Cancer Register in 177 individuals with colorectal cancer detected at screening colonoscopy within SCREESCO.

**Arm**	**Stage in SCREESCO**	**Stage in the registries**		
		I-II	III-IV	Missing
Both	I-II	**122 (92%)**	1 (0.8%)	9 (6.8%)
	III-IV	2 (4.4%)	**43 (96%)**	0 (0.0%)
Primary colonoscopy	I-II	**35 (90%)**	1 (2.6%)	3 (7.7%)
	III-IV	0 (0%)	**13 (100%)**	0 (0.0%)
FITx2	I-II	**87 (94%)**	0 (0.0%)	6 (6.5%)
	III-IV	2 (6.3%)	**30 (94%)**	0 (0.0%)

Supplementary Table 3. Incidence rate (per 100,000 person-years) of individuals diagnosed with colorectal cancer during the diagnostic phase, by sex.

Stage	Sex	Arm	Screen detected	Events	%	Person years	Rate	95% CI	IRR*	95% CI	Effect modification (by sex) IRR* men / IRR* women	95% CI
Any	Men	Primary colonoscopy	Any	86	0.55	73,819	116.6	(94.9-143.9)	1.08	(0.86-1.36)	1.00	(0.71-1.40)
			Yes	31	0.20							
			No	55	0.35							
		Control	No	478	0.51	443,352	107.8	(98.6-117.9)	1.00			
	Women	Primary colonoscopy	Any	74	0.48	74,518	99.3	(79.1-124.7)	1.08	(0.84-1.38)		
			Yes	20	0.13							
			No	54	0.35							
		Control	No	411	0.44	446,422	92.1	(83.6-101.4)	1.00			
	Men	Fitx2	Any	197	0.65	170,891	115.3	(100.3-132.6)	1.03	(0.87-1.22)	1.29	(0.99-1.67)
			Yes	64	0.21							
			No	133	0.44							
		Control	No	383	0.64	342,289	111.9	(101.2-123.7)	1.00			
	Women	Fitx2	Any	133	0.44	172,812	77.0	(64.9-91.2)	0.80	(0.66-0.98)		
			Yes	60	0.20							
			No	73	0.24							
		FITx2 Control	No	331	0.55	344,759	96.0	(86.2-106.9)				
I-II	Men	Primary colonoscopy	Any	41	0.26	73,819	55.5	(40.9-75.4)	1.18	(0.84-1.65)	0.72	(0.45-1.15)
			Yes	20	0.13							
			No	21	0.14							
		Control	No	209	0.22	443,352	47.1	(41.2-54.0)	1.00			
	Women	Primary colonoscopy	Any	46	0.30	74,518	61.7	(46.2-82.4)	1.63	(1.18-2.26)		
			Yes	15	0.10							
			No	31	0.20							
		Control	No	169	0.18	446,422	37.9	(32.6-44.0)	1.00			
	Men	Fitx2	Any	103	0.34	170,891	61.3	(49.7-73.1)	1.23	(0.96-1.57)	1.08	(0.75-1.57)
			Yes	48	0.16							
			No	55	0.18							
		Control	No	168	0.28	342,289	49.1	(42.2-57.1)	1.00			
	Women	Fitx2	Any	78	0.26	172,812	45.1	(36.2-56.4)	1.14	(0.86-1.50)		
			Yes	41	0.14							
			No	37	0.12							
		FITx2 Control	No	137	0.23	344,759	39.7	(33.6-47.0)	1.00			

Supplementary Table 3 (continued). Incidence rate (per 100,000 person-years) of individuals diagnosed with colorectal cancer during the diagnostic phase, by sex.

Stage	Sex	Arm	Screen detected	Events	%	Person years	Rate	95% CI	IRR*	95% CI	Effect modification (by sex) IRR* men / IRR* women	95% CI
III-IV	Men	Primary colonoscopy	Any	43	0.28	73,819	58.3	(43.2-78.5)	1.01	(0.73-1.39)	1.45	(0.87-2.42)
			Yes	10	0.06							
			No	33	0.21							
		Control	No	256	0.27	443,352	57.7	(51.1-65.3)				
	Women	Primary colonoscopy	Any	27	0.17	74,518	36.2	(24.8-52.8)	0.70	(0.47-1.04)		
			Yes	4	0.03							
			No	23	0.15							
		Control	No	232	0.25	446,422	52.0	(45.7-59.1)				
	Men	Fitx2	Any	88	0.29	170,891	51.5	(41.8-63.5)	0.85	(0.66-1.09)	1.56	(1.05-2.32)
			Yes	15	0.05							
			No	73	0.24							
		Control	No	207	0.34	342,289	60.5	(52.8-69.3)				
	Women	Fitx2	Any	51	0.17	172,812	29.5	(22.4-38.8)	0.55	(0.40-0.75)		
			Yes	15	0.05							
			No	36	0.12							
		FITx2 Control	No	186	0.31	344,759	54.0	(46.7-62.3)				

* IRR=Incidence rate ratio (rate arm / rate control).

Supplementary Table 4. Incidence rate (per 100,000 person-years) of individuals experiencing a cardiovascular or gastrointestinal event during the diagnostic phase, by type of event.

Arm	Type of event	Events	%	Person years	Rate	95% CI	IRR*	95% CI
Primary colonoscopy	**Any cardiovascular event**	236	0.76	148,284	159.2	(140.1-180.8)	1.05	(0.92-1.21)
Control		1343	0.72	889,187	151.0	(143.2-159.3)	1.00	
Fitx2		520	0.86	343,638	151.3	(138.9-164.9)	0.97	(0.87-1.08)
FITx2 Control		1071	0.89	686,562	156.0	(146.9-165.6)	1.00	
Primary colonoscopy	Ischemic Heart Disease	1449	4.7	144,531	1002.6	(952.2-1055.5)	1.00	(0.95-1.06)
Control		8671	4.6	866,469	1000.7	(979.9-1022.0)	1.00	
Fitx2		3324	5.5	333,910	995.5	(962.2-1029.9)	1.00	(0.96-1.05)
FITx2 Control		6614	5.5	667,354	991.1	(967.5-1015.3)	1.00	
Primary colonoscopy	Pulmonary embolism	254	0.82	148,186	171.4	(151.6-193.8)	1.02	(0.89-1.16)
Control		1495	0.80	888,568	168.2	(159.9-177.0)	1.00	
Fitx2		575	0.95	343,331	167.5	(154.3-181.7)	1.00	(0.90-1.11)
FITx2 Control		1149	0.95	686,106	167.5	(158.1-177.4)	1.00	
Primary colonoscopy	Acute endocarditis	23	0.07	148,704	15.5	(10.3-23.3)	1.09	(0.70-1.69)
Control		127	0.07	891,371	14.2	(12.0-17.0)	1.00	
Fitx2		48	0.08	344,580	13.9	(10.5-18.5)	0.89	(0.63-1.25)
FITx2 Control		108	0.09	688,456	15.7	(13.0-18.9)	1.00	
Primary colonoscopy	Cardiac arrest	86	0.28	148,696	57.8	(46.8-71.4)	0.82	(0.66-1.03)
Control		625	0.33	891,225	70.1	(64.8-75.8)	1.00	
Fitx2		233	0.39	344,481	67.6	(59.5-76.9)	0.97	(0.83-1.13)
FITx2 Control		481	0.40	688,346	69.9	(63.9-76.4)	1.00	
Primary colonoscopy	Cerebral infarction	351	1.1	147,862	237.4	(213.8-263.6)	0.98	(0.87-1.09)
Control		2153	1.2	886,665	242.8	(232.8-253.3)	1.00	
Fitx2		814	1.4	342,526	237.6	(221.9-254.5)	0.95	(0.88-1.04)
FITx2 Control		1704	1.4	684,378	249.0	(237.4-261.1)	1.00	
Primary colonoscopy	Peripheral artery embolism	32	0.10	148,670	21.5	(15.2-30.4)	0.78	(0.54-1.12)
Control		247	0.13	891,133	27.7	(24.5-31.4)	1.00	
Fitx2		85	0.14	344,460	24.7	(20.0-30.5)	0.88	(0.69-1.14)
FITx2 Control		192	0.16	688,251	27.9	(24.2-32.1)	1.00	
Primary colonoscopy	Venous thromboembolism	72	0.23	148,608	48.4	(38.5-61.0)	1.08	(0.84-1.39)
Control		400	0.21	890,910	44.9	(40.7-49.5)	1.00	
Fitx2		207	0.34	344,199	60.1	(52.5-68.9)	1.39	(1.16-1.66)
FITx2 Control		298	0.25	688,100	43.3	(38.7-48.5)	1.00	

* IRR=Incidence rate ratio (rate arm / rate control).

Supplementary Table 4 (continued). Incidence rate (per 100,000 person-years) of individuals experiencing a gastrointestinal or cardiovascular event during the diagnostic phase, by type of event.

Arm	Type of event	Events	%	Person years	Rate	95% CI	IRR*	95% CI
Primary colonoscopy	**Any gastrointestinal event**	442	1.4	147,671	299.3	(272.7-328.6)	0.99	(0.89-1.09)
Control		2689	1.4	885,246	303.8	(292.5-315.5)	1.00	
Fitx2		1193	2.0	341,297	349.5	(330.3-370.0)	1.12	(1.04-1.20)
FITx2 Control		2134	1.8	683,074	312.4	(299.4-326.0)	1.00	
Primary colonoscopy	Splenic injury	10	0.03	148,726	6.7	(3.6-12.5)	1.58	(0.79-3.17)
Control		38	0.02	891,578	4.3	(3.1-5.9)	1.00	
Fitx2		14	0.02	344,641	4.1	(2.4-6.9)	1.08	(0.56-2.06)
FITx2 Control		26	0.02	688,644	3.8	(2.6-5.5)	1.00	
Primary colonoscopy	Colonic injury	0	0.00	148,748	0.0			
Control		19	0.01	891,648	2.1	(1.4-3.3)		
Fitx2		9	0.01	344,657	2.6	(1.4-5.0)	1.20	(0.52-2.74)
FITx2 Control		15	0.01	688,698	2.2	(1.3-3.6)	1.00	
Primary colonoscopy	Rectal injury	2	0.01	148,742	1.3			
Control		8	0.00	891,663	0.90	(0.45-1.8)		
Fitx2		3	0.00	344,665	0.87			
FITx2 Control		7	0.01	688,712	1.0	(0.48-2.1)		
Primary colonoscopy	Bleeding iatrogenic	169	0.54	148,310	114.0	(98.0-132.5)	0.97	(0.82-1.14)
Control		1047	0.56	889,070	117.8	(110.8-125.1)	1.00	
Fitx2		487	0.81	343,278	141.9	(129.8-155.0)	1.18	(1.06-1.32)
FITx2 Control		825	0.68	686,449	120.2	(112.3-128.7)	1.00	
Primary colonoscopy	Perforation	43	0.14	148,651	28.9	(21.5-39.0)	0.88	(0.64-1.22)
Control		292	0.16	890,982	32.8	(29.2-36.8)	1.00	
Fitx2		113	0.19	344,400	32.8	(27.3-39.5)	0.99	(0.79-1.24)
FITx2 Control		229	0.19	688,117	33.3	(29.2-37.9)	1.00	
Primary colonoscopy	Unspecified gastrointestinal bleeding	237	0.76	148,202	159.9	(140.8-181.6)	1.03	(0.90-1.18)
Control		1380	0.74	888,527	155.3	(147.3-163.7)	1.00	
Fitx2		632	1.0	342,861	184.3	(170.5-199.3)	1.14	(1.04-1.26)
FITx2 Control		1106	0.92	685,937	161.2	(152.0-171.0)	1.00	

* IRR=Incidence rate ratio (rate arm / rate control).

Supplementary Table 5. Incidence rate (per 100,000 person-years) of individuals that died or experienced a cardiovascular or gastrointestinal event during the diagnostic phase, by type of event and sex.

Type of event	Sex	Arm	Events	%	Person years	Rate	95% CI	IRR*	95% CI	Effect modification (by sex) IRR* men / IRR* women	95% CI
Cardiovascular event	Men	Primary colonoscopy	1466	9.4	69,950	2095.8	(1991.2-2205.9)	1.00	(0.95-1.06)	1.01	(0.92-1.12)
		Control	8775	9.4	420,545	2086.6	(2043.4-2130.7)	1.00			
	Women	Primary colonoscopy	645	4.1	73,087	882.5	(817.0-953.3)	0.99	(0.91-1.08)		
		Control	3887	4.2	437,442	888.6	(861.1-917.0)	1.00			
	Men	Fitx2	3380	11.2	161,309	2095.4	(2025.9-2167.2)	1.01	(0.97-1.05)	0.99	(0.92-1.07)
		FITx2 Control	6700	11.1	322,972	2074.5	(2025.4-2124.8)	1.00			
	Women	Fitx2	1521	5.0	168,853	900.8	(856.6-947.2)	1.02	(0.96-1.08)		
		FITx2 Control	2988	5.0	337,151	886.2	(855.0-918.6)	1.00			
Gastrointestinal event	Men	Primary colonoscopy	239	1.5	73,476	325.3	(286.6-369.2)	0.95	(0.83-1.09)	0.92	(0.75-1.12)
		Control	1513	1.6	440,796	343.2	(326.4-361.0)	1.00			
	Women	Primary colonoscopy	203	1.3	74,195	273.6	(238.4-314.0)	1.03	(0.89-1.20)		
		Control	1176	1.3	444,451	264.6	(249.9-280.2)	1.00			
	Men	Fitx2	664	2.2	169,676	391.3	(362.7-422.3)	1.10	(1.00-1.21)	0.97	(0.84-1.11)
		FITx2 Control	1207	2.0	340,048	354.9	(335.5-375.5)	1.00			
	Women	Fitx2	529	1.8	171,621	308.2	(283.1-335.7)	1.14	(1.03-1.27)		
		FITx2 Control	927	1.5	343,026	270.2	(253.4-288.2)	1.00			
Death	Men	Primary colonoscopy	505	3.2	74,042	682.0	(625.1-744.2)	0.99	(0.90-1.08)	1.07	(0.92-1.25)
		Control	3075	3.3	444,370	692.0	(668.0-716.9)	1.00			
	Women	Primary colonoscopy	320	2.1	74,706	428.3	(383.9-477.9)	0.92	(0.82-1.03)		
		Control	2088	2.2	447,312	466.8	(447.2-487.2)	1.00			
	Men	Fitx2	1225	4.1	171,471	714.4	(675.5-755.6)	0.99	(0.93-1.06)	1.09	(0.98-1.22)
		FITx2 Control	2470	4.1	343,177	719.7	(691.9-748.7)	1.00			
	Women	Fitx2	764	2.5	173,200	441.1	(410.9-473.5)	0.91	(0.84-0.99)		
		FITx2 Control	1675	2.8	345,552	484.7	(462.1-508.5)	1.00			

* IRR=Incidence rate ratio (rate arm / rate control

SCREESCO – Screening of Swedish Colons

Study Protocol
131220

Summary
Colorectal cancer (CRC) is a major cause of death in Sweden. There are approximately 6000 new cases each year in Sweden and the disease specific mortality is more than 40%. The risk is about 1% to develop CRC between 60-70 years of age, making 60-year-olds a suitable target population for colorectal cancer screening.

The Swedish Ministry of Health and Social affairs has proposed a national study on the efficiency of colorectal cancer screening in the Swedish population regarding mortality, but also what screening method to be used. Thirteen participating counties of Sweden now fund the study to be launched in 2014.

Individuals 60 years of age will be randomized from the population register and invited to screening by mail. Twenty thousand individuals will be invited to primary colonoscopy and 60 000 individuals will be invited to high sensitive FIT (OC Sensor®) with approximately 10% positivity rate and, if positive, to a subsequent follow-up colonoscopy. When test negative a second round of FIT will be asked for in two years. In total 120 000 randomized individuals will not be invited to screening serve as controls and will be followed in the Swedish Cancer Register. The inclusion period is set to three years (five years including the second round of FIT) generating approximately 5 000 colonoscopies yearly at a compliance rate of 50% in the colonoscopy arm and 60% in the FIT arm.

Follow-up time is set to 15 years with the primary endpoint disease specific mortality and colorectal cancer incidence. Secondary outcomes, by others, to be studied are in short quality assurance variables of colonoscopy, participants and non-participants experiences of the invitation and the screening procedure, health economy measures of the CRC-screening study and when implemented in clinical care.

Table of contents

1. Background 4
1.1 Colorectal cancer and screening 4
1.2 Evaluation of a screening program 4
1.2.1 Effectiveness 4

1.2.2 The screening test *5*
1.2.3 Compliance *6*
1.2.4 Cost-effectiveness *6*
1.3 Challenges in colorectal cancer screening **7**
1.3.1 Colonoscopy quality assurance *7*
1.3.2 Lack of pathologists *7*
1.3.3 Compliance and emotional effects *7*
1.4 Colorectal cancer screening in Sweden **7**
1.5 A call for a population-based Swedish CRC-screening study **8**

2. Study aims **8**
2.1 Primary endpoints **8**
2.2 Secondary endpoints **8**

3. Material and Methods **9**
3.1 Study population **9**
3.1.1 Inclusion criteria *9*
3.1.2 Exclusion criteria *9*
3.1.3 Randomization *9*
3.2 Invitation procedures **9**
3.2.1 FIT *9*
3.2.2 Colonoscopy *10*
3.2.3 Controls *10*
3.2.4 Undelivered invitations *10*
3.3 Interventions **10**
3.3.1 FIT-arm and the positivity rate *10*
3.3.1.1 Two rounds of FIT 10
3.3.1.2 Information of FIT-result 11
3.3.1.3 Reminder and default returned FIT 11
3.3.1.4 FIT returned after 6 months 11
3.3.1.5 Follow-up colonoscopy appointment of individuals with positive FIT 11
3.3.1.6 Bowel preparation for follow-up colonoscopy 11
3.3.1.7 Medical history taken at telephone confirmation of appointment 11
3.3.1.8 Reminder of follow-up colonoscopy 11
3.3.1.9 Follow-up after positive FIT follow-up colonoscopy 11
3.1.1.9.10 Positive FIT and asking for a new FIT replacing colonoscopy 12
3.3.2 Colonoscopy-arm *12*
3.3.2.1 Primary colonoscopy appointment 12
3.3.2.2 Weight and height measurement and nurse questionnaire 12
3.3.2.3 Blood sample at colonoscopy 12
3.3.2.4 Positive finding at colonoscopy 12
3.3.2.5 Individuals with positive FIT but negative colonoscopy 13
3.3.2.6 Questionnaire after the colonoscopy 13
3.4 Informed consent **13**
3.5 Quality assurance of colonoscopy **13**
3.6 Pathology **14**
3.7 Secondary studies **14**
3.7.1 Complier and non-complier experiences *14*

3.7.2 Health-economy 14
3.7.3 Quality assurance 14
3.7.4 Surveillance of adenomas 14
3.7.5 DNA in blood 14
3.7.6 Bacteria in feces 14
3.7.7 Non-steroid anti-inflammatory drugs and adenoma development 14
3.8 Follow-up 14

4. Ethical considerations 15

5. Statistical analyses 15

6. Data management 16

7. Head secretariat 16

8. Participating centers 16

9. Main study publications 16

10. Scientific committee 17

11. Figures 18
11.1 Figure 1. Flow chart of invitation procedure of individuals randomized to intervention FIT 18
11.2 Figure 2. Flow chart of invitation procedure of individuals randomized to intervention primary colonoscopy 19

12. References 20

1. Background

1.1 Colorectal cancer and screening

The primary purpose of cancer screening is to reduce mortality from the disease in the population by finding the cancer at an early and treatable stage. The cancer has to be an important health problem to be a suitable target for screening. With approximately 6 000 new cases each year in Sweden (1),

colorectal cancer (CRC) is the third most common cancer in Sweden (after prostate- and breast cancer) and, hence, a relatively common disease, but it is also a major cause of death. There is a 1% risk to develop CRC between the ages 60-75 years, and the five-year survival rate is close to 60%. The prognosis is related to if the cancer is detected at an early or late stage of the disease. When detected at an early stage, there is a 90% five-year survival, as compared to only a 10% five-year survival if the cancer at detected at a late stage.

CRC is usually detected clinically by patient symptoms, either an alteration in bowel habits due to obstruction of the lumen, visual blood in the stool or symptoms due to anemia caused by a bleeding from the tumor. Both larger precursor stages – the adenomatous polyp (2, 3) – and cancers bleed and could be detected by sensitive methods to analyze blood in the stool. All patients diagnosed with colorectal cancer need treatment, but there is a significant difference in suffers and costs depending on the stage the disease at diagnosis. With screening early stages of the disease will be found before they are clinically detected.

Removal of adenomatous polyps (adenomas) has a protective effect against colorectal cancer development (4-6) and, consequentially, a colorectal cancer screening program also might have the potential of decreasing the future incidence of the disease.

1.2 Evaluation of a screening program

1.2.1 Effectiveness

The effectiveness of a screening program is the ability of the program to reduce the disease specific mortality. Survival is not a valid measure of effectiveness because of the possibilities of bias; selection bias (when screened subjects and non-screened controls represent different populations), lead time bias (earlier diagnosis in screen-detected cancers adds time to the total survival time) and length biased sampling (screen-detected tumors often grow slowly and might be less malignant).

The most valid measure of effectiveness in screening is a lower mortality in the screened group, as compared to the non-screened group evaluated in a randomized controlled trial (RCT):

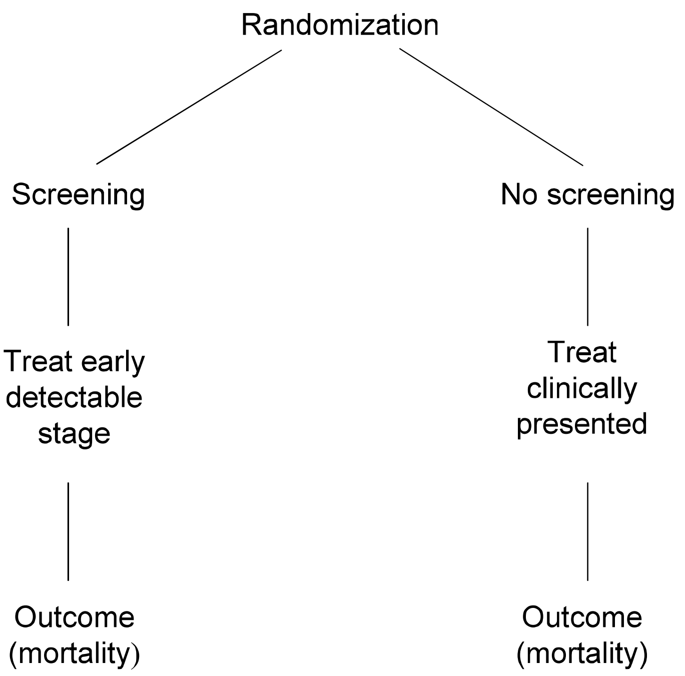

1.2.2 The screening test

A very large majority of individuals in the general population using the screening test offered will not have colorectal cancer. Therefore the test must be free from unwanted side effects, inexpensive, but also simple to take and easy to interpret (7). Furthermore, a high sensitivity (to limit the number of missed cancers) and specificity (to limit the number of incorrect diagnosed cancers), is a prerequisite for a screening test to be used in a program of the average risk population.

There are a number of screening tests and methods to examine the colorectum in order to find CRC and/or adenomatous polyps:

<u>Indirect tests:</u> The most commonly used screening test has been the guaiac based fecal occult blood test (FOBT) (Hemoccult®). Four larger RCTs have demonstrated a 16% decreased in CRC mortality with the test in screening (8-11). The degree of mortality reduction with Hemoccult® depends on the compliance with the test and the dietary restrictions, the sensitivity of the test, the screening frequency (annual or biennial), the number of screening rounds the subjects participate in and, also, the compliance with the diagnostic follow-up colonoscopy after a positive test. More advanced fecal immunological tests (FITs) with higher sensitivity, but only a marginally increase in the false positive rate (specificity), are now available. FIT demonstrates presence of human blood only, as opposed to the guaiac test that also can be positive due to animal hemoglobin. Moreover, no dietary restrictions are needed with FIT.

<u>Direct tests:</u> The main advantages with endoscopy (*e.g.* colonoscopy and sigmoidoscopy) are the direct visualization of the colorectum and the possibility of obtaining tissue samples from suspected cancer lesions for histopathology and/or removing adenomatous polyps during the procedure. Suggestively, the bowel can be examined with sigmoidoscopy and a subsequent colonoscopy in

case of pathological finding within the reach of the sigmoidoscope (approximately 60 cm) or with a complete colonoscopy as primary test (when 1/3 of tumors will appear in the right part of the colon). Recently, three randomized controlled European trials with sigmoidoscopy as the primary screening test have demonstrated both a reduced disease specific mortality of around 30%, but also a reduced incidence of CRC of as much as 40% (12-14).

Up to now, there are no larger randomized controlled studies of the average risk population published demonstrating a disease specific mortality reduction with colonoscopy as the primary screening test.

1.2.3 Compliance

The proportion of individuals offered a screening test and actually take the test is referred to as compliance (7). The compliance to a screening program is a major determinant of the programs effectiveness and there has to be a rigid organization with a call- recall system and quality assurance in a screening program to be effective (15).

Instead of number needed to treat (NNT) used to estimate the efficacy in interventional RCTs evaluating medication, the number needed to screen (NNS) is used in the evaluation of RCTs of screening. The NNS is the number of individuals who need to be invited (offered) screening to prevent one death (intention-to-screen). The results then reflect the efficacy of the screening program to reduce mortality among those *invited* to screening. With NNS there is often an underestimation of the efficacy of the screening test in those people who actually participate – are being screened. The NNS for most screening programs are usually much higher than the number of people who have to participate to prevent one death (16). It is only the participants that can contribute to the mortality reduction achieved by the screening program and with low compliance the number of deaths prevented will be few, and consequently the NNS will be very large (16). Therefore, a high participation rate in a screening program is very important to be able to evaluate its effectiveness on mortality.

1.2.4 Cost-effectiveness

There are a large variety of variables involved when measuring the cost-effectiveness of a colorectal cancer screening program and focus cannot only be on the eventual incidence and mortality reduction of the disease. Firstly, one have to make assumptions about the duration of the early, asymptomatic and curable stage of the disease. Secondly, one has to estimate the effectiveness and negative effects of the screening procedure, such as morbidity due to complications and costs. A low compliance in a screening program will both effect the incidence and mortality reduction achieved and the cost-effectiveness of the program.

1.3 Challenges in colorectal cancer screening

1.3.1 Colonoscopy quality assurance

There are some outstanding challenges in implementing colorectal cancer screening for the average risk population. One very important issue is the quality assurance of the endoscopic examinations and the follow-up of eventual findings. If the quality of the examinations is not excellent, but with frequent practical mistakes and neglect to find adenomas and or cancers, the positive effect of the screening procedure for that particular individual and of the program as a whole is both diminished and unethical (17-21).

Colonoscopy resources and quality will be a key issue in the present study. Currently in Sweden, about 65 000 procedures are carried out outside the Stockholm area yearly, while approximately 30 000 are carried out in Stockholm. A crude estimate is that a future national screening program will need a substantial number of colonoscopies, and, thus, much work is needed to both increase the number of colonoscopies performed nationally and also increase and secure the quality of the investigations.

Adenomatous polyps should be removed in order to prevent the occurrence of CRC. The removal of adenomas is a sometimes difficult and dangerous procedure, since it may cause bleeding and perforation of the bowel wall. It is very important that the adenoma is completely removed and correctly diagnosed. Thus, both a skillful endoscopists and high-quality histopathology are extremely important.

1.3.2 Lack of pathologists

Another problem when implementing a screening program is the created increased burden for pathologists. There is a need for standardization of the use of the diagnostic criteria and the possibility to have a group of pathologists to evaluate the sections. In order to do so, it would be of great advantage if the histological sections could be stained and digitally processed and evaluated electronically.

1.3.3 Compliance and emotional effects

Another important factor, in need of further studies, is the actual adherence rate or compliance discussed above and the emotional effects of the inclusion of healthy persons from the general population in colorectal cancer screening (22-25).

1.4 Colorectal cancer screening in Sweden

The National Board of Health and Welfare has not been recommending CRC screening due to lack of experiences of screening in routine health care and refer CRC screening to the Research and Development list waiting for studies outside Sweden to generate results. Based on the results from the four RCTs with a net CRC-mortality reduction of 16% (8-11) the Council of the European Union in 2003 recommended CRC-screening with guaiac-based fecal occult blood test (FOBT) in the average risk population aged 55-74 years old. The recommendations have recently been updated in the European Guidelines of Quality Assurance in CRC-screening (26) and as a result, national CRC-screening

programs with guaiac-based FOBT have started around Europe, *e.g.* United Kingdom, Finland, France, Italy and shortly in Denmark and Norway. In Sweden, only the counties of Stockholm/Gotland have an organized colorectal cancer screening program of the 60-69 years old general population using guaiac-based FOBT as the primary screening test.

1.5 A call for a population-based Swedish CRC-screening study

The Swedish minister of Health and Social affairs, Göran Hägglund, funded a task force in 2011 in order to design a study of the effectiveness of colorectal cancer screening of the average risk population of Sweden. The Swedish Association of Local Authorities and Regions (SALAR) organized the task force in cooperation with the newly started Regional Cancer Centers of Sweden. A committee was formed with one committee member from each national center and professor Rolf Hultcrantz, the Principal Investigator of the present study, has been the chairman of the committee. The present study was designed based on what data was needed to get better outcome of colorectal cancer screening. The study design was submitted in January 2012 to the Ministry of Health and Social affairs and, since then, a thorough work has been carried out in order to get all counties in Sweden to fund the study. Eighteen counties in Sweden achieved the funding in March 2013 covering the actual screening procedures. The counties of Stockholm and Gotland cannot take part in the study due to the already implemented CRC-screening program with Hemoccult®.

2. Study aims

2.1 Primary endpoints

1. To demonstrate if colorectal cancer screening has an effect on the mortality from colorectal cancer in the Swedish population.
2. To demonstrate if colorectal cancer screening has an effect on the incidence of colorectal cancer in the Swedish population.
3. To demonstrate what method should be used in Sweden regarding the effect according to 1 and 2.

2.2 Secondary endpoints

1. To study compliance with the screening program (read study) and what factors are of importance for the adherence rate.
2. To study health economy and costs for colorectal cancer screening.
3. To study the emotional impact of screening on participants and non-participants including eventual change in lifestyle after invitation and/or participation.
4. To study quality control aspects and side effects of screening with colonoscopy.
5. To study pathology by means of quality registries and digital pathology.
6. To study surveillance strategies for adenomas found at colonoscopy.
7. To study associations of DNA in blood with findings at colonoscopy.
8. To study the flora of fecal bacteria among participants and outcome of FIT and colonoscopy.

3. Material and Methods

3.1 Study population

In total 200 000 individuals and residents of Sweden will be randomized from the population register maintained by Swedish tax agency (Skatteverket) (27). The randomized individuals will turn 60-years old the calendar year of randomization. Twenty thousand individuals will be invited to a primary screening colonoscopy, 60 000 individuals will be invited to high sensitive FIT and, if positive, to a subsequent colonoscopy and 120 000 persons will serve as controls (11. Figure 1 and 2). The inclusion period is set to three years with a repeated test after two years in the FIT arm.

3.1.1 Inclusion criteria

All individuals 60 years old and living in Sweden and randomized and identified through the Register of the total population.

3.1.2 Exclusion criteria

Residents of the counties of Stockholm and Gotland, individuals with a diagnose of colorectal cancer and/or anal cancer in the continuous updated cancer registers run by the local Regional Cancer Centers, individuals randomized to be included in the ongoing NordICC-trial (28) and individuals living in Västernorrland County – the only county in Sweden except for Stockholm/Gotland not participating in the study.

3.1.3 Randomization

From the Swedish tax agency three sets of individuals by random from the population register (27) will be asked for, approximately 67 000 individuals each born in 1954, 1955 and 1956. The randomization process will than be performed at the Head secretariat in the beginning of 2014, 2015 and 2016:

- 6 700 individuals will randomized, by county in proportion to the population, to be invited to primary colonoscopy (3.2.2 Colonoscopy).
- Three individuals per primary colonoscopy individual will be matched according to year of birth, gender and county of residence and invited to FIT (3.2.1 FIT).
- Six individuals per primary colonoscopy individual will be matched according to year of birth, gender and county of residence and serve as controls.

3.2 Invitation procedures

3.2.1 FIT

All individuals randomized to FIT as primary screening test will by regular mail receive an invitation to participate in the study. The invitation includes a brochure with information about the incentives of the study and the need for a follow-up colonoscopy in case of a positive test. The invitation contains a FIT-kit for two separate test samples and instructions on how to take the test. With the invitation is a pre-paid return-envelope for submitting the two tests together directly to the analyzing laboratory. The returned FIT will be stored in Bio bank after analysis.

3.2.2 Colonoscopy

All individuals randomized to the colonoscopy arm will receive an invitational letter by regular mail including the brochure with information about the incentives of the study. The invitee will be informed that they shortly by mail will receive an appointment for colonoscopy within 8 weeks, sent from the endoscopy clinic in their area of residency.

3.2.3 Controls

The individuals randomized to the control arm will not be contacted and informed about participation as controls in the study. If any individuals would contact the Head secretariat to ask about if they are controls, information will be given.

3.2.4 Undelivered invitations

All invitations will be sent by the Regional Cancer Center, Uppsala/Örebro, where the Head secretariat of SCREESCO is located. Undelivered and returned invitations will be sent out a second time, but if undelivered and returned again they will be excluded from the intention-to-screen analyses of the study (1.2.3 Compliance). Since allocation to intervention is by random, we believe the number of undelivered invitations will be approximately the same in both intervention arms.

3.3 Interventions

3.3.1 FIT-arm and the positivity rate

A high sensitive FIT (OC-Sensor®) with about 10% positivity rate will be used. The screening method of FIT is well known and established with an adjustable positivity rate from about 2% up to almost 10% (with and opposed effect on specificity). Almost all individuals have traces of blood and hereby hemoglobin in their feces, why the level of sensitivity of the test is an important balance of not missing any lesions, but at the same time minimizing the total number of false positive tests. In SCREESCO people are regarded as having a positive test result if at least one out of the two test samples are positive, i.e. above the set cut off level of 50 µg Hb/L (50 ng Hb/mL). With the used OC-Sensor® 1 µg Hb/L buffer = 0,2 µg Hb/g feces, *i.e.* 50 µg Hb/L buffer equals 10 µg Hb/g feces.

3.3.1.1 Two rounds of FIT

We plan two rounds of FIT – one the first year and one the third year. All individuals randomized to the FIT-arm will have new test-kits sent home after two years, regardless of compliance with the invitation to the first round. Previous studies have been performed with FOBT every second year for ten years and we will follow the findings and perform interim analyses and suggest that further rounds of FIT are carried out if the scientific committee deems it necessary. Two tests per round will be asked for without dietary restrictions. If 60% of the invitees comply and send their FIT test to the laboratory (experiences from the ongoing screening program in the counties of Stockholm and Gotland) and 10% are positive, this will generate approximately 1 200 colonoscopies for each year in the FIT-arm except for year four when the number of generated colonoscopies is estimated to 2 400 due to the second round re-testing.

3.3.1.2 Information of FIT-result
All participating individuals in the FIT-arm will be informed about their test result by mail within 4 weeks after the test was sent in. People with a negative test result will in the same mail be informed that a new test-kit will be sent after two years. People with positive test will be informed by mail that they within one week will get a colonoscopy appointment (within 4 weeks instead of 8 weeks as in primary endoscopy arm [3.3.2.1] due to positive test) by mail sent by the endoscopy site in their area of residency.

3.3.1.3 Reminder and default returned FIT
People with no tests sent in will get a reminder by mail after 8 weeks and with instructions to call to receive new test kits if they are missing. People with default FITs sent in will receive new test kits with instructions.

3.3.1.4 FIT returned after 6 months
FIT returned after 6 months will be scientifically handled as non-compliers. The test results are expected un-valid due to expiring date of the test.

3.3.1.5 Follow-up colonoscopy appointment of individuals with positive FIT
Within one week after the information of a positive test is sent to the participant, the endoscopy site corresponding to the participants geographical area of residency will electronically within the Central IT-Support System – Study (CIS-S) (6. Data management) receive the information about the participant that needs an appointment for colonoscopy and book one.

3.3.1.6 Bowel preparation for follow-up colonoscopy
Instructions to call and confirm the colonoscopy appointment within the 4 weeks is included with the mailed colonoscopy appointment and after confirmation the participant with a positive FIT will receive bowel preparation (Laxabon®) by mail without charge.

3.3.1.7 Medical history taken at telephone confirmation of appointment
At confirmation of the colonoscopy appointment the study nurse will ask a few specific questions about the health status of the invitee, including medications (*e.g.* Warfarin®) and check with the responsible physician in doubt of colonoscopy risks. The invitee will also at the telephone conversation be able to ask questions about the colonoscopy.

3.3.1.8 Reminder of follow-up colonoscopy
Without confirmation by telephone a reminder together with a new colonoscopy appointment time is sent out from the corresponding endoscopy site, following the same confirmation procedure as above.

3.3.1.9 Follow-up after positive FIT follow-up colonoscopy
Individuals with no pathological finding at colonoscopy will be invited to a second FIT after two years and individuals with a pathological finding will be followed-up regarding to the specific clinical guidelines.

3.1.1.9.10 Positive FIT and asking for a new FIT replacing colonoscopy
Individuals asking for a new FIT instead of a follow-up colonoscopy after a positive test will not have this option. In the study, all positive FIT will be followed-up by a colonoscopy. All individuals invited to the FIT-arm will have a second round of FIT sent home after two years.

3.3.2 Colonoscopy-arm
A primary screening colonoscopy will be carried out once and with an estimated adherence of about 50%, approximately 10 000 colonoscopies will be carried out. The examination will be performed with or without sedation following a standard bowel cleaning preparation. The endoscopy centers will be accredited and the quality of the examiners will be investigated. All details will follow the European guidelines (26, 29).

3.3.2.1 Primary colonoscopy appointment
With the colonoscopy appointment (within 8 weeks) sent after the initial invitation letter are instructions to call and confirm the appointment, following the same procedure as the participants with a positive FIT (3.3.1.6 Bowel preparation for follow-up colonoscopy). A difference, though, is that in the group randomized to primary colonoscopy screening, a reminder will be sent out from the CIS-S if no confirmation of the colonoscopy appointment has been performed within 8 weeks. The endoscopy site will then send a new colonoscopy appointment (within 8 weeks) to be confirmed as previously described.

3.3.2.2 Weight and height measurement and nurse questionnaire
At both primary colonoscopy and follow-up colonoscopy after a positive FIT, the nurse will weigh and measure height (not only asked for) and ask up to ten health questions regarding *e.g.* smoking, alcohol and use of non-steroid anti-inflammatory drugs to be registered in the CIS-S. The questions will be asked *before* the colonoscopy to limit re-call bias.

3.3.2.3 Blood sample at colonoscopy
At colonoscopy the participant will be asked to leave two blood samples (EDTA®) to be stored in Bio bank for future analyses after signing an informed consent (3.4 Informed consent). The individual can decline leaving blood samples for the study, but participate with colonoscopy.

3.3.2.4 Positive finding at colonoscopy
Individuals with pathological finding at colonoscopy, *i.e.* colorectal cancer or advanced adenomas qualifying to the adenoma surveillance program, will be followed-up regarding to the specific local clinical guidelines. No further screening test will be offered within the study for this category of patients. Patients with *other* findings at colonoscopy, *e.g.* inflammatory bowel disease, will be taken care of by the performing endoscopist, but not excluded from a second round of FIT-screening within the SCREESCO-study if randomized to the FIT intervention arm.

3.3.2.5 Individuals with positive FIT but negative colonoscopy
Individuals with a positive FIT but with a negative colonoscopy (no adenoma or colorectal cancer) will not be investigated further within the study, accept for a second round of FIT after two years if randomized to the FIT intervention arm.

3.3.2.6 Questionnaire after the colonoscopy
After the colonoscopy the participant will receive a short questionnaire about there experiences of the invitation to screening, bowel preparation and colonoscopy examination, together with a pre paid return envelope addressed to the Head secretariat. The participant will be asked to fill out and post the questionnaire within 24 hours. The questionnaires are confidential, but are linked to the colonoscopy protocols to enable to relate answers with, *e.g.*, intervention arm (primary- *vs.* follow-up colonoscopy) and possible findings.

3.4 Informed consent
The SCREESCO-study has approval from the Ethical Review Board (No. 2012/2058-31/3) Stockholm, Sweden, that a returned FIT-test is to be regarded as informed consent to participate in the study. At colonoscopy, an informed consent is signed by both the study participant in the primary intervention arm, the individuals with a positive FIT and the endoscopist informing about the procedure. The informed consents will be stored at the endoscopy site and returned to the Head secretariat of SCREESCO at the end of the inclusion period.

3.5 Quality assurance of colonoscopy
A specific part of the study will be aimed at controlling the outcome of the estimated 17 000 colonoscopies. The patients will be subjected to colonoscopy in 37 different centers throughout Sweden in the 13 participating counties. Previous work in this field has demonstrated that a good quality endoscopist should perform more than 300 procedures/year and be able to detect adenomas in more than 20 % of the examinations. Moreover, following intubation of the instrument to the caecum, the withdrawal time should be more than six minutes.

There will be a thorough evaluation of each endoscopist from a quality aspect according to set guidelines. This will be carried out by using a specific quality register in the CIS-S, where data on the success rate of the colonoscopist, findings and side effects will be entered from all the estimated 17 000 colonoscopies. In the study registry, adverse advents such as pain, bleeding and perforations will also be collected. The register data will continuously be cross-linked with data from the National Patient register (30) and the Swedish Causes of Death register (31) in order to find severe adverse events.

The colonoscopy performance register will contain a unique set of data, which could be used for both colonoscopy development as well as the follow-up of the safety and success in a screening setting – enabling correlation of the performance regarding findings and adverse events of each endoscopist and the previous described quality indicators. The results will be stratified by different categories of endoscopists, those with high and medium numbers of procedures annually, with special focus on nurses performing endoscopies since involving

nurses in the endoscopies may be an important step and a key to a successful CRC-screening program in the country.

An internet-based on-line system to connect one endoscopist with a set of experts will be developed within the study in order to deliver immediate second opinion of findings during a procedure. Criteria for the identification of adenomatous polyps as opposed to hyperplastic polyps (32) during the endoscopy will also be developed in the study. Hyperplastic polyps are unlikely to develop dysplasia and, hence, do not have to be removed. If they can be identified in the endoscope much work is spared and patient safety is improved.

Moreover, both primary screening colonoscopies and follow-up colonoscopies after a positive FIT test will be evaluated with a participant questionnaire (3.3.2.6 Questionnaire after the colonoscopy).

3.6 Pathology

All removed adenomatous polyps will be sent for histopathology and also bio banked for further analyses. Approximately 11 000 adenomas will be removed during the study. A new technique for digitalization of sections from adenomas larger than 10 mm will be developed. Digital images will be made in Aperio machines from sections of adenomas and stored. These images will be used for quality studies in order to develop processes for diagnostic procedures in pathology in colorectal screening. Secondary studies on correlation of evaluation between various pathologists will be performed in order to create kappa-values in collaboration with Swedish gastrointestinal pathologists in the KVAST (Kvalitets- och standardiseringskommittén [in Swedish]) Study Group of the Swedish Society for Pathology (33). The data will be used in order to demonstrate which type of adenomas need to be extra carefully resected and which of them are especially at risk to develop new adenomas. The purpose is to combine these results with the results from the colonoscopical investigation in order to possibly reduce the number of polyps needed to be resected generating a decreased risk for perforation of the colonic wall by the procedure and also a reduced work-load for both endoscopists and pathologists (32).

3.7 Secondary studies

3.7.1 Complier and non-complier experiences
3.7.2 Health-economy
3.7.3 Quality assurance
3.7.4 Surveillance of adenomas
3.7.5 DNA in blood
3.7.6 Bacteria in feces
3.7.7 Non-steroid anti-inflammatory drugs and adenoma development

3.8 Follow-up

Follow-up time is set at 15 years after inclusion and invitation for the primary endpoints colorectal cancer mortality and incidence with interim analysis at 5 and 10 years. Data of all 200.000 individuals randomized from the population register (FIT-, primary colonoscopy- or control arm) will be obtained from the Causes of Death register (31) and the Cancer register (1) managed by the

Swedish National Board of Health and Welfare. Regarding secondary outcomes, *e.g.* quality control of performed colonoscopies and non-steroid anti-inflammatory drugs and adenoma development, information will be retrieved from registries such as the Patient register (30) and the Drug register (34).

4. Ethical considerations

To randomize people from the population register and invite them to screening colonoscopy (or not when control) is an ethical challenge. Primarily, we do have to consider possible risks for the participants, *i.e.* side effects of the primary and follow-up colonoscopies, but secondarily, we also have to consider the stress a false positive test could generate. Further more, we will cross link register information of individuals randomized as controls without their informed consent and the information generated must be treated rigorously and that is why the controls are de-identified and the register information gathered aggregated at group level. On a population level, it is of utter most importance that the study is performed. Most certainly, due to the increasing frequency of opportunistic screening, we only have one chance to get a solid answer to our primary endpoint - to demonstrate if colorectal cancer screening has an effect on the mortality from colorectal cancer in the Swedish population. The study has been processed and approved by the regional Ethics Review Board at Karolinska Institutet, Stockholm, Sweden (No. 2012/2058-31/3).

5. Statistical analyses

All individuals will be randomized and allocated to one of three arms; FIT, colonoscopy or control. Disease specific mortality is the variable used for power analysis. Individuals registered in the national Register of the total population will be the bases for the intention to screen analysis and the follow up period will be 15 years.

The lifetime cumulative mortality in colorectal cancer in Sweden is about 1% after 15 years. With a 80% power and a 2.5% significance level according to the Bonferroni method the present study need to randomize 20 100 persons in the colonoscopy arm, 60 186 in the FIT arm and 120 372 in the control arm.
Based on previous studies, we estimate that the reduction in mortality will be about 30% for those examined with FIT and a subsequent colonoscopy if the FIT is positive and approximately 50% for individuals who are examined with a primary screening colonoscopy. The compliance is estimated to about 60% in the FIT-arm and approximately 10% of them will have a positive test and invited to follow-up colonoscopy with 80-90% adherence rate. Compliance with primary colonoscopy is estimated to 50% and there is supposed to be a very low contamination from opportunistic screening.

6. Data management

The Central IT-Support System – Study (CIS-S) platform will be located at the Head secretariat. All information generated by the invitation routines, laboratory tests and findings at colonoscopy, as well as individual questionnaire information, will be automatically registered in the system prospectively and available with explicit restriction to guarantee discretion of personal information of individuals.

7. Head secretariat

The Head secretariat of the study is situated at the Regional Cancer Centre in the Uppsala/Örebro region, Uppsala, Sweden.

http://www.cancercentrum.se/sv/uppsalaorebro/Aktuellt/Nyheter/tarmcancer screeningstudie-startar-forsta-kvartalet-2014/

http://www.cancercentrum.se/sv/uppsalaorebro/Funktioner/in-english/

8. Participating centers

There are thirty-seven participating endoscopy sites distributed nationally and in the areas of residency of the invitees (and controls) of the study.

9. Main study publications

Descriptive paper of the screening study (methods and rationale).
Compliance, findings and negative effects of the screening initiative.
Emotional impact of screening on participants and non-participants.
Quality assurance of screening colonoscopy and pathology reporting.
Health economy of colorectal cancer screening implementation.
Follow-up analyses regarding primary end-point at 10 and 15 years.
Secondary studies (3.7 Secondary studies).

10. Scientific committee

Name	Country	Area of expertise
Rolf Hultcrantz	Sweden	Gastroenterology
Lars Holmberg	Sweden	Screening, surgery
Anders Ekbom	Sweden	Epidemiology
Robert Steele	United Kingdom	Surgery
Richard Palmqvist	Sweden	Pathology
Mef Nilbert	Sweden	Molecular oncology
Andreas Pischel	Sweden	Endoscopy
Marc Bruse	Belgium	Biostatistics
Yvonne Wengström	Sweden	Qualitative research
Per Carlsson	Sweden	Health economy
Lars Engstrand	Sweden	Microbiota
Johannes Blom	Sweden	Screening, surgery

11. Figures

11.1 Figure 1. Flow chart of invitation procedure of individuals randomized to intervention FIT

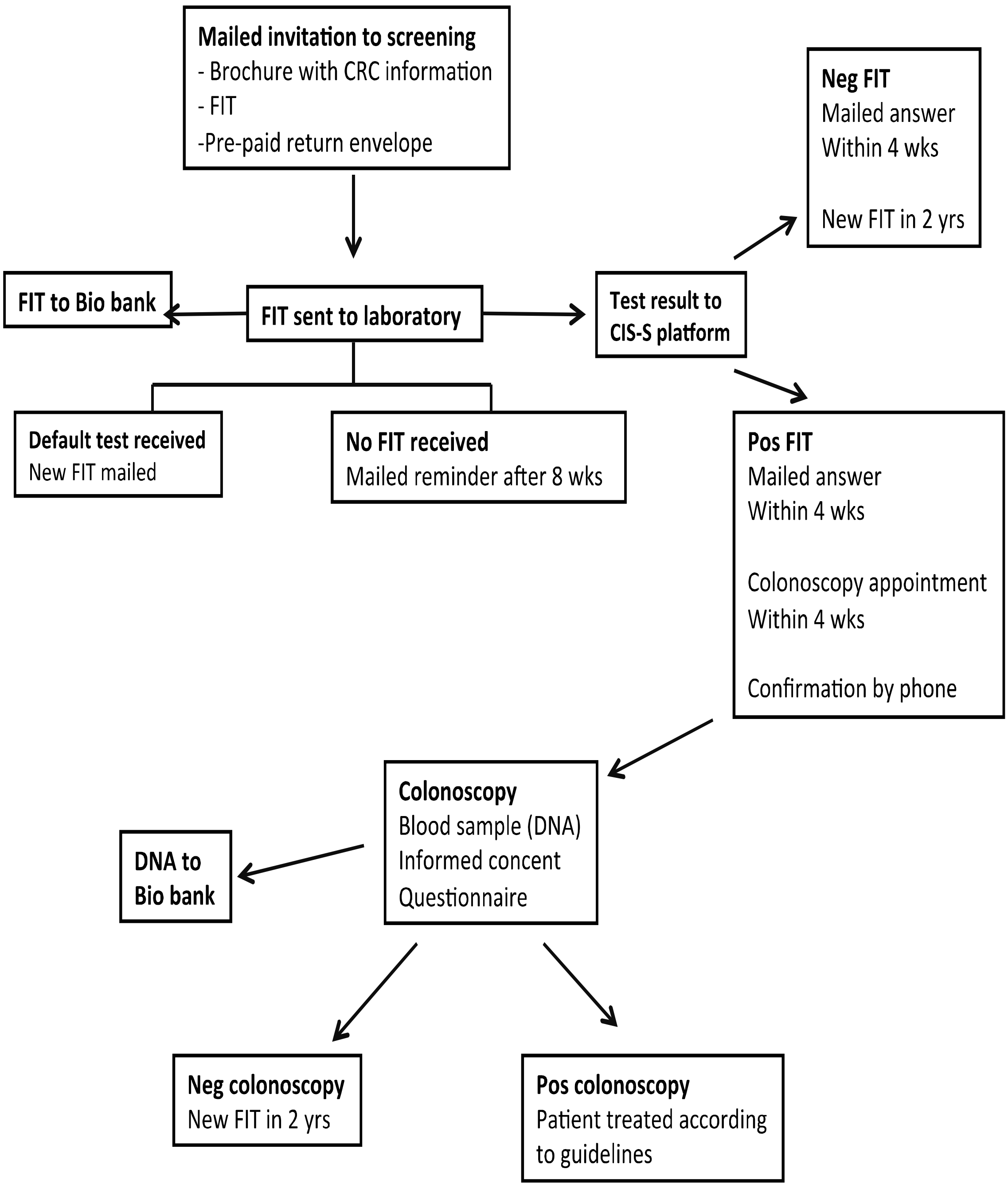

11.2 Figure 2. Flow chart of invitation procedure of individuals randomized to intervention primary colonoscopy

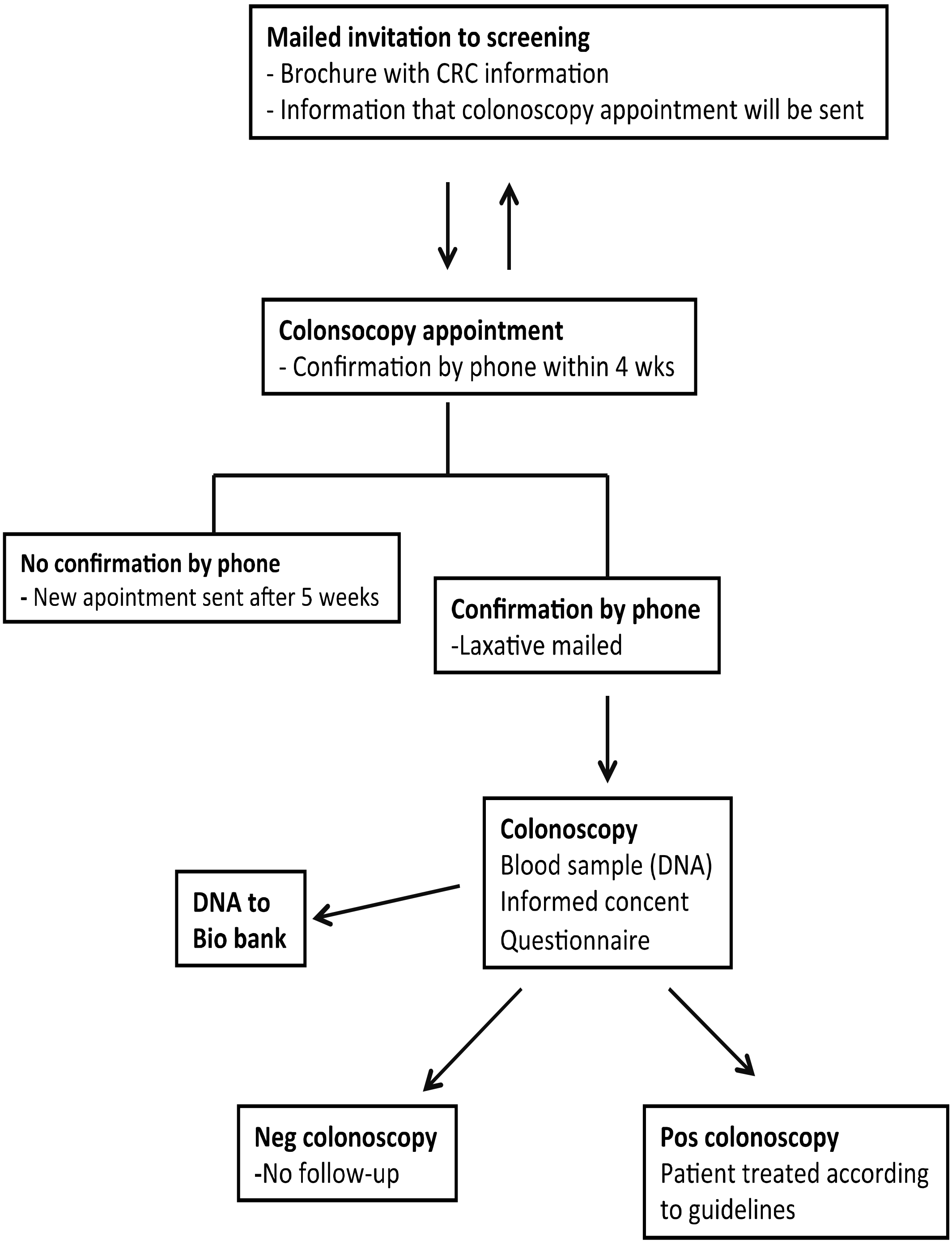

12. References

1. Cancer register http://www.socialstyrelsen.se/register/halsodataregister/cancerregistret/inenglish: National Board of Health and Welfare; [cited 2013 December].
2. Muto T, Bussey HJ, Morson BC. The evolution of cancer of the colon and rectum. Cancer. 1975 Dec;36(6):2251-70. PubMed PMID: 1203876.
3. Vogelstein B, Fearon ER, Hamilton SR, Kern SE, Preisinger AC, Leppert M, et al. Genetic alterations during colorectal-tumor development. N Engl J Med. 1988 Sep 1;319(9):525-32. PubMed PMID: 2841597.
4. Winawer SJ, Zauber AG, Ho MN, O'Brien MJ, Gottlieb LS, Sternberg SS, et al. Prevention of colorectal cancer by colonoscopic polypectomy. The National Polyp Study Workgroup. N Engl J Med. 1993 Dec 30;329(27):1977-81. PubMed PMID: 8247072.
5. Muller AD, Sonnenberg A. Prevention of colorectal cancer by flexible endoscopy and polypectomy. A case-control study of 32,702 veterans. Ann Intern Med. 1995 Dec 15;123(12):904-10. PubMed PMID: 7486484.
6. Thiis-Evensen E, Hoff GS, Sauar J, Langmark F, Majak BM, Vatn MH. Population-based surveillance by colonoscopy: effect on the incidence of colorectal cancer. Telemark Polyp Study I. Scand J Gastroenterol. 1999 Apr;34(4):414-20. PubMed PMID: 10365903.
7. Armitage N. Screening for cancer. In: Morris D, Kearsley, J, Williams, C, editor. Cancer: a comprehensive clnical guide. pp 9-20. U.K.: Taylor and Francis Ltd 1997.
8. Mandel JS, Bond JH, Church TR, Snover DC, Bradley GM, Schuman LM, et al. Reducing mortality from colorectal cancer by screening for fecal occult blood. Minnesota Colon Cancer Control Study. N Engl J Med. 1993 May 13;328(19):1365-71. PubMed PMID: 8474513.
9. Kronborg O, Fenger C, Olsen J, Jorgensen OD, Sondergaard O. Randomised study of screening for colorectal cancer with faecal-occult-blood test. Lancet. 1996 Nov 30;348(9040):1467-71. PubMed PMID: 8942774.
10. Hardcastle JD, Chamberlain JO, Robinson MH, Moss SM, Amar SS, Balfour TW, et al. Randomised controlled trial of faecal-occult-blood screening for colorectal cancer. Lancet. 1996 Nov 30;348(9040):1472-7. PubMed PMID: 8942775.
11. Lindholm E, Brevinge H, Haglind E. Survival benefit in a randomized clinical trial of faecal occult blood screening for colorectal cancer. Br J Surg. 2008 Aug;95(8):1029-36. PubMed PMID: 18563785.
12. Atkin WS, Edwards R, Kralj-Hans I, Wooldrage K, Hart AR, Northover JM, et al. Once-only flexible sigmoidoscopy screening in prevention of colorectal cancer: a multicentre randomised controlled trial. Lancet. 2010 May 8;375(9726):1624-33. PubMed PMID: 20430429.
13. Segnan N, Armaroli P, Bonelli L, Risio M, Sciallero S, Zappa M, et al. Once-only sigmoidoscopy in colorectal cancer screening: follow-up findings of the Italian Randomized Controlled Trial--SCORE. J Natl Cancer Inst. 2011 Sep 7;103(17):1310-22. PubMed PMID: 21852264.
14. Schoen RE, Pinsky PF, Weissfeld JL, Yokochi LA, Church T, Laiyemo AO, et al. Colorectal-cancer incidence and mortality with screening flexible sigmoidoscopy. N Engl J Med. 2012 Jun 21;366(25):2345-57. PubMed PMID: 22612596. Pubmed Central PMCID: 3641846.

15. Faivre J, Bouvier AM, Bonithon-Kopp C. Epidemiology and screening of colorectal cancer. Best Pract Res Clin Gastroenterol. 2002 Apr;16(2):187-99. PubMed PMID: 11969233.
16. Richardson A. Screening and the number needed to treat. J Med Screen. 2001;8(3):125-7. PubMed PMID: 11678550.
17. Bretthauer M. Colorectal cancer screening. J Intern Med. 2011 Aug;270(2):87-98. PubMed PMID: 21575082.
18. Brouwers MC, De Vito C, Bahirathan L, Carol A, Carroll JC, Cotterchio M, et al. What implementation interventions increase cancer screening rates? a systematic review. Implementation science : IS. 2011;6:111. PubMed PMID: 21958556. Pubmed Central PMCID: 3197548.
19. Tinmouth J, Ritvo P, McGregor SE, Claus D, Pasut G, Myers RE, et al. A qualitative evaluation of strategies to increase colorectal cancer screening uptake. Can Fam Physician. 2011 Jan;57(1):e7-15. PubMed PMID: 21322288. Pubmed Central PMCID: 3024174.
20. Blom J, Liden A, Jeppsson B, Holmberg L, Pahlman L. Compliance and findings in a Swedish population screened for colorectal cancer with sigmoidoscopy. Eur J Surg Oncol. 2002 Dec;28(8):827-31. PubMed PMID: 12477473.
21. Blom J, Liden A, Nilsson J, Pahlman L, Nyren O, Holmberg L. Colorectal cancer screening with flexible sigmoidoscopy-participants' experiences and technical feasibility. Eur J Surg Oncol. 2004 May;30(4):362-9. PubMed PMID: 15063888.
22. Collins RE, Lopez LM, Marteau TM. Emotional impact of screening: a systematic review and meta-analysis. BMC Public Health. 2011;11:603. PubMed PMID: 21798046. Pubmed Central PMCID: 3223929.
23. Tornberg S, Lundstrom V, Gustafsson S, Hultkrantz R. [The first year with colorectal cancer screening in Stockholm. Careful monitoring and quality control of the whole process is necessary]. Lakartidningen. 2010 Jun 30-Jul 20;107(26-28):1709-11. PubMed PMID: 20701152. Forsta aret med screening for kolorektal cancer i Stockholm. Noggrann overvakning och kvalitetskontroll av hela vardkedjan nodvandig.
24. Foss C, Ellefsen B. The value of combining qualitative and quantitative approaches in nursing research by means of method triangulation. J Adv Nurs. 2002 Oct;40(2):242-8. PubMed PMID: 12366654.
25. Ostlund U, Kidd L, Wengstrom Y, Rowa-Dewar N. Combining qualitative and quantitative research within mixed method research designs: a methodological review. Int J Nurs Stud. 2011 Mar;48(3):369-83. PubMed PMID: 21084086.
26. von Karsa L, Patnick J, Segnan N. European guidelines for quality assurance in colorectal cancer screening and diagnosis. First Edition--Executive summary. Endoscopy. 2012 Sep;44 Suppl 3:SE1-8. PubMed PMID: 23012113.
27. Register of the total population https://http://www.skatteverket.se/download/18.2b543913a42158acf80003761/1386594730871/Allm%C3%A4nBeskrivning.pdf: Swedish tax agency; [cited 2013 December].
28. Kaminski MF, Bretthauer M, Zauber AG, Kuipers EJ, Adami HO, van Ballegooijen M, et al. The NordICC Study: rationale and design of a randomized

trial on colonoscopy screening for colorectal cancer. Endoscopy. 2012 Jul;44(7):695-702. PubMed PMID: 22723185.
29. Rembacken B, Hassan C, Riemann JF, Chilton A, Rutter M, Dumonceau JM, et al. Quality in screening colonoscopy: position statement of the European Society of Gastrointestinal Endoscopy (ESGE). Endoscopy. 2012 Oct;44(10):957-68. PubMed PMID: 22987217.
30. Patient register http://www.socialstyrelsen.se/register/halsodataregister/patientregistret/inenglish: National Board of Health and Welfare; [cited 2013 December].
31. Causes of Death register http://www.socialstyrelsen.se/register/dodsorsaksregistret: National Board of Health and Welfare; [cited 2013 December].
32. Wallace MB, Kiesslich R. Advances in endoscopic imaging of colorectal neoplasia. Gastroenterology. 2010 Jun;138(6):2140-50. PubMed PMID: 20420951.
33. Hamilton SR AL. Pathology and Genetics. Tumours of the Digestive System, WHO Classification of tumours, vol. 2. IARC WHO, Classification of tumours, No 2; 20.
34. Drug register http://www.socialstyrelsen.se/register/halsodataregister/lakemedelsregistret National Board of Health and Welfare; [cited 2013 December].

SCREESCO – Screening of Swedish Colons 2.0

Study Protocol

131220

Amended study protocol after new power-calculation due to anticipated 35% participation in the colonoscopy arm. In Swedish 2017-03-10, translated to English 2021-04-29

Summary

Colorectal cancer (CRC) is a major cause of death in Sweden. There are approximately 6000 new cases each year in Sweden and the disease specific mortality is more than 40%. The risk is about 1% to develop CRC between 60-70 years of age, making 60-year-olds a suitable target population for colorectal cancer screening.

The Swedish Ministry of Health and Social affairs has proposed a national study on the efficiency of colorectal cancer screening in the Swedish population regarding mortality, but also what screening method to be used. Thirteen participating counties of Sweden now fund the study to be launched in 2014.

Individuals 60 years of age will be randomized from the population register and invited to screening by mail. 31,140 individuals will be invited to primary colonoscopy and 60,300 individuals will be invited to high-sensitive FIT (OC Sensor®) with approximately 10% positivity rate and, if positive, to a subsequent follow-up colonoscopy. When test negative a second round of FIT will be asked for in two years. In total 186,840 randomized individuals will not be invited to screening serve as controls and will be followed in the Swedish Cancer Register. The inclusion period is set to five years (five years including the second round of FIT) generating approximately 17,000 colonoscopies at a compliance rate of 35% in the colonoscopy arm and 50% in the FIT arm.

Follow-up time is set to 15 years with the primary endpoint disease specific mortality and colorectal cancer incidence. Secondary outcomes, by others, to be studied are in short quality assurance variables of colonoscopy, participants and non-participants experiences of the invitation and the screening procedure, health economy measures of the CRC-screening study and when implemented in clinical care.

Table of contents

1. Background

1.1 Colorectal cancer and screening

1.2 Evaluation of a screening program

1.2.1 Effectiveness

1.2.2 The screening test

1.2.3 Compliance

1.2.4 Cost-effectiveness

1.3 Challenges in colorectal cancer screening

1.3.1 Colonoscopy quality assurance

1.3.2 Lack of pathologists

1.3.3 Compliance and emotional effects

1.4 Colorectal cancer screening in Sweden

1.5 A call for a population-based Swedish CRC-screening study

2. Study aims

2.1 Primary endpoints

2.2 Secondary endpoints

3. Material and Methods

3.1 Study population

3.1.1 Inclusion criteria

3.1.2 Exclusion criteria

3.1.3 Randomization

3.2 Invitation procedures

3.2.1 Colonoscopy

3.2.2 FIT

3.2.3 Controls

3.2.4 Undelivered invitations

3.3 Interventions

3.3.1 Colonoscopy-arm

3.3.2. Primary colonoscopy appointment

3.3.3 FIT arm and the positivity rate

3.3.4 *Two rounds of FIT*

3.3.5 *Information of FIT-result*

3.3.6 *Reminder and default returned FIT*

3.3.7 *FIT returned after 6 months*

3.3.8 *Follow-up colonoscopy appointment of individuals with positive FIT*

3.3.9 *Bowel preparation for follow-up colonoscopy*

3.3.10 *Medical history taken at telephone confirmation of appointment*

3.3.11 *Reminder of follow-up colonoscopy*

3.3.12 *Follow-up after positive FIT follow-up colonoscopy*

3.3.13 *Positive FIT and asking for a new FIT replacing colonoscopy*

3.3.14 *Weight and height measurement and nurse questionnaire*

3.3.15 *Blood sample at colonoscopy*

3.3.16 *Positive finding at colonoscopy*

3.3.17 *Individuals with positive FIT but negative colonoscopy*

3.3.18 *Questionnaire after the colonoscopy*

3.4 Informed consent

3.5 Quality assurance of colonoscopy

3.6 Pathology

3.7 Secondary studies

3.7.1 Complier and non-complier experiences

3.7.2 Health-economy

3.7.3 Quality assurance

3.7.4 Surveillance of adenomas

3.7.5 DNA in blood

3.7.6 Bacteria in feces

3.7.7 Non-steroid anti-inflammatory drugs and adenoma development

3.8 Follow-up

4. Ethical considerations

5. Statistical analyses

6. Data management

7. Head secretariat

8. Participating centers

9. Main study publications

10. Scientific committee

11. Figures

11.1 Figure 1. Flow chart of invitation procedure of individuals randomized to intervention FIT

11.2 Figure 2. Flow chart of invitation procedure of individuals randomized to intervention primary colonoscopy

12. References

1. Background

1.1 Colorectal cancer and screening

The primary purpose of cancer screening is to reduce mortality from the disease in the population by finding the cancer at an early and treatable stage. The cancer has to be an important health problem to be a suitable target for screening. With approximately 6 000 new cases each year in Sweden (1), colorectal cancer (CRC) is the third most common cancer in Sweden (after prostate- and breast cancer) and, hence, a relatively common disease, but it is also a major cause of death. There is a 1% risk to develop CRC between the ages 60-75 years, and the five-year survival rate is close to 60%. The prognosis is related to if the cancer is detected at an early or late stage of the disease. When detected at an early stage, there is a 90% five-year survival, as compared to only a 10% five-year survival if the cancer at detected at a late stage.

CRC is usually detected clinically by patient symptoms, either an alteration in bowel habits due to obstruction of the lumen, visual blood in the stool or symptoms due to anemia caused by a bleeding from the tumor. Both larger precursor stages – the adenomatous polyp (2, 3) – and cancers bleed and could be detected by sensitive methods to analyze blood in the stool. All patients diagnosed with colorectal cancer need treatment, but there is a significant difference in suffers and costs depending on the stage the disease at diagnosis. With screening early stages of the disease will be found before they are clinically detected.

Removal of adenomatous polyps (adenomas) has a protective effect against colorectal cancer development (4-6) and, consequentially, a colorectal cancer screening program also might have the potential of decreasing the future incidence of the disease.

1.2 Evaluation of a screening program

1.2.1 Effectiveness

The effectiveness of a screening program is the ability of the program to reduce the disease specific mortality. Survival is not a valid measure of effectiveness because of the possibilities of bias; selection bias (when screened subjects and non-screened controls represent different populations), lead time bias (earlier diagnosis in screen-detected cancers adds time to the total survival time) and length biased sampling (screen-detected tumors often grow slowly and might be less malignant).

The most valid measure of effectiveness in screening is a lower mortality in the screened group, as compared to the non-screened group evaluated in a randomized controlled trial (RCT):

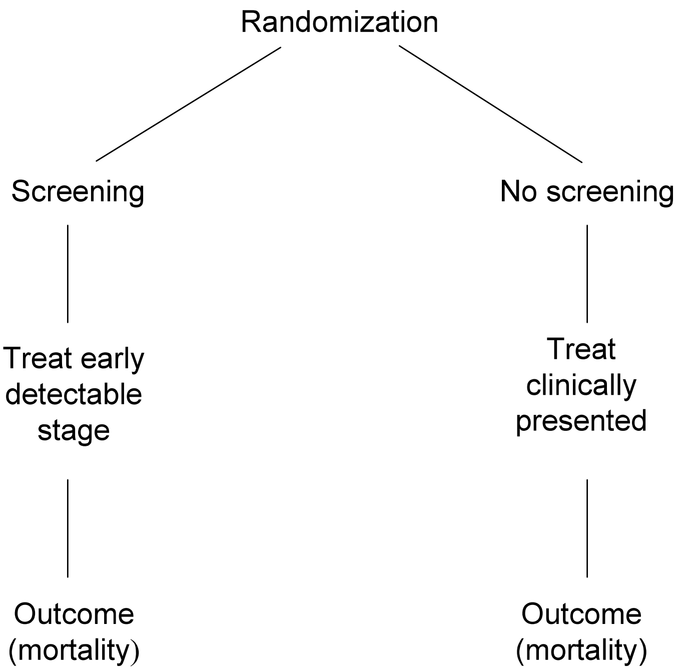

1.2.2 The screening test

A very large majority of individuals in the general population using the screening test offered will not have colorectal cancer. Therefore the test must be free from unwanted side effects, inexpensive, but also simple to take and easy to interpret (7). Furthermore, a high sensitivity (to limit the number of missed cancers) and specificity (to limit the number of incorrect diagnosed cancers), is a prerequisite for a screening test to be used in a program of the average risk population.

There are a number of screening tests and methods to examine the colorectum in order to find CRC and/or adenomatous polyps:

Indirect tests: The most commonly used screening test has been the guaiac based fecal occult blood test (FOBT) (Hemoccult®). Four larger RCTs have demonstrated a 16% decreased in CRC mortality with the test in screening (8-11). The degree of mortality reduction with Hemoccult® depends on the compliance with the test and the dietary restrictions, the sensitivity of the test, the screening frequency (annual or biennial), the number of screening rounds the subjects participate in and, also, the compliance with the diagnostic follow-up colonoscopy after a positive test. More advanced fecal immunological tests (FITs) with higher sensitivity, but only a marginally increase in the false positive rate (specificity), are now available. FIT demonstrates presence of human blood only, as opposed to the guaiac test that also can be positive due to animal hemoglobin. Moreover, no dietary restrictions are needed with FIT.

Direct tests: The main advantages with endoscopy (*e.g.* colonoscopy and sigmoidoscopy) are the direct visualization of the colorectum and the possibility of obtaining tissue samples from suspected cancer lesions for histopathology and/or removing adenomatous polyps during the procedure. Suggestively, the bowel can be examined with sigmoidoscopy and a subsequent colonoscopy in case of pathological finding within the reach of the sigmoidoscope (approximately 60 cm) or with a complete colonoscopy as primary test (when 1/3 of tumors will appear in the right part of the colon). Recently, three randomized controlled trials with sigmoidoscopy as the primary screening test have demonstrated both a reduced disease specific mortality of around 30%, but also a reduced incidence of CRC of as much as 40% (12-14).

Up to now, there are no larger randomized controlled studies of the average risk population published demonstrating a disease specific mortality reduction with colonoscopy as the primary screening test.

1.2.3 Compliance

The proportion of individuals offered a screening test and actually take the test is referred to as compliance (7). The compliance to a screening program is a major determinant of the program's effectiveness and there has to be a rigid organization with a call- recall system and quality assurance in a screening program to be effective (15).

Instead of number needed to treat (NNT) used to estimate the efficacy in interventional RCTs evaluating medication, the number needed to screen (NNS) is used in the evaluation of RCTs of screening. The NNS is the number of individuals who need to be invited (offered) screening to prevent one death (intention-to-screen). The results then reflect the efficacy of the screening program to reduce mortality among those *invited* to screening. With NNS there is often an underestimation of the efficacy of the screening test in those people who actually participate – are being screened. The NNS for most screening programs are usually much higher than the number of people who have to participate to prevent one death (16). It is only the participants that can contribute to the mortality reduction achieved by the screening program and with low compliance the number of deaths prevented will be few, and consequently the NNS will be very large (16). Therefore, a high participation rate in a screening program is very important to be able to evaluate its effectiveness on mortality.

1.2.4 Cost-effectiveness

There are a large variety of variables involved when measuring the cost-effectiveness of a colorectal cancer screening program and focus cannot only be on the eventual incidence and mortality reduction of the disease. Firstly, one havs to make assumptions about the duration of the early, asymptomatic and curable stage of the disease. Secondly, one has to estimate the effectiveness and

negative effects of the screening procedure, such as morbidity due to complications and costs. A low compliance in a screening program will both effect the incidence and mortality reduction achieved and the cost-effectiveness of the program.

1.3 Challenges in colorectal cancer screening

1.3.1 Colonoscopy quality assurance

There are some outstanding challenges in implementing colorectal cancer screening for the average risk population. One very important issue is the quality assurance of the endoscopic examinations and the follow-up of eventual findings. If the quality of the examinations is not excellent, but with frequent practical mistakes and neglect to find adenomas and or cancers, the positive effect of the screening procedure for that particular individual and of the program as a whole is both diminished and unethical (17-21).

Colonoscopy resources and quality will be a key issue in the present study. Currently in Sweden, about 65 000 procedures are carried out outside the Stockholm area yearly, while approximately 30 000 are carried out in Stockholm. A crude estimate is that a future national screening program will need a substantial number of colonoscopies, and, thus, much work is needed to both increase the number of colonoscopies performed nationally and also increase and secure the quality of the investigations.

Adenomatous polyps should be removed in order to prevent the occurrence of CRC. The removal of adenomas is a sometimes difficult and dangerous procedure, since it may cause bleeding and perforation of the bowel wall. It is very important that the adenoma is completely removed and correctly diagnosed. Thus, both a skillful endoscopists and high-quality histopathology are extremely important.

1.3.2 Lack of pathologists

Another problem when implementing a screening program is the created increased burden for pathologists. There is a need for standardization of the use of the diagnostic criteria and the possibility to have a group of pathologists to evaluate the sections. In order to do so, it would be of great advantage if the histological sections could be stained and digitally processed and evaluated electronically.

1.3.3 Compliance and emotional effects

Another important factor, in need of further studies, is the actual adherence rate or compliance discussed above and the emotional effects of the inclusion of healthy persons from the general population in colorectal cancer screening (22-25).

1.4 Colorectal cancer screening in Sweden

The National Board of Health and Welfare has not been recommending CRC screening due to lack of experiences of screening in routine health care and refer CRC screening to the Research and

Development list waiting for studies outside Sweden to generate results. Based on the results from the four RCTs with a net CRC-mortality reduction of 16% (8-11) the Council of the European Union in 2003 recommended CRC-screening with guaiac-based fecal occult blood test (FOBT) in the average risk population aged 55-74 years old. The recommendations have recently been updated in the European Guidelines of Quality Assurance in CRC-screening (26) and as a result, national CRC-screening programs with guaiac-based FOBT have started around Europe, *e.g.* United Kingdom, Finland, France, Italy and shortly in Denmark and Norway. In Sweden, only the counties of Stockholm/Gotland have an organized colorectal cancer screening program of the 60-69 years old general population using guaiac-based FOBT as the primary screening test.

1.5 A call for a population-based Swedish CRC-screening study

The Swedish minister of Health and Social affairs, Göran Hägglund, funded a task force in 2011 in order to design a study of the effectiveness of colorectal cancer screening of the average risk population of Sweden. The Swedish Association of Local Authorities and Regions (SALAR) organized the task force in cooperation with the newly started Regional Cancer Centers of Sweden. A committee was formed with one committee member from each national center and professor Rolf Hultcrantz, the Principal Investigator of the present study, has been the chairman of the committee. The present study was designed based on what data was needed to get better outcome of colorectal cancer screening. The study design was submitted in January 2012 to the Ministry of Health and Social affairs and, since then, a thorough work has been carried out in order to get all counties in Sweden to fund the study. Eighteen counties in Sweden achieved the funding in March 2013 covering the actual screening procedures. The counties of Stockholm and Gotland cannot take part in the study due to the already implemented CRC-screening program with Hemoccult®.

2. Study aims

2.1 Primary endpoints

1. To demonstrate if colorectal cancer screening has an effect on the mortality from colorectal cancer in the Swedish population.

2. To demonstrate if colorectal cancer screening has an effect on the incidence of colorectal cancer in the Swedish population.

3. To demonstrate what method should be used in Sweden regarding the effect according to 1 and 2.

2.2 Secondary endpoints

1. To study compliance with the screening program (read study) and what factors are of importance for the adherence rate.

2. To study health economy and costs for colorectal cancer screening.

3. To study the emotional impact of screening on participants and non-participants including eventual change in lifestyle after invitation and/or participation.

4. To study quality control aspects and side effects of screening with colonoscopy.

5. To study pathology by means of quality registries and digital pathology.

6. To study surveillance strategies for adenomas found at colonoscopy.

7. To study associations of DNA in blood with findings at colonoscopy.

8. To study the flora of fecal bacteria among participants and outcome of FIT and colonoscopy.

3. Material and Methods

3.1 Study population

In total 278,280 individuals, residents of Sweden will be randomized from the population register maintained by Swedish tax agency (Skatteverket) (27). The randomized individuals will turn 60-years old the calendar year of randomization. 31,140 individuals will be invited to a primary screening colonoscopy, 60,300 individuals will be invited to high-sensitive FIT and, if positive, to a subsequent colonoscopy and 186,840 persons will serve as controls (11. Figure 1 and 2). The inclusion period is set to three years with a repeated test after two years in the FIT arm.

3.1.1 Inclusion criteria

All individuals 60 years old and living in Sweden and randomized and identified through the Register of the total population.

3.1.2 Exclusion criteria

Residents of the counties of Stockholm and Gotland, individuals with a diagnose of colorectal cancer and/or anal cancer in the continuous updated cancer registers run by the local Regional Cancer Centers, individuals randomized to be included in the ongoing NordICC-trial (28) and individuals living in Västernorrland County – the only county (region) in Sweden except for Stockholm/Gotland not participating in the study.

3.1.3 Randomization

From the Swedish tax agency three sets of individuals by random from the population register (27) will be asked for, approximately 67 000 individuals each born in 1954, 1955 and 1956. The randomization process will then be performed at the Head secretariat in the beginning of 2014, 2015 and 2016 and in the colonoscopy arm 2017, 2018):

- 6,700 individuals born 1954, 1955, 1956 will randomized, by county (region) in proportion to the population, to be invited to primary colonoscopy (3.2.1 Colonoscopy) and 5,250 born in 1957 and1958.

- Three individuals per primary colonoscopy individual will be matched according to year of birth, gender and county (region) of residence and invited to FIT (3.2.2 FIT).
- Six individuals per primary colonoscopy individual will be matched according to year of birth, gender and county (region) of residence and serve as controls.

3.2 Invitation procedures

3.2.1 *Colonoscopy arm*

All individuals randomized to the colonoscopy arm will receive an invitational letter by regular mail including the brochure with information about the incentives of the study. The invitee will be informed that they shortly by mail will receive an appointment for colonoscopy within 8 weeks, sent from the endoscopy clinic in their area of residency.

3.2.2 FIT arm

All individuals randomized to FIT as primary screening test will by regular mail receive an invitation to participate in the study. The invitation includes a brochure with information about the incentives of the study and the need for a follow-up colonoscopy in case of a positive test. The invitation contains a FIT-kit for two separate test samples and instructions on how to take the test. With the invitation is a pre-paid return-envelope for submitting the two tests together directly to the analyzing laboratory. The returned FIT will be stored in Bio bank after analysis.

3.2.3 Controls

The individuals randomized to the control arm will not be contacted and informed about participation as controls in the study. If any individuals would contact the Head secretariat to ask about if they are controls, information will be given.

3.2.4 Undelivered invitations

All invitations will be sent by the Regional Cancer Center, Uppsala/Örebro, where the Head secretariat of SCREESCO is located. Undelivered and returned invitations will be sent out a second time. Since allocation to intervention is by random, we believe the number of undelivered invitations will be approximately the same in both intervention arms.

3.3 Interventions

3.3.1 *Colonoscopy arm*

A primary screening colonoscopy will be carried out once and with an estimated adherence of about 35%, approximately 10,000 colonoscopies will be carried out. The examination will be performed with or without sedation following a standard bowel cleaning preparation. The endoscopy centers will be accredited and the quality of the examiners will be investigated. All details will follow the European guidelines (26). If the colonoscopy is not complete the participant is offered to be examined again with colonoscopy or CT colonoscopy.

3.3.2. *Primary colonoscopy appointment*

With the colonoscopy appointment (within 8 weeks) sent after the initial invitation letter are instructions to call and confirm the appointment, following the same procedure as the participants with a positive FIT (3.3.9 Bowel preparation for follow-up colonoscopy). A difference, though, is that in the group randomized to primary colonoscopy screening, a reminder will be sent out from the CIS-S if no confirmation of the colonoscopy appointment has been performed within 8 weeks. The endoscopy site will then send a new colonoscopy appointment (within 8 weeks) to be confirmed as previously described.

3.3.3 FIT arm and the positivity rate

A high-sensitive FIT (OC-Sensor®) with about 10% positivity rate will be used. The screening method of FIT is well known and established with an adjustable positivity rate from about 2% up to almost 10% (with and opposed effect on specificity). Almost all individuals have traces of blood and hereby hemoglobin in their feces, why the level of sensitivity of the test is an important balance of not missing any lesions, but at the same time minimizing the total number of false positive tests. In SCREESCO people are regarded as having a positive test result if at least one out of the two test samples are positive, i.e. above the set cut off level of 50 µg Hb/L (50 ng Hb/mL). With the used OC-Sensor® 1 µg Hb/L buffer = 0,2 µg Hb/g feces, *i.e.* 50 µg Hb/L buffer equals 10 µg Hb/g feces.

3.3.4 *Two rounds of FIT*

We plan two rounds of FIT – one the first year and one the third year. All individuals randomized to the FIT-arm will have new test-kits sent home after two years, regardless of compliance with the invitation to the first round. Previous studies have been performed with FOBT every second year for ten years and we will follow the findings and perform interim analyses and suggest that further rounds of FIT are carried out if the scientific committee deems it necessary. Two tests per round will be asked for without dietary restrictions. If 50% of the invitees comply and send their FIT test to the laboratory (experiences from the ongoing screening program in the counties of Stockholm and Gotland) and 10% are positive, this will generate approximately 1,200 colonoscopies for each year in the FIT-arm except for year four when the number of generated colonoscopies is estimated to 2,400 due to the second round re-testing.

3.3.5 *Information of FIT-result*

All participating individuals in the FIT-arm will be informed about their test result by mail within 4 weeks after the test was sent in. People with a negative test result will in the same mail be informed that a new test-kit will be sent after two years. People with positive test will be informed by mail that they within one week will get a colonoscopy appointment (within 4 weeks instead of 8 weeks as in primary endoscopy arm [3.3.1] due to positive test) by mail sent by the endoscopy site in their area of residency.

3.3.6 *Reminder and default returned FIT*

People with no tests sent in will get a reminder by mail after 8 weeks and with instructions to call to receive new test kits if they are missing. People with default FITs sent in will receive new test kits with instructions.

3.3.7 *FIT returned after 6 months*

FIT returned after 6 months will be scientifically handled as non-compliers. The test results are expected un-valid due to expiring date of the test.

3.3.8 *Follow-up colonoscopy appointment of individuals with positive FIT*

The endoscopy site in the participants geographical area of residency will electronically within the Central IT-Support System – Study (CIS-S) (6. Data management) simultaneously receive the information about the participant who needs an appointment for colonoscopy and book one.

3.3.9 *Bowel preparation for colonoscopy*

Instructions to call and confirm the colonoscopy appointment within the 4 weeks is included with the mailed colonoscopy appointment and after confirmation the participant with a positive FIT will receive bowel preparation (Laxabon®) by mail without charge. Split dose is highly recommended (26).

3.3.10 *Medical history taken at telephone confirmation of appointment*

At confirmation of the colonoscopy appointment the study nurse will ask a few specific questions about the health status of the invitee, including medications (*e.g.* Warfarin®) and check with the responsible physician in doubt of colonoscopy risks. The invitee will also at the telephone conversation be able to ask questions about the colonoscopy.

3.3.11 *Reminder of follow-up colonoscopy*

Without confirmation by telephone a reminder together with a new colonoscopy appointment time is sent out from the corresponding endoscopy site, following the same confirmation procedure as above.

3.3.12 *Follow-up after positive FIT follow-up colonoscopy*

Individuals with no pathological finding at colonoscopy will be invited to a second FIT after two years and individuals with a pathological finding will be followed-up regarding to the specific clinical guidelines.

3.3.13 *Positive FIT and asking for a new FIT replacing colonoscopy*

Individuals asking for a new FIT instead of a follow-up colonoscopy after a positive test will not have this option. In the study, all positive FIT will be followed-up by a colonoscopy. All individuals invited to the FIT-arm will have a second round of FIT sent home after two years (except those who had a cancer diagnosis or polyp findings requiring surveillance)

3.3.14 *Weight and height measurement and nurse questionnaire*

At both primary colonoscopy and follow-up colonoscopy after a positive FIT, the nurse will weigh and measure height (not only asked for) and ask up to ten health questions regarding *e.g.* smoking, alcohol and use of non-steroid anti-inflammatory drugs to be registered in the CIS-S. The questions will be asked *before* the colonoscopy to limit re-call bias.

3.3.15 *Blood sample at colonoscopy*

At colonoscopy the participant will be asked to leave two blood samples (EDTA®) to be stored in Biobank for future analyses after signing an informed consent (3.4 Informed consent). The individual can decline leaving blood samples for the study, but participate with colonoscopy.

3.3.16 *Positive finding at colonoscopy*

Individuals with pathological finding at colonoscopy, *i.e.* colorectal cancer or advanced adenomas qualifying to the adenoma surveillance program, will be followed-up regarding to the specific local clinical guidelines. No further screening test will be offered within the study for this category of patients. Patients with *other* findings at colonoscopy, *e.g.* inflammatory bowel disease, will be taken care of by the performing endoscopist, but not excluded from a second round of FIT-screening within the SCREESCO-study if randomized to the FIT intervention arm.

3.3.17 *Individuals with positive FIT but negative colonoscopy*

Individuals with a positive FIT but with a negative colonoscopy (no adenoma or colorectal cancer) will not be investigated further within the study, accept for a second round of FIT after two years if randomized to the FIT intervention arm.

3.3.18 *Questionnaire after the colonoscopy*

After the colonoscopy, the participant will receive a short questionnaire about their experiences of the invitation to screening, bowel preparation and colonoscopy examination, together with a pre-paid return envelope addressed to the endoscopy unit. The participant will be asked to fill out and post the questionnaire within 24 hours.

3.4 Informed consent

The SCREESCO-study has approval from the Ethical Review Board (No. 2012/2058-31/3) Stockholm, Sweden, that a returned FIT-test is to be regarded as informed consent to participate in the study. At colonoscopy, an informed consent is signed by both the study participant in the primary intervention arm, the individuals with a positive FIT and the endoscopist informing about the procedure. The informed consents will be stored at the endoscopy site.

3.5 Quality assurance of colonoscopy

A specific part of the study will be aimed at controlling the outcome of the estimated 17 000 colonoscopies. The patients will be subjected to colonoscopy in 33 different centers throughout Sweden in the 18 participating regions (counties). Previous work in this field has demonstrated that a good quality endoscopist should perform more than 100 procedures/year and be able to detect adenomas in more than 20 % of the examinations. Moreover, following intubation of the instrument to the caecum, the withdrawal time should be more than six minutes.

There will be a thorough evaluation of each endoscopist from a quality aspect according to set guidelines. This will be carried out by using a specific quality register in the CIS-S, where data on the success rate of the colonoscopist, findings and side effects will be entered from all the estimated 17 000 colonoscopies. In the study registry, adverse advents such as pain, bleeding and perforations will also be collected. The register data will continuously be cross-linked with data from the National Patient register (29) and the Swedish Causes of Death register (30) in order to find severe adverse events.

The colonoscopy performance register will contain a unique set of data, which could be used for both colonoscopy development as well as the follow-up of the safety and success in a screening setting – enabling correlation of the performance regarding findings and adverse events of each endoscopist and the previous described quality indicators. The results will be stratified by different categories of endoscopists, those with high and medium numbers of procedures annually, with special focus on nurses performing endoscopies since involving nurses in the endoscopies may be an important step and a key to a successful CRC-screening program in the country.

An internet-based on-line system to connect one endoscopist with a set of experts will be developed within the study in order to deliver immediate second opinion of findings during a procedure. Criteria for the identification of adenomatous polyps as opposed to hyperplastic polyps (31) during the endoscopy will also be developed in the study. Hyperplastic polyps are unlikely to develop dysplasia and, hence, do not have to be removed. If they can be identified in the endoscope much work is spared and patient safety is improved.

Moreover, both primary screening colonoscopies and follow-up colonoscopies after a positive FIT test will be evaluated with a participant questionnaire (3.3.18 Questionnaire after the colonoscopy).

3.6 Pathology

All removed adenomatous polyps will be sent for histopathology and stored in biobank for further analyses. Approximately 11 000 adenomas will be removed during the study. A new technique for digitalization of sections from adenomas larger than 10 mm will be developed. Digital images will be made in Aperio machines from sections of adenomas and stored. These images will be used for quality studies in order to develop processes for diagnostic procedures in pathology in colorectal screening. Secondary studies on correlation of evaluation between various pathologists will be performed in order to create kappa-values in collaboration with Swedish gastrointestinal pathologists in the KVAST (Kvalitets- och standardiseringskommittén [in Swedish]) Study Group of the Swedish Society for Pathology (32). The data will be used in order to demonstrate which type of adenomas need to be extra carefully resected and which of them are especially at risk to develop new adenomas. The purpose is to combine these results with the results from the colonoscopy investigation in order to possibly reduce the number of polyps needed to be resected generating a decreased risk for perforation of the colonic wall by the procedure and also a reduced work-load for both endoscopists and pathologists (31).

3.7 Secondary studies

3.7.1 Complier and non-complier experiences

3.7.2 Health-economy

3.7.3 Quality assurance

3.7.4 Surveillance of adenomas

3.7.5 DNA in blood

3.7.6 Bacteria in feces

3.7.7 Non-steroid anti-inflammatory drugs and adenoma development

3.8 Follow-up

Follow-up time is set at 15 years after inclusion and invitation for the primary endpoint colorectal cancer mortality and incidence with interim analysis at 5 and 10 years. Data of all 278,280 individuals randomized from the population register (FIT-, primary colonoscopy- or control arm) will be obtained from the Causes of Death register (30) and the Cancer register (1) managed by the Swedish National Board of Health and Welfare. Regarding secondary outcomes, *e.g.* quality control of performed colonoscopies and non-steroid anti-inflammatory drugs and adenoma development, information will be retrieved from registries such as the Patient register (29) and the Drug register (33).

4. Ethical considerations

To randomize people from the population register and invite them to screening colonoscopy (or not when control) is an ethical challenge. Primarily, we do have to consider possible risks for the participants*, i.e.* side effects of the primary and follow-up colonoscopies, but secondarily, we also have to consider the stress a false positive test could generate. Furthermore, we will cross link register information of individuals randomized as controls without their informed consent and the information generated must be treated rigorously and that is why the controls are de-identified and the register information gathered aggregated at group level. On a population level, it is of utter most importance that the study is performed. Most certainly, due to the increasing frequency of opportunistic screening, we only have one chance to get a solid answer to our primary endpoint - to demonstrate if colorectal cancer screening has an effect on the mortality from colorectal cancer in the Swedish population. The study has been processed and approved by the regional Ethics Review Board at Karolinska Institutet, Stockholm, Sweden (No. 2012/2058-31/3).

5. Statistical analyses

All individuals will be randomized and allocated to one of three arms; colonoscopy, FIT or control. Disease specific mortality is the variable used for power analysis. Individuals registered in the national Register of the total population will be the bases for the intention to screen analysis and the follow up period will be 15 years.

The lifetime cumulative mortality in colorectal cancer in Sweden is about 1% after 15 years. With a 80% power and a 2.5% significance level according to the Bonferroni method the present study need to randomize 20,100 persons in the colonoscopy arm, 60,186 in the FIT arm and 120,372 in the control arm.

Based on previous studies, we estimate that the reduction in mortality will be about 30% for those examined with FIT and a subsequent colonoscopy if the FIT is positive and approximately 50% for individuals who are examined with a primary screening colonoscopy. The compliance is estimated to about 50% in the FIT-arm and approximately 10% of them will have a positive test and invited to follow-up colonoscopy with 80-90% adherence rate. Compliance with primary colonoscopy is estimated to 50% and there is supposed to be a very low contamination from opportunistic screening. **In 2016 we prolonged the colonoscopy arm due to a lower than expected compliance, 35% instead of 50% and added 10, 500 participants.**

6. Data management

The Central IT-Support System – Study (CIS-S) platform will be located at the Head secretariat. All information generated by the invitation routines, laboratory tests and findings at colonoscopy, as well as individual questionnaire information, will be automatically registered in the system prospectively and available with explicit restriction to guarantee discretion of personal information of individuals.

7. Head secretariat

The Head secretariat of the study is situated at the Regional Cancer Centre in the Uppsala/Örebro region, Uppsala, Sweden.

http://www.cancercentrum.se/sv/uppsalaorebro/Aktuellt/Nyheter/tarmcancerscreeningstudie-startar-forsta-kvartalet-2014/

http://www.cancercentrum.se/sv/uppsalaorebro/Funktioner/in-english/

8. Participating centers

There are 33 participating endoscopy sites distributed nationally and in the areas of residency of the invitees (and controls) of the study.

9. Main study publications

Descriptive paper of the screening study (methods and rationale).

Compliance, findings and negative effects of the screening initiative.

Emotional impact of screening on participants and non-participants.

Quality assurance of screening colonoscopy and pathology reporting.

Health economy of colorectal cancer screening implementation.

Follow-up analyses regarding primary end-point at 10 and 15 years.

Secondary studies (3.7 Secondary studies).

10. Scientific committee

Name	Country	Area of expertise
Rolf Hultcrantz	Sweden	Gastroenterology
Lars Holmberg	Sweden	Screening, surgery
Anders Ekbom	Sweden	Epidemiology
Anna Forsberg	Sweden	Gastroenterology
Robert Steele	United Kingdom	Surgery
Richard Palmqvist	Sweden	Pathology
Mef Nilbert	Sweden	Molecular oncology
Andreas Pischel	Sweden	Endoscopy
Marc Bruse	Belgium	Biostatistics
Yvonne Wengström	Sweden	Qualitative research
Per Carlsson	Sweden	Health economy
Lars Engstrand	Sweden	Microbiota
Johannes Blom	Sweden	Screening, surgery

Addendum May 06 2021

Updated list of members Scientific Committee

**Name**	**Country**	**Area of expertise**
Rolf Hultcrantz	Sweden	Gastroenterology
Lars Holmberg	Sweden	Screening, surgery
Anders Ekbom	Sweden	Epidemiology, Surgery
Anna Forsberg	Sweden	Gastroenterology
Robert Steele	United Kingdom	Screening, surgery
Chris Metcalfe	United Kingdom	Medical Statistics
Christian Löwbeer	Sweden	Laboratory medicine, Clinical chemistry
Andreas Pischel	Sweden	Endoscopy
Yvonne Wengström	Sweden	Qualitative research
Lars-Åke Levin	Sweden	Health economy
Lars Engstrand	Sweden	Microbiota
Johannes Blom	Sweden	Screening, Surgery
Mikael Hellström	Sweden	Radiology
Kaisa Fritzell	Sweden	Qualitative research
Ulf Strömberg	Sweden	Mathematical statistics

11. Figures

11.1 Figure 2. Flow chart of invitation procedure of individuals randomized to intervention FIT

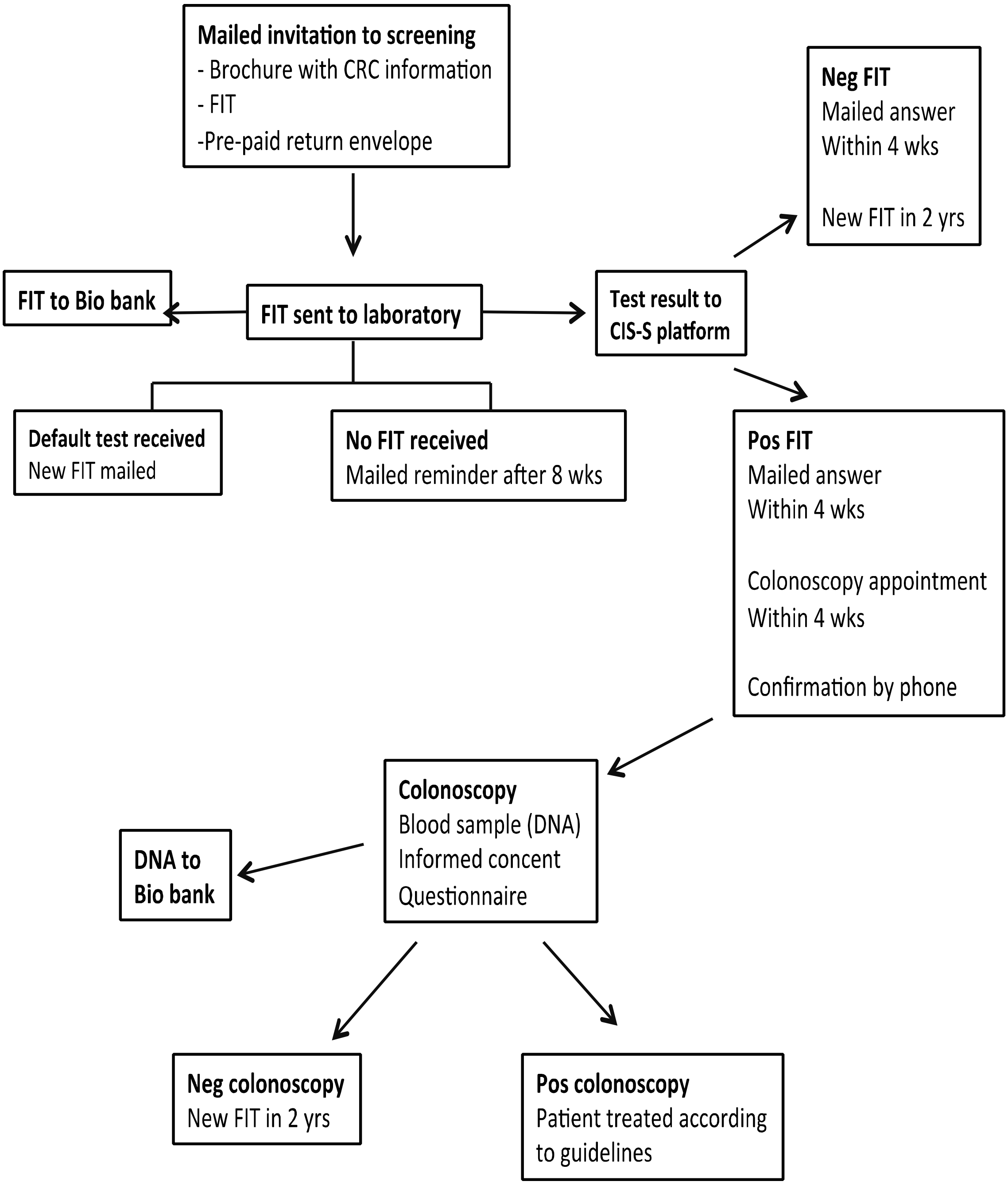

11.2 Figure 1. Flow chart of invitation procedure of individuals randomized to intervention primary colonoscopy

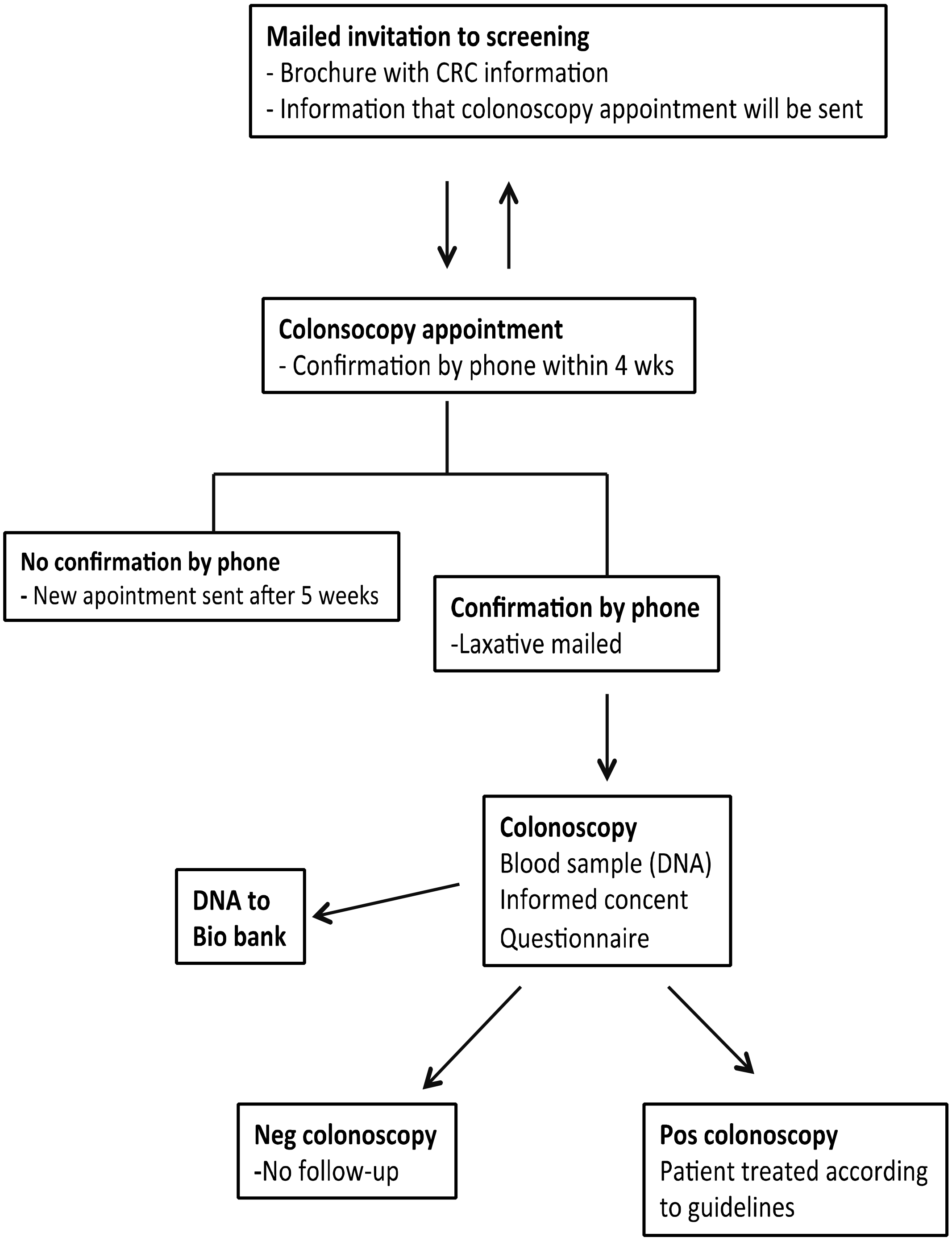

12. References

1. Cancer register http://www.socialstyrelsen.se/register/halsodataregister/cancerregistret/inenglish: National Board of Health and Welfare; [
2. Muto T, Bussey HJ, Morson BC. The evolution of cancer of the colon and rectum. Cancer. 1975;36(6):2251-70.
3. Vogelstein B, Fearon ER, Hamilton SR, Kern SE, Preisinger AC, Leppert M, et al. Genetic alterations during colorectal-tumor development. N Engl J Med. 1988;319(9):525-32.
4. Winawer SJ, Zauber AG, Ho MN, O'Brien MJ, Gottlieb LS, Sternberg SS, et al. Prevention of colorectal cancer by colonoscopic polypectomy. The National Polyp Study Workgroup. N Engl J Med. 1993;329(27):1977-81.
5. Muller AD, Sonnenberg A. Prevention of colorectal cancer by flexible endoscopy and polypectomy. A case-control study of 32,702 veterans. Ann Intern Med. 1995;123(12):904-10.
6. Thiis-Evensen E, Hoff GS, Sauar J, Langmark F, Majak BM, Vatn MH. Population-based surveillance by colonoscopy: effect on the incidence of colorectal cancer. Telemark Polyp Study I. Scand J Gastroenterol. 1999;34(4):414-20.
7. Armitage N. Screening for cancer. In: Morris D, Kearsley, J, Williams, C, editor. Cancer: a comprehensive clnical guide. pp 9-20. U.K.: Taylor and Francis Ltd 1997.
8. Mandel JS, Bond JH, Church TR, Snover DC, Bradley GM, Schuman LM, et al. Reducing mortality from colorectal cancer by screening for fecal occult blood. Minnesota Colon Cancer Control Study. N Engl J Med. 1993;328(19):1365-71.
9. Kronborg O, Fenger C, Olsen J, Jorgensen OD, Sondergaard O. Randomised study of screening for colorectal cancer with faecal-occult-blood test. Lancet. 1996;348(9040):1467-71.
10. Hardcastle JD, Chamberlain JO, Robinson MH, Moss SM, Amar SS, Balfour TW, et al. Randomised controlled trial of faecal-occult-blood screening for colorectal cancer. Lancet. 1996;348(9040):1472-7.
11. Lindholm E, Brevinge H, Haglind E. Survival benefit in a randomized clinical trial of faecal occult blood screening for colorectal cancer. Br J Surg. 2008;95(8):1029-36.
12. Atkin WS, Edwards R, Kralj-Hans I, Wooldrage K, Hart AR, Northover JM, et al. Once-only flexible sigmoidoscopy screening in prevention of colorectal cancer: a multicentre randomised controlled trial. Lancet. 2010;375(9726):1624-33.
13. Segnan N, Armaroli P, Bonelli L, Risio M, Sciallero S, Zappa M, et al. Once-only sigmoidoscopy in colorectal cancer screening: follow-up findings of the Italian Randomized Controlled Trial--SCORE. J Natl Cancer Inst. 2011;103(17):1310-22.
14. Schoen RE, Pinsky PF, Weissfeld JL, Yokochi LA, Church T, Laiyemo AO, et al. Colorectal-cancer incidence and mortality with screening flexible sigmoidoscopy. N Engl J Med. 2012;366(25):2345-57.
15. Faivre J, Bouvier AM, Bonithon-Kopp C. Epidemiology and screening of colorectal cancer. Best Pract Res Clin Gastroenterol. 2002;16(2):187-99.
16. Richardson A. Screening and the number needed to treat. J Med Screen. 2001;8(3):125-7.

17. Bretthauer M. Colorectal cancer screening. J Intern Med. 2011;270(2):87-98.
18. Brouwers MC, De Vito C, Bahirathan L, Carol A, Carroll JC, Cotterchio M, et al. What implementation interventions increase cancer screening rates? a systematic review. Implementation science : IS. 2011;6:111.
19. Tinmouth J, Ritvo P, McGregor SE, Claus D, Pasut G, Myers RE, et al. A qualitative evaluation of strategies to increase colorectal cancer screening uptake. Can Fam Physician. 2011;57(1):e7-15.
20. Blom J, Liden A, Jeppsson B, Holmberg L, Pahlman L. Compliance and findings in a Swedish population screened for colorectal cancer with sigmoidoscopy. Eur J Surg Oncol. 2002;28(8):827-31.
21. Blom J, Liden A, Nilsson J, Pahlman L, Nyren O, Holmberg L. Colorectal cancer screening with flexible sigmoidoscopy-participants' experiences and technical feasibility. Eur J Surg Oncol. 2004;30(4):362-9.
22. Collins RE, Lopez LM, Marteau TM. Emotional impact of screening: a systematic review and meta-analysis. BMC Public Health. 2011;11:603.
23. Tornberg S, Lundstrom V, Gustafsson S, Hultkrantz R. [The first year with colorectal cancer screening in Stockholm. Careful monitoring and quality control of the whole process is necessary]. Lakartidningen. 2010;107(26-28):1709-11.
24. Foss C, Ellefsen B. The value of combining qualitative and quantitative approaches in nursing research by means of method triangulation. J Adv Nurs. 2002;40(2):242-8.
25. Ostlund U, Kidd L, Wengstrom Y, Rowa-Dewar N. Combining qualitative and quantitative research within mixed method research designs: a methodological review. Int J Nurs Stud. 2011;48(3):369-83.
26. von Karsa L, Patnick J, Segnan N. European guidelines for quality assurance in colorectal cancer screening and diagnosis. First Edition--Executive summary. Endoscopy. 2012;44 Suppl 3:SE1-8.
27. Register of the total population https://www.skatteverket.se/download/18.2b543913a42158acf80003761/1386594730871/AllmänBeskrivning.pdf: Swedish tax agency; [
28. Kaminski MF, Bretthauer M, Zauber AG, Kuipers EJ, Adami HO, van Ballegooijen M, et al. The NordICC Study: rationale and design of a randomized trial on colonoscopy screening for colorectal cancer. Endoscopy. 2012;44(7):695-702.
29. Patient register http://www.socialstyrelsen.se/register/halsodataregister/patientregistret/inenglish: National Board of Health and Welfare; [
30. Causes of Death register http://www.socialstyrelsen.se/register/dodsorsaksregistret: National Board of Health and Welfare; [
31. Wallace MB, Kiesslich R. Advances in endoscopic imaging of colorectal neoplasia. Gastroenterology. 2010;138(6):2140-50.
32. Hamilton SR AL. Pathology and Genetics. Tumours of the Digestive System, WHO Classification of tumours, vol. 2. IARC WHO, Classification of tumours, No 2; 20.
33. Drug register http://www.socialstyrelsen.se/register/halsodataregister/lakemedelsregistret National Board of Health and Welfare; [cited 2013 December].

SCREESCO – Screening of Swedish Colons

Study Protocol Version 3.0
2025-01-16

Principal investigator of SCREESCO and endoscopist: Dr Anna Forsberg, Division of Clinical Epidemiology, Department of Medicine K2, Solna, Karolinska Institutet, Stockholm, Sweden. Email: anna.forsberg@ki.se

Chair of the SCREESCO scientific committee and epidemiologist: Dr Jonas F. Ludvigsson, Department of Medical Epidemiology and Biostatistics, Karolinska Institutet, Stockholm, Sweden, and the Department of Pediatrics, Örebro University Hospital, Örebro, Sweden.

Medical statistician: Chris Metcalfe, Bristol Medical School: Population Health Sciences, University of Bristol, United Kingdom.

Epidemiologist: Ulf Strömberg, School of Public Health and Community Medicine, Institute of Medicine, Sahlgrenska Academy at University of Gothenburg.

Research group leader of Cancer prevention and screening, Karolinska Institutet: Johannes Blom, Department of Surgery, Södersjukhuset, and Department of Clinical Science and Education, Karolinska Institutet, Stockholm, Sweden.

Expertise in microbiome analyses, logistics, storage, DNA extraction and metagenome sequencing: Lars Engstrand, Department of Microbiology and Department of Tumor and Cell Biology.

Expertise in qualitative research: Kaisa Fritzell, Department of Neurobiology and Department of Care Sciences and Society.

Radiology specialist in abdominal imaging, expertise in evaluation of diagnostic tests: Mikael Hellström, Department of Radiology, Institute of Clinical Sciences at Sahlgrenska Academy, Gothenburg University, Gothenburg, Sweden.

Specialist in Clinical Chemistry and Laboratory Medicine: Christian Löwbeer, Department of Laboratory Medicine, Division of Clinical Chemistry, Karolinska Institutet, Stockholm, Sweden, and Department of Clinical Chemistry, SYNLAB Sverige, Täby, Sweden.

Senior scientific advisor, expertise in screening and surgery: Robert Steele, Department of Surgery, Population Health and Genomics, School of Medicine, University of Dundee, Ninewells Hospital, Dundee DD1 9SY, United Kingdom.

Senior scientific advisor, expertise in screening and surgery: Lars Holmberg, Department of Surgical Sciences, Uppsala University, Uppsala, Sweden, and

Translational Oncology & Urology Research (TOUR), School of Cancer and Pharmaceutical Sciences, King's College London, London, UK.

Principal Statistician and Data Manager of SCREESCO: Marcus Westerberg, Department of Surgical Sciences, Uppsala University, Uppsala, Sweden.

Summary

Colorectal cancer (CRC) is a major cause of death in Sweden. There are approximately 6000 new cases each year in Sweden and the disease specific mortality is more than 40%. The risk is about 1% to develop CRC between 60-70 years of age, making 60-year-olds a suitable target population for colorectal cancer screening.

The Swedish Ministry of Health and Social affairs has proposed a national study on the efficiency of colorectal cancer screening in the Swedish population regarding mortality, but also what screening method to be used. Thirteen participating counties of Sweden now fund the study to be launched in 2014.

Individuals 60 years of age will be randomized from the population register and invited to screening by mail. 31,140 individuals will be invited to primary colonoscopy and 60,300 individuals will be invited to high-sensitive FIT (OC Sensor®) with approximately 10% positivity rate and, if positive, to a subsequent follow-up colonoscopy. When test negative a second round of FIT will be asked for in two years. In total 186,840 randomized individuals will not be invited to screening serve as controls and will be followed in the Swedish Cancer Register. The inclusion period is set to five years (five years including the second round of FIT) generating approximately 17,000 colonoscopies at a compliance rate of 35% in the colonoscopy arm and 50% in the FIT arm.

Follow-up time is set to 15 years with the primary endpoint disease specific mortality and colorectal cancer incidence. Secondary outcomes, by others, to be studied are in short quality assurance variables of colonoscopy, participants and non-participants experiences of the invitation and the screening procedure, health economy measures of the CRC-screening study and when implemented in clinical care.

Table of contents

1. Background ... 6
1.1 Colorectal cancer and screening ... 6
1.2 Evaluation of a screening program ... 6
1.2.1 Effectiveness ... 6
1.2.2 The screening test ... 7
1.2.3 Compliance ... 8
1.2.4 Cost-effectiveness ... 8
1.3 Challenges in colorectal cancer screening ... 9
1.3.1 Colonoscopy quality assurance ... 9
1.3.2 Lack of pathologists ... 9
1.3.3 Compliance and emotional effects ... 9
1.4 Colorectal cancer screening in Sweden ... 9
1.5 A call for a population-based Swedish CRC-screening study ... 10
2. Study aims ... 10
2.1 Primary endpoints ... 10
2.2 Secondary endpoints ... 10
3. Material and Methods ... 11
3.1 Study population ... 11
3.1.1 Inclusion criteria ... 11
3.1.2 Exclusion criteria ... 11
3.1.3 Randomization ... 11
3.2 Invitation procedures ... 11
3.2.1 Colonoscopy arm ... 11
3.2.2 FIT arm ... 12
3.2.3 Controls ... 12
3.2.4 Undelivered invitations ... 12
3.3 Interventions ... 12
3.3.1 Colonoscopy arm ... 12
3.3.2. Primary colonoscopy appointment ... 12
3.3.3 FIT arm and the positivity rate ... 12
3.3.4 Two rounds of FIT ... 13
3.3.5 Information of FIT-result ... 13
3.3.6 Reminder and default returned FIT ... 13
3.3.7 FIT returned after 6 months ... 13

3.3.8 Follow-up colonoscopy appointment of individuals with positive FIT 13
3.3.9 Bowel preparation for colonoscopy 14
3.3.10 Medical history taken at telephone confirmation of appointment 14
3.3.11 Reminder of follow-up colonoscopy 14
3.3.12 Follow-up after positive FIT follow-up colonoscopy 14
3.3.13 Positive FIT and asking for a new FIT replacing colonoscopy 14
3.3.14 Weight and height measurement and nurse questionnaire 14
3.3.15 Blood sample at colonoscopy 14
3.3.16 Positive finding at colonoscopy 14
3.3.17 Individuals with positive FIT but negative colonoscopy 15
3.3.18 Questionnaire after the colonoscopy 15
3.4 Informed consent 15
3.5 Quality assurance of colonoscopy 15
3.6 Pathology 16
3.7 Secondary studies 16
3.8 Follow-up 17
4. Ethical considerations 17
5. Statistical analyses 17
6. Data management 18
7. Head secretariat 18
8. Participating centers supplement: sites and local PIs 19
9. Main study publications 20
10. Scientific committee 21
11. Figures 23
11.2 Figure 1 23
11.1 Figure 2 24
12. References 25
13. Summary of changes to the SCREESCO study protocol and statistical analysis plan 2013-2024 28

1. Background

1.1 Colorectal cancer and screening

The primary purpose of cancer screening is to reduce mortality from the disease in the population by finding the cancer at an early and treatable stage. The cancer has to be an important health problem to be a suitable target for screening. With approximately 6 000 new cases each year in Sweden (1), colorectal cancer (CRC) is the third most common cancer in Sweden (after prostate- and breast cancer) and, hence, a relatively common disease, but it is also a major cause of death. There is a 1% risk to develop CRC between the ages 60-75 years, and the five-year survival rate is close to 60%. The prognosis is related to if the cancer is detected at an early or late stage of the disease. When detected at an early stage, there is a 90% five-year survival, as compared to only a 10% five-year survival if the cancer at detected at a late stage.

CRC is usually detected clinically by patient symptoms, either an alteration in bowel habits due to obstruction of the lumen, visual blood in the stool or symptoms due to anemia caused by a bleeding from the tumor. Both larger precursor stages – the adenomatous polyp (2, 3) – and cancers bleed and could be detected by sensitive methods to analyze blood in the stool. All patients diagnosed with colorectal cancer need treatment, but there is a significant difference in suffers and costs depending on the stage the disease at diagnosis. With screening early stages of the disease will be found before they are clinically detected.

Removal of adenomatous polyps (adenomas) has a protective effect against colorectal cancer development (4, 5, 6) and, consequentially, a colorectal cancer screening program also might have the potential of decreasing the future incidence of the disease.

1.2 Evaluation of a screening program

1.2.1 Effectiveness

The effectiveness of a screening program is the ability of the program to reduce the disease specific mortality. Survival is not a valid measure of effectiveness because of the possibilities of bias; selection bias (when screened subjects and non-screened controls represent different populations), lead time bias (earlier diagnosis in screen-detected cancers adds time to the total survival time) and length biased sampling (screen-detected tumors often grow slowly and might be less malignant).

The most valid measure of effectiveness in screening is a lower mortality in the screened group, as compared to the non-screened group evaluated in a randomized controlled trial (RCT):

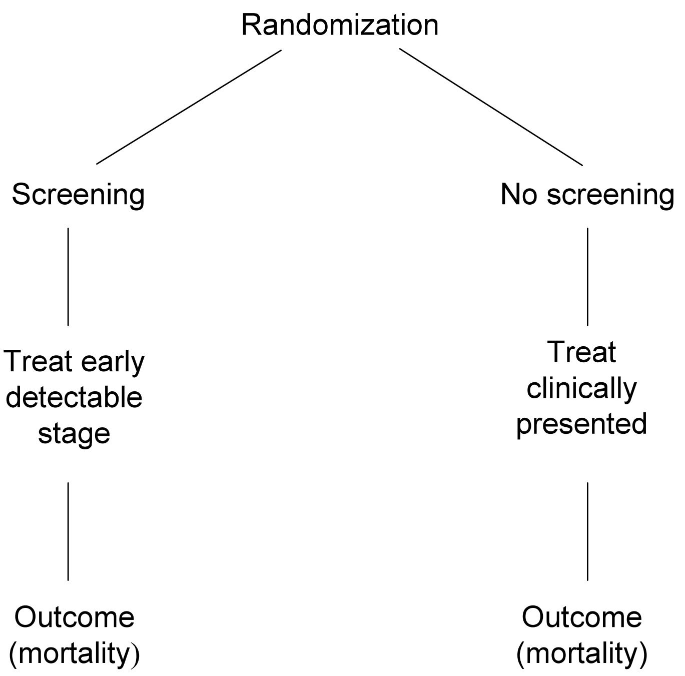

1.2.2 The screening test

A large majority of individuals in the general population using the screening test offered will not have colorectal cancer. Therefore the test must be free from unwanted side effects, inexpensive, but also simple to take and easy to interpret (7). Furthermore, a high sensitivity (to limit the number of missed cancers) and specificity (to limit the number of incorrect diagnosed cancers), is a prerequisite for a screening test to be used in a program of the average risk population.

There are a number of screening tests and methods to examine the colorectum in order to find CRC and/or adenomatous polyps:

<u>Indirect tests:</u> The most commonly used screening test has been the guaiac based fecal occult blood test (FOBT) (Hemoccult®). Four larger RCTs have demonstrated a 16% decreased in CRC mortality with the test in screening (8, 9, 10, 11). The degree of mortality reduction with Hemoccult® depends on the compliance with the test and the dietary restrictions, the sensitivity of the test, the screening frequency (annual or biennial), the number of screening rounds the subjects participate in and, also, the compliance with the diagnostic follow-up colonoscopy after a positive test. More advanced fecal immunological tests (FITs) with higher sensitivity, but only a marginally increase in the false positive rate (specificity), are now available. FIT demonstrates presence of human blood only, as opposed to the guaiac test that also can be positive due to animal hemoglobin. Moreover, no dietary restrictions are needed with FIT.

<u>Direct tests:</u> The main advantages with endoscopy (*e.g.* colonoscopy and sigmoidoscopy) are the direct visualization of the colorectum and the possibility of obtaining tissue samples from suspected cancer lesions for histopathology and/or removing adenomatous polyps during the procedure. The bowel can be examined with sigmoidoscopy and a subsequent colonoscopy in case of

pathological finding within the reach of the sigmoidoscope (approximately 60 cm) or with a complete colonoscopy as primary test (when 1/3 of tumors will appear in the right part of the colon). Recently, three randomized controlled trials with sigmoidoscopy as the primary screening test have demonstrated both a reduced disease specific mortality of around 30%, but also a reduced incidence of CRC of as much as 40% (12, 13, 14).

Up to now, there are no larger randomized controlled studies of the average risk population published demonstrating a disease specific mortality reduction with colonoscopy as the primary screening test.

1.2.3 Compliance

The proportion of individuals offered a screening test who take the test is referred to as compliance (7). The compliance to a screening program is a major determinant of the program's effectiveness and there has to be a rigid organization with a call- recall system and quality assurance in a screening program to be effective (15).

Instead of number needed to treat (NNT) used to estimate the efficacy in interventional RCTs evaluating medication, the number needed to screen (NNS) is used in the evaluation of RCTs of screening. The NNS is the number of individuals who need to be invited (offered) screening to prevent one death (intention-to-screen). The results then reflect the efficacy of the screening program to reduce mortality among those *invited* to screening. With NNS there is often an underestimation of the efficacy of the screening test in those people who actually participate – are being screened. The NNS for most screening programs are usually much higher than the number of people who have to participate to prevent one death (16). It is only the participants that can contribute to the mortality reduction achieved by the screening program and with low compliance the number of deaths prevented will be few, and consequently the NNS will be large (16). Therefore, a high participation rate in a screening program is important to be able to evaluate its effectiveness on mortality.

1.2.4 Cost-effectiveness

There are a large variety of variables involved when measuring the cost-effectiveness of a colorectal cancer screening program and focus cannot only be on the eventual incidence and mortality reduction of the disease. Firstly, one has to make assumptions about the duration of the early, asymptomatic and curable stage of the disease. Secondly, one has to estimate the effectiveness and negative effects of the screening procedure, such as morbidity due to complications and costs. A low compliance in a screening program will both effect the incidence and mortality reduction achieved and the cost-effectiveness of the program.

1.3 Challenges in colorectal cancer screening

1.3.1 Colonoscopy quality assurance

There are some outstanding challenges in implementing colorectal cancer screening for the average risk population. One important issue is the quality assurance of the endoscopic examinations and the follow-up of eventual findings. If the quality of the examinations is not excellent, but with frequent practical mistakes and neglect to find adenomas and or cancers, the positive effect of the screening procedure for that particular individual and of the program as a whole is both diminished and unethical (17, 18, 19, 20, 21).

Colonoscopy resources and quality will be a key issue in the present study. Currently in Sweden, about 65 000 procedures are carried out outside the Stockholm area yearly, while approximately 30 000 are carried out in Stockholm. A crude estimate is that a future national screening program will need a substantial number of colonoscopies, and, thus, much work is needed to both increase the number of colonoscopies performed nationally and also increase and secure the quality of the investigations.

Adenomatous polyps should be removed in order to prevent the occurrence of CRC. The removal of adenomas is a sometimes difficult and dangerous procedure, since it may cause bleeding and perforation of the bowel wall. It is important that the adenoma is completely removed and correctly diagnosed. Thus, both a skillful endoscopists and high-quality histopathology are extremely important.

1.3.2 Lack of pathologists

Another problem when implementing a screening program is the created increased burden for pathologists. There is a need for standardization of the use of the diagnostic criteria and the possibility to have a group of pathologists to evaluate the sections. In order to do so, it would be of great advantage if the histological sections could be stained and digitally processed and evaluated electronically.

1.3.3 Compliance and emotional effects

Another important factor, in need of further studies, is the actual adherence rate or compliance discussed above and the emotional effects of the inclusion of healthy persons from the general population in colorectal cancer screening (22, 23, 24, 25).

1.4 Colorectal cancer screening in Sweden

The National Board of Health and Welfare has not been recommending CRC screening due to lack of experiences of screening in routine health care and refer CRC screening to the Research and Development list waiting for studies outside Sweden to generate results. Based on the results from the four RCTs with a net CRC-mortality reduction of 16% (8, 9, 10, 11) the Council of the European Union in 2003 recommended CRC-screening with guaiac-based fecal occult blood test (FOBT) in the average risk population aged 55-74 years old. The recommendations have recently been updated in the European Guidelines of

Quality Assurance in CRC-screening (26) and as a result, national CRC-screening programs with guaiac-based FOBT have started around Europe, *e.g.* United Kingdom, Finland, France, Italy and shortly in Denmark and Norway. In Sweden, only the counties of Stockholm/Gotland have an organized colorectal cancer screening program of the 60-69 years old general population using guaiac-based FOBT as the primary screening test.

1.5 A call for a population-based Swedish CRC-screening study

The Swedish minister of Health and Social affairs, Göran Hägglund, funded a task force in 2011 in order to design a study of the effectiveness of colorectal cancer screening of the average risk population of Sweden. The Swedish Association of Local Authorities and Regions (SALAR) organized the task force in cooperation with the newly started Regional Cancer Centers of Sweden. A committee was formed with one committee member from each national center and professor Rolf Hultcrantz, the Principal Investigator of the present study, has been the chairman of the committee. The present study was designed based on what data was needed to get better outcome of colorectal cancer screening. The study design was submitted in January 2012 to the Ministry of Health and Social affairs and, since then, a thorough work has been carried out in order to get all counties in Sweden to fund the study. Eighteen counties in Sweden achieved the funding in March 2013 covering the actual screening procedures. The counties of Stockholm and Gotland cannot take part in the study due to the already implemented CRC-screening program with Hemoccult®.

2. Study aims

2.1 Primary endpoints

1. To investigate if colorectal cancer screening has an effect on the mortality from colorectal cancer in the Swedish population.
2. To investigate if colorectal cancer screening has an effect on the incidence of colorectal cancer in the Swedish population.
3. To investigate what method should be used in Sweden regarding the effect according to 1 and 2.

2.2 Secondary endpoints

- To study associations of DNA in blood with findings at colonoscopy

- To study the FIT microbiome profile in CRC patients and controls to identify microbial biomarkers that are associated with colonoscopy findings

3. Material and Methods

3.1 Study population

In total 278,280 individuals, residents of Sweden will be randomized from the population register maintained by Swedish tax agency (Skatteverket) (27). The randomized individuals will turn 60-years old the calendar year of randomization. 31,140 individuals will be invited to a primary screening colonoscopy, 60,300 individuals will be invited to high-sensitive FIT and, if positive, to a subsequent colonoscopy and 186,840 persons will serve as controls (11. Figure 1 and 2). The inclusion period is set to three years with a repeated test after two years in the FIT arm.

3.1.1 Inclusion criteria

All individuals 60 years old and living in Sweden and randomized and identified through the Register of the total population.

3.1.2 Exclusion criteria

Residents of the counties of Stockholm and Gotland, individuals with a diagnosis of colorectal cancer and/or anal cancer in the continuous updated cancer registers run by the local Regional Cancer Centers, individuals randomized to be included in the ongoing NordICC-trial (28) and individuals living in Västernorrland County – the only county (region) in Sweden except for Stockholm/Gotland not participating in the study.

3.1.3 Randomization

From the Swedish tax agency three sets of individuals by random from the population register (27) will be asked for, approximately 67 000 individuals each born in 1954, 1955 and 1956. The randomization process will then be performed at the Head secretariat in the beginning of 2014, 2015 and 2016 and in the colonoscopy arm 2017, 2018):

- 6,700 individuals born 1954, 1955, 1956 will randomized, by county (region) in proportion to the population, to be invited to primary colonoscopy (3.2.1 Colonoscopy) and 5,250 born in 1957 and1958.
- Three individuals per primary colonoscopy individual will be matched according to year of birth, gender and county (region) of residence and invited to FIT (3.2.2 FIT).
- Six individuals per primary colonoscopy individual will be matched according to year of birth, gender and county (region) of residence and serve as controls.

3.2 Invitation procedures

3.2.1 Colonoscopy arm

All individuals randomized to the colonoscopy arm will receive an invitation letter by regular mail including the brochure with information about the incentives of the study. The invitee will be informed that they shortly by mail will receive an appointment for colonoscopy within 8 weeks, sent from the endoscopy clinic in their area of residency.

3.2.2 FIT arm
All individuals randomized to FIT as primary screening test will by regular mail receive an invitation to participate in the study. The invitation includes a brochure with information about the incentives of the study and the need for a follow-up colonoscopy in case of a positive test. The invitation contains a FIT-kit for two separate test samples and instructions on how to take the test. With the invitation is a pre-paid return-envelope for submitting the two tests together directly to the analyzing laboratory. The returned FIT will be stored in Bio bank after analysis.

3.2.3 Controls
The individuals randomized to the control arm will not be contacted and informed about participation as controls in the study. If any individuals would contact the Head secretariat to ask about if they are controls, information will be given.

3.2.4 Undelivered invitations
All invitations will be sent by the Regional Cancer Center, Uppsala/Örebro, where the Head secretariat of SCREESCO is located. Undelivered and returned invitations will be sent out a second time. Since allocation to intervention is by random, we believe the number of undelivered invitations will be approximately the same in both intervention arms.

3.3 Interventions

3.3.1 Colonoscopy arm
A primary screening colonoscopy will be carried out once and with an estimated adherence of about 35%, approximately 10,000 colonoscopies will be carried out. The examination will be performed with or without sedation following a standard bowel cleaning preparation. The endoscopy centers will be accredited and the performance of the examiners will be investigated. All requirements will follow the European guidelines (26). If the colonoscopy is not complete the participant is offered to be examined again with colonoscopy or CT colonoscopy.

3.3.2. Primary colonoscopy appointment
With the colonoscopy appointment (within 8 weeks) sent after the initial invitation letter are instructions to call and confirm the appointment, following the same procedure as the participants with a positive FIT (3.3.9 Bowel preparation for follow-up colonoscopy). A difference, though, is that in the group randomized to primary colonoscopy screening, a reminder will be sent out from the CIS-S if no confirmation of the colonoscopy appointment has been performed within 8 weeks. The endoscopy site will then send a new colonoscopy appointment (within 8 weeks) to be confirmed as previously described.

3.3.3 FIT arm and the positivity rate
A high-sensitive FIT (OC-Sensor®) with about 10% positivity rate will be used. The screening method of FIT is well known and established with an adjustable positivity rate from about 2% up to almost 10% (with an opposed effect on

specificity). Almost all individuals have traces of blood and hereby hemoglobin in their feces, why the level of sensitivity of the test is an important balance of not missing any lesions, but at the same time minimizing the total number of false positive tests. In SCREESCO people are regarded as having a positive test result if at least one out of the two test samples are positive, i.e. above the set cut off level of 50 µg Hb/L (50 ng Hb/mL). With the used OC-Sensor® 1 µg Hb/L buffer = 0,2 µg Hb/g feces, *i.e.* 50 µg Hb/L buffer equals 10 µg Hb/g feces.

3.3.4 Two rounds of FIT
We plan two rounds of FIT – one the first year and one the third year. All individuals randomized to the FIT-arm will have new test-kits sent home after two years, regardless of compliance with the invitation to the first round. Previous studies have been performed with FOBT every second year for ten years and we will follow the findings and perform interim analyses and suggest that further rounds of FIT are carried out if the scientific committee deems it necessary. Two tests per round will be asked for without dietary restrictions. If 50% of the invitees comply and send their FIT test to the laboratory (experiences from the ongoing screening program in the counties of Stockholm and Gotland) and 10% are positive, this will generate approximately 1,200 colonoscopies for each year in the FIT-arm except for year four when the number of generated colonoscopies is estimated to 2,400 due to the second round re-testing.

3.3.5 Information of FIT-result
All participating individuals in the FIT-arm will be informed about their test result by mail within 4 weeks after the test was sent in. People with a negative test result will in the same mail be informed that a new test-kit will be sent after two years. People with positive test will be informed by mail that they within one week will get a colonoscopy appointment (within 4 weeks instead of 8 weeks as in primary endoscopy arm [3.3.1] due to positive test) by mail sent by the endoscopy site in their area of residency.

3.3.6 Reminder and default returned FIT
People with no tests sent in will get a reminder by mail after 8 weeks and with instructions to call to receive new test kits if they are missing. People with default FITs sent in will receive new test kits with instructions.

3.3.7 FIT returned after 6 months
FIT returned after 6 months will be scientifically handled as non-compliers. The test results are expected un-valid due to expiring date of the test.

3.3.8 Follow-up colonoscopy appointment of individuals with positive FIT
The endoscopy site in the participants geographical area of residency will electronically within the Central IT-Support System – Study (CIS-S) (6. Data management) simultaneously receive the information about the participant who needs an appointment for colonoscopy and book one.

3.3.9 Bowel preparation for colonoscopy
Instructions to call and confirm the colonoscopy appointment within the 4 weeks is included with the mailed colonoscopy appointment and after confirmation the participant with a positive FIT will receive bowel preparation (Laxabon®) by mail without charge. Split dose is highly recommended (26).

3.3.10 Medical history taken at telephone confirmation of appointment
At confirmation of the colonoscopy appointment the study nurse will ask a few specific questions about the health status of the invitee, including medications (*e.g.* Warfarin®) and check with the responsible physician in doubt of colonoscopy risks. The invitee will also at the telephone conversation be able to ask questions about the colonoscopy.

3.3.11 Reminder of follow-up colonoscopy
Without confirmation by telephone a reminder together with a new colonoscopy appointment time is sent out from the corresponding endoscopy site, following the same confirmation procedure as above.

3.3.12 Follow-up after positive FIT follow-up colonoscopy
Individuals with no pathological finding at colonoscopy will be invited to a second FIT after two years and individuals with a pathological finding will be followed regarding to the specific clinical guidelines.

3.3.13 Positive FIT and asking for a new FIT replacing colonoscopy
Individuals asking for a new FIT instead of a follow-up colonoscopy after a positive test will not have this option. In the study, all positive FIT will be followed by a colonoscopy. All individuals invited to the FIT-arm will have a second round of FIT sent home after two years (except those who had a cancer diagnosis or polyp findings requiring surveillance)

3.3.14 Weight and height measurement and nurse questionnaire
At both primary colonoscopy and follow-up colonoscopy after a positive FIT, the nurse will weigh and measure height (not only asked for) and ask up to ten health questions regarding *e.g.* smoking, alcohol and use of non-steroid anti-inflammatory drugs to be registered in the CIS-S. The questions will be asked *before* the colonoscopy to limit re-call bias.

3.3.15 Blood sample at colonoscopy
At colonoscopy the participant will be asked to leave two blood samples (EDTA®) to be stored in Biobank for future analyses after signing an informed consent (3.4 Informed consent). The individual can decline leaving blood samples for the study, but participate with colonoscopy.

3.3.16 Positive finding at colonoscopy
Individuals with pathological finding at colonoscopy, *i.e.* colorectal cancer or advanced adenomas qualifying to the adenoma surveillance program, will be

followed regarding to the specific local clinical guidelines. No further screening test will be offered within the study for this category of patients. Patients with *other* findings at colonoscopy, *e.g.* inflammatory bowel disease, will be taken care of by the performing endoscopist, but not excluded from a second round of FIT-screening within the SCREESCO-study if randomized to the FIT intervention arm.

3.3.17 Individuals with positive FIT but negative colonoscopy
Individuals with a positive FIT but with a negative colonoscopy (no adenoma or colorectal cancer) will not be investigated further within the study, except for a second round of FIT after two years if randomized to the FIT intervention arm.

3.3.18 Questionnaire after the colonoscopy
After the colonoscopy, the participant will receive a short questionnaire about their experiences of the invitation to screening, bowel preparation and colonoscopy examination, together with a pre-paid return envelope addressed to the endoscopy unit. The participant will be asked to fill out and post the questionnaire within 24 hours.

3.4 Informed consent

The SCREESCO-study has approval from the Ethical Review Board (No. 2012/2058-31/3) Stockholm, Sweden, that a returned FIT-test is to be regarded as informed consent to participate in the study. At colonoscopy, an informed consent is signed by both the study participant in the primary intervention arm, the individuals with a positive FIT and the endoscopist informing about the procedure. The informed consents will be stored at the endoscopy site.

3.5 Quality assurance of colonoscopy

A specific part of the study will be aimed at studying the outcome of the estimated 17 000 colonoscopies. The patients will be subjected to colonoscopy in 33 different centers throughout Sweden in the 18 participating regions (counties). Previous work in this field has demonstrated that a good quality endoscopist should perform more than 100 procedures/year and be able to detect adenomas in more than 20 % of the examinations. Moreover, following intubation of the instrument to the caecum, the withdrawal time should be more than six minutes.

There will be a thorough evaluation endoscopist performance according to set guidelines. This will be carried out by using a specific quality register in the CIS-S, where data on the success rate of the colonoscopist, findings and side effects will be entered from all the estimated 17 000 colonoscopies. In the study registry, adverse advents such as pain, bleeding and perforations will also be collected. The register data will continuously be cross-linked with data from the National Patient register (29) and the Swedish Causes of Death register (30) in order to find severe adverse events.

The colonoscopy performance register will contain a unique set of data, which could be used for both colonoscopy development as well as the follow-up of the safety and success in a screening setting – enabling correlation of the

performance regarding findings and adverse events of each endoscopist and the previous described quality indicators. The results will be stratified by different categories of endoscopists, those with high and medium numbers of procedures annually, with special focus on nurses performing endoscopies since involving nurses in the endoscopies may be an important step and a key to a successful CRC-screening program in the country.

An internet-based on-line system to connect one endoscopist with a set of experts will be developed within the study to deliver immediate second opinion of findings during a procedure. Criteria for the identification of adenomatous polyps as opposed to hyperplastic polyps (31) during the endoscopy will also be developed in the study. Hyperplastic polyps are unlikely to develop dysplasia and, hence, do not have to be removed. If they can be identified in the endoscope much work is saved and patient safety is improved.

Moreover, both primary screening colonoscopies and follow-up colonoscopies after a positive FIT test will be evaluated with a participant questionnaire (3.3.18 Questionnaire after the colonoscopy).

3.6 Pathology

All removed adenomatous polyps will be sent for histopathology and stored in a biobank (Biobank of the Karolinska Institute) for further analyses. Approximately 11 000 adenomas will be removed during the study. A new technique for digitalization of sections from adenomas larger than 10 mm will be developed. Digital images will be made in Aperio machines from sections of adenomas and stored. These images will be used for quality studies to develop processes for diagnostic procedures in pathology in colorectal screening. Secondary studies on correlation of evaluation between various pathologists will be performed in order to create kappa-values in collaboration with Swedish gastrointestinal pathologists in the KVAST (Kvalitets- och standardiseringskommittén [in Swedish]) Study Group of the Swedish Society for Pathology (32). The data will be used to demonstrate which type of adenomas need to be extra carefully resected and which of them are especially at risk to develop new adenomas. The purpose is to combine these results with the results from the colonoscopy investigation in order to possibly reduce the number of polyps needed to be resected generating a decreased risk for perforation of the colonic wall by the procedure and also a reduced work-load for both endoscopists and pathologists (31).

3.7 Secondary studies

The following secondary studies are planned:

- Complier and non-complier experiences
- Health-economy: intervention arms versus control arms
- Quality assurance of primary and secondary screening tests
- DNA in blood and advanced colorectal neoplasia
- Microbiome in feces

- Socio-economical inequities in screening participation and in screening-detected colorectal findings

3.8 Follow-up

Follow-up time is set at 15 years after inclusion and invitation for the primary endpoint colorectal cancer mortality and incidence. The previous versions of the Study Protocol did not however specify when in calendar time the analysis should be performed. The Scientific Committee assessed in 2024 the impact of choice of date for the end of follow-up on the statistical precision for comparing colorectal cancer specific mortality between each of the two active screening arms and the control arm and decided the last date of follow-up is 2030-12-31 (see the Statistical Analysis Plan 3.0 for details). The Scientific Committee also decided, in contrast to what was initially specified, that no interim analyses will be performed for the primary endpoint.

Data of all 278,280 individuals randomized from the population register (FIT-, primary colonoscopy- or control arm) will be obtained from the Causes of Death register (30) and the Cancer register (1) managed by the Swedish National Board of Health and Welfare. Regarding secondary outcomes, *e.g.* quality control of performed colonoscopies and non-steroid anti-inflammatory drugs and adenoma development, information will be retrieved from registries such as The Swedish National Patient Register (29) and The Swedish Prescribed Drug register (33).

4. Ethical considerations

To randomize people from the Total Population Register and invite them to screening colonoscopy (or not when control) is an ethical challenge. Primarily, we do have to consider possible risks for the participants*, i.e.* side effects of the primary and follow-up colonoscopies, but secondarily, we also have to consider the stress a false positive test could generate. Furthermore, we will cross link register information of individuals randomized as controls without their informed consent and the information generated must be treated rigorously and that is why the controls are de-identified and the register information gathered aggregated at group level. On a population level, it is of utter most importance that the study is performed. Most certainly, due to the increasing frequency of opportunistic screening, we only have one chance to get a solid answer to our primary endpoint - to investigate if colorectal cancer screening has an effect on the mortality from colorectal cancer in the Swedish population. The study has been processed and approved by the regional Ethics Review Board at Karolinska Institutet, Stockholm, Sweden (No. 2012/2058-31/3).

5. Statistical analyses

All individuals will be randomized and allocated to one of three arms; colonoscopy, FIT or control. Disease specific mortality is the variable used for

power analysis. Individuals registered in the national Register of the total population will be the bases for the intention to screen analysis.

Study planning including the sample size target were based on the following power calculation. The lifetime cumulative mortality in colorectal cancer in Sweden is about 1% after 15 years. With a 80% power and a 2.5% significance level according to the Bonferroni method the present study need to randomize 20,100 persons in the colonoscopy arm, 60,186 in the FIT arm and 120,372 in the control arm.

Based on previous studies, we estimate that the reduction in mortality will be about 30% for those examined with FIT and a subsequent colonoscopy if the FIT is positive and approximately 50% for individuals who are examined with a primary screening colonoscopy. The compliance is estimated to about 50% in the FIT-arm and approximately 10% of them will have a positive test and invited to follow-up colonoscopy with 80-90% adherence rate. Compliance with primary colonoscopy is estimated to 50% and there is supposed to be a low contamination from opportunistic screening.

In 2016 we prolonged the colonoscopy arm due to a lower-than-expected compliance, 35% instead of 50% and added 10, 500 participants.

See the Statistical Analysis Plan for further details regarding the power calculations and rationale for setting the last date of follow-up to 2030-12-31.

6. Data management

The Central IT-Support System – Study (CIS-S) platform will be located at the Head secretariat. All information generated by the invitation routines, laboratory tests and findings at colonoscopy, as well as individual questionnaire information, will be automatically registered in the system prospectively and available with explicit restriction to guarantee discretion of personal information of individuals.

7. Head secretariat

The Head secretariat of the study is situated at the Regional Cancer Centre in the Uppsala/Örebro region (now called Mellansverige), Uppsala, Sweden.

https://cancercentrum.se/samverkan/vara-uppdrag/prevention-och-tidig-upptackt/screening-tjock-och-andtarmscancer/screesco-studien/

https://cancercentrum.se/samverkan/regional-cancer-centres/

8. Participating centers supplement: sites and local PIs

There are 33 participating endoscopy sites distributed nationally and in the areas of residency of the invitees (and controls) of the study.

SCREESCO Principal investigators per site

Stefan Spinnell<br>Bengt Sundbaum	Sunderby hospital
Leif-Göran Carlsson	Skellefteå hospital
Lars Almersson	Lycksele hospital
Tomas Koczkas	Östersund hospital
Åke Öberg	University hospital of Umeå
Michael Wagner	Uppsala University hospital
Lech Rademacher	Avesta hospital
Stefan Willmarsson	Karlstad hospital
Lars Strandberg	Falun hospital
Verena Voss<br>Jörn Holm<br>Torbjörn Sakari	Gävle hospital
Laszlo Kosztyu	Hudiksvall hospital
Gunilla von Schoultz<br>Karin Nyborg	Lindesberg hospital<br>Karlskoga hospital
Gunter Häselbarth	Mora hospital
Märit Larsson	Eskilstuna hospital
Daniel Nordström	Nyköping hospital
Ronald Malcher<br>Josip Kujundzic	Västerås hospital
Rikard Svernlöv	University hospital of Linköping
Eva Adauktusson<br>Ahmad Kassem	Värnamo hospital
Mats Persborn<br>Jörgen Tolstrup Rasmussen<br>Bengt Druvefors	Eksjö hospital
Roland Persson	Kalmar hospital
Hjalmar Åselius	Västervik hospital
Otto Űberbacher<br>Cyrus Dyarmand	Varberg hospital
Joakim Holmin<br>Robert Kunz	Halmstad hospital
Dietrich Ahlhausen	Northern Älvsborg County hospital
Anders Lasson	Southern Älvsborg County hospital
Andreas Pischel	Sahlgrenska University hospital Gothenburg
Morteza Shafazand	Sahlgrenska University hospital /East (Eastern hospital) Gothenburg
Birgit Edin<br>Fotios Chalkidis	Falköping hospital
Ervin Toth	University hospital of Malmö
Jörgen Torp	Kristianstad hospital
Matthias Hoeschen	Helsingborg hospital
Björn Ohlsson	Karlshamn hospital
Peter Andersson<br>Staffan Jangmalm	Växjö hospital

9. Main study publications

Planned and published main studies of SCREESCO:

- Compliance, findings and negative effects of the screening initiative (34)
- Emotional impact of screening on participants and non-participants (35)
- Quality assurance of screening colonoscopy (36)
- Study of intervention compared to control: adverse events and incident colorectal cancers during the intervention phase
- Health economy of colorectal cancer screening implementation
- Main analysis of the primary end-point (mortality)

See Section 3.7 for a list of secondary studies.

10. Scientific committee

Original list of members of the Scientific Committee

Name	Country	Area of expertise
Rolf Hultcrantz	Sweden	Gastroenterology
Lars Holmberg	Sweden	Screening, surgery
Anders Ekbom	Sweden	Epidemiology
Anna Forsberg	Sweden	Gastroenterology, endoscopy
Robert Steele	United Kingdom	Screening, Surgery
Richard Palmqvist	Sweden	Pathology
Mef Nilbert	Sweden	Molecular oncology
Andreas Pischel	Sweden	Endoscopy
Marc Buyse	Belgium	Biostatistics
Yvonne Wengström	Sweden	Qualitative research
Per Carlsson	Sweden	Health economy
Lars Engstrand	Sweden	Microbiota
Johannes Blom	Sweden	Screening, surgery

Updated list of members of the Scientific Committee (May 06, 2021)

Name	Country	Area of expertise
Rolf Hultcrantz	Sweden	Gastroenterology
Lars Holmberg	Sweden	Screening, surgery
Anders Ekbom	Sweden	Epidemiology
Anna Forsberg	Sweden	Gastroenterology, endoscopy
Robert Steele	United Kingdom	Screening, Surgery
Chris Metcalfe	United Kingdom	Medical Statistics
Christian Löwbeer	Sweden	Laboratory medicine, clinical chemistry
Andreas Pischel	Sweden	Endoscopy
Yvonne Wengström	Sweden	Qualitative research
Lars-Åke Levin	Sweden	Health economy
Lars Engstrand	Sweden	Microbiota
Johannes Blom	Sweden	Screening, surgery
Mikael Hellström	Sweden	Radiology
Kaisa Fritzell	Sweden	Qualitative research
Ulf Strömberg	Sweden	Epidemiology

Updated list of members of the Scientific Committee (Nov 26, 2024)

Name	Country	Area of expertise
Lars Holmberg	Sweden	Screening, surgery
Anna Forsberg	Sweden	Gastroenterology, endoscopy
Marcus Westerberg	Sweden	Medical Statistics
Jonas F Ludvigsson	Sweden	Epidemiology
Robert Steele	United Kingdom	Screening, Surgery
Chris Metcalfe	United Kingdom	Medical Statistics
Christian Löwbeer	Sweden	Lab medicine, clinical chemistry
Lars Engstrand	Sweden	Microbiota
Johannes Blom	Sweden	Screening, surgery
Mikael Hellström	Sweden	Radiology
Kaisa Fritzell	Sweden	Qualitative research
Ulf Strömberg	Sweden	Epidemiology

11. Figures

11.2 Figure 1. Flow chart of invitation procedure of individuals randomized to intervention primary colonoscopy

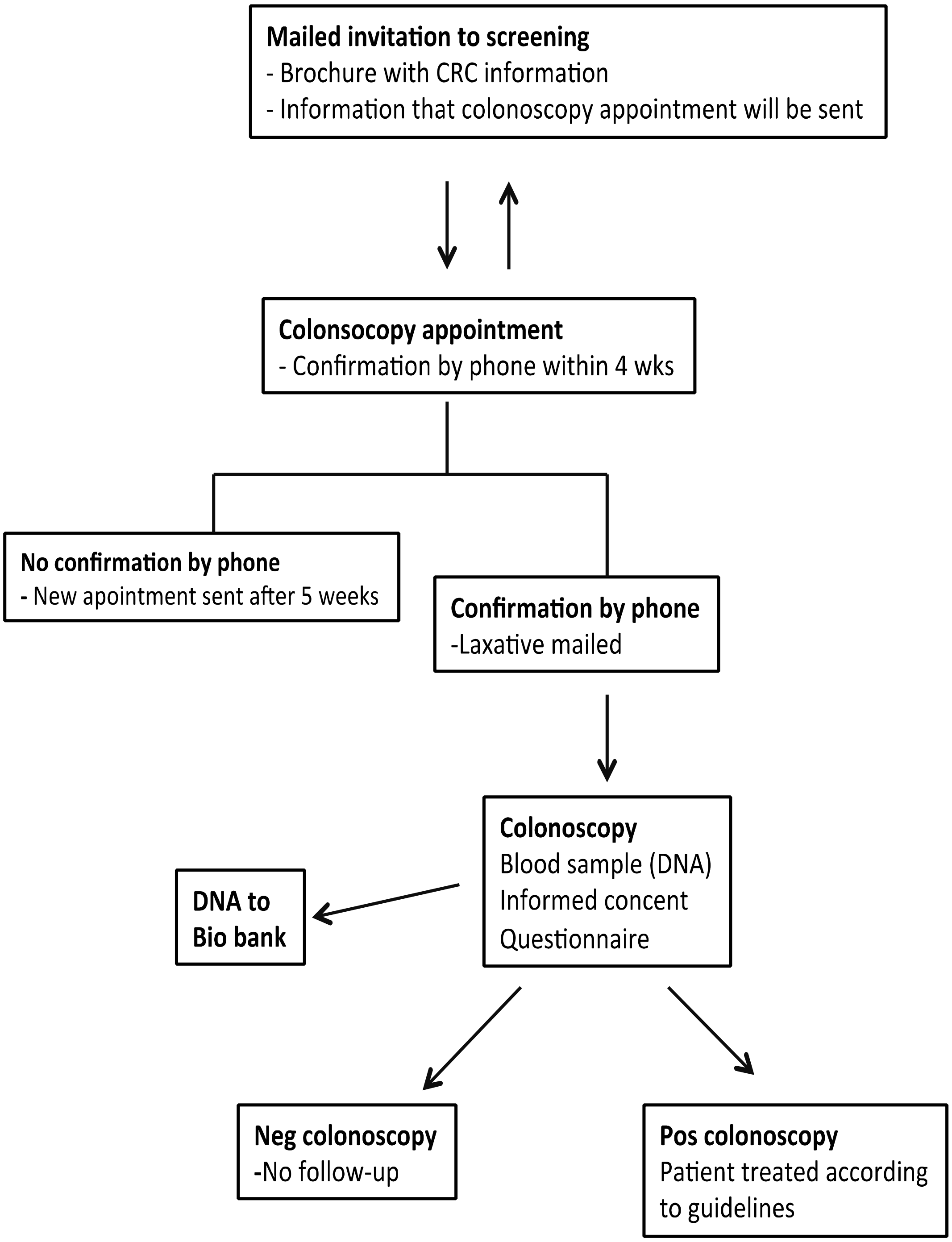

11.1 Figure 2. Flow chart of invitation procedure of individuals randomized to intervention FIT

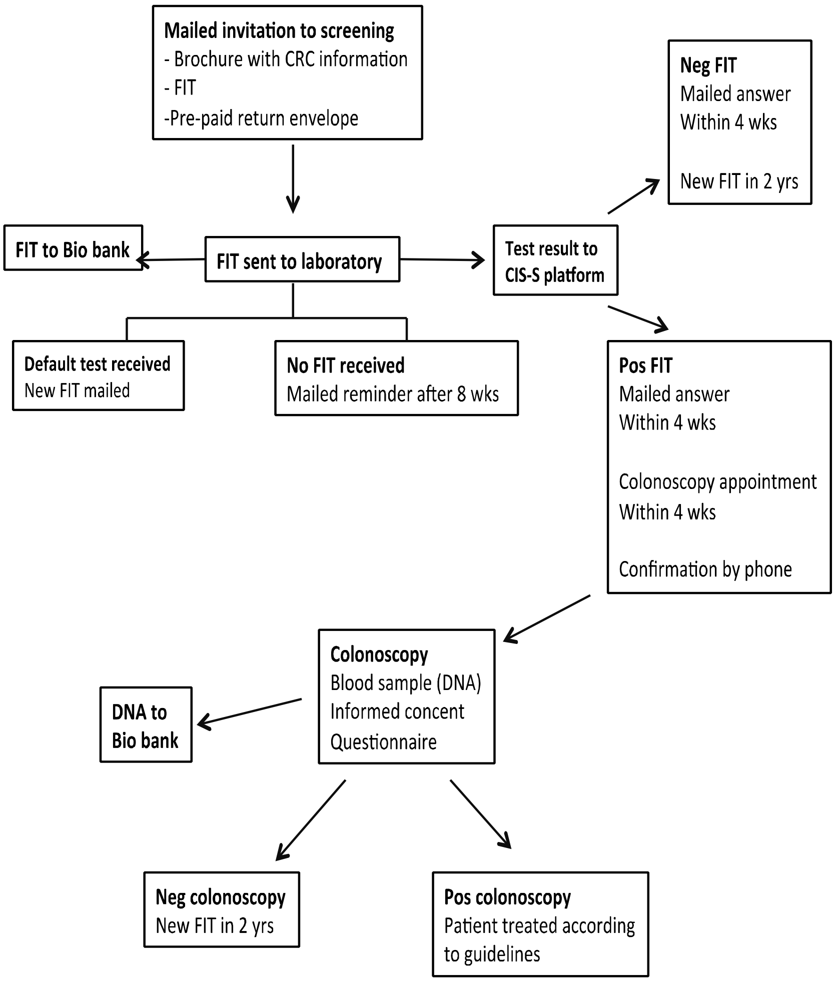

12. References

1. The Swedish Cancer Register http://www.socialstyrelsen.se/register/halsodataregister/cancerregistret/inenglish: National Board of Health and Welfare; [cited 2013 December]
2. Muto T, Bussey HJ, Morson BC. The evolution of cancer of the colon and rectum. Cancer. 1975;36(6):2251-70.
3. Vogelstein B, Fearon ER, Hamilton SR, Kern SE, Preisinger AC, Leppert M, et al. Genetic alterations during colorectal-tumor development. N Engl J Med. 1988;319(9):525-32.
4. Winawer SJ, Zauber AG, Ho MN, O'Brien MJ, Gottlieb LS, Sternberg SS, et al. Prevention of colorectal cancer by colonoscopic polypectomy. The National Polyp Study Workgroup. N Engl J Med. 1993;329(27):1977-81.
5. Muller AD, Sonnenberg A. Prevention of colorectal cancer by flexible endoscopy and polypectomy. A case-control study of 32,702 veterans. Ann Intern Med. 1995;123(12):904-10.
6. Thiis-Evensen E, Hoff GS, Sauar J, Langmark F, Majak BM, Vatn MH. Population-based surveillance by colonoscopy: effect on the incidence of colorectal cancer. Telemark Polyp Study I. Scand J Gastroenterol. 1999;34(4):414-20.
7. Armitage N. Screening for cancer. In: Morris D, Kearsley, J, Williams, C, editor. Cancer: a comprehensive clnical guide. pp 9-20. U.K.: Taylor and Francis Ltd 1997.
8. Mandel JS, Bond JH, Church TR, Snover DC, Bradley GM, Schuman LM, et al. Reducing mortality from colorectal cancer by screening for fecal occult blood. Minnesota Colon Cancer Control Study. N Engl J Med. 1993;328(19):1365-71.
9. Kronborg O, Fenger C, Olsen J, Jorgensen OD, Sondergaard O. Randomised study of screening for colorectal cancer with faecal-occult-blood test. Lancet. 1996;348(9040):1467-71.
10. Hardcastle JD, Chamberlain JO, Robinson MH, Moss SM, Amar SS, Balfour TW, et al. Randomised controlled trial of faecal-occult-blood screening for colorectal cancer. Lancet. 1996;348(9040):1472-7.
11. Lindholm E, Brevinge H, Haglind E. Survival benefit in a randomized clinical trial of faecal occult blood screening for colorectal cancer. Br J Surg. 2008;95(8):1029-36.
12. Atkin WS, Edwards R, Kralj-Hans I, Wooldrage K, Hart AR, Northover JM, et al. Once-only flexible sigmoidoscopy screening in prevention of colorectal cancer: a multicentre randomised controlled trial. Lancet. 2010;375(9726):1624-33.
13. Segnan N, Armaroli P, Bonelli L, Risio M, Sciallero S, Zappa M, et al. Once-only sigmoidoscopy in colorectal cancer screening: follow-up findings of the Italian Randomized Controlled Trial--SCORE. J Natl Cancer Inst. 2011;103(17):1310-22.
14. Schoen RE, Pinsky PF, Weissfeld JL, Yokochi LA, Church T, Laiyemo AO, et al. Colorectal-cancer incidence and mortality with screening flexible sigmoidoscopy. N Engl J Med. 2012;366(25):2345-57.
15. Faivre J, Bouvier AM, Bonithon-Kopp C. Epidemiology and screening of colorectal cancer. Best Pract Res Clin Gastroenterol. 2002;16(2):187-99.

16. Richardson A. Screening and the number needed to treat. J Med Screen. 2001;8(3):125-7.
17. Bretthauer M. Colorectal cancer screening. J Intern Med. 2011;270(2):87-98.
18. Brouwers MC, De Vito C, Bahirathan L, Carol A, Carroll JC, Cotterchio M, et al. What implementation interventions increase cancer screening rates? a systematic review. Implementation science : IS. 2011;6:111.
19. Tinmouth J, Ritvo P, McGregor SE, Claus D, Pasut G, Myers RE, et al. A qualitative evaluation of strategies to increase colorectal cancer screening uptake. Can Fam Physician. 2011;57(1):e7-15.
20. Blom J, Liden A, Jeppsson B, Holmberg L, Pahlman L. Compliance and findings in a Swedish population screened for colorectal cancer with sigmoidoscopy. Eur J Surg Oncol. 2002;28(8):827-31.
21. Blom J, Liden A, Nilsson J, Pahlman L, Nyren O, Holmberg L. Colorectal cancer screening with flexible sigmoidoscopy-participants' experiences and technical feasibility. Eur J Surg Oncol. 2004;30(4):362-9.
22. Collins RE, Lopez LM, Marteau TM. Emotional impact of screening: a systematic review and meta-analysis. BMC Public Health. 2011;11:603.
23. Tornberg S, Lundstrom V, Gustafsson S, Hultkrantz R. [The first year with colorectal cancer screening in Stockholm. Careful monitoring and quality control of the whole process is necessary]. Lakartidningen. 2010;107(26-28):1709-11.
24. Foss C, Ellefsen B. The value of combining qualitative and quantitative approaches in nursing research by means of method triangulation. J Adv Nurs. 2002;40(2):242-8.
25. Ostlund U, Kidd L, Wengstrom Y, Rowa-Dewar N. Combining qualitative and quantitative research within mixed method research designs: a methodological review. Int J Nurs Stud. 2011;48(3):369-83.
26. von Karsa L, Patnick J, Segnan N. European guidelines for quality assurance in colorectal cancer screening and diagnosis. First Edition--Executive summary. Endoscopy. 2012;44 Suppl 3:SE1-8.
27. Register of the total population https://www.skatteverket.se/download/18.2b543913a42158acf80003761/1386594730871/AllmänBeskrivning.pdf: Swedish tax agency; [
28. Kaminski MF, Bretthauer M, Zauber AG, Kuipers EJ, Adami HO, van Ballegooijen M, et al. The NordICC Study: rationale and design of a randomized trial on colonoscopy screening for colorectal cancer. Endoscopy. 2012;44(7):695-702.
29. Patient register http://www.socialstyrelsen.se/register/halsodataregister/patientregistret/inenglish: National Board of Health and Welfare; [
30. Causes of Death register http://www.socialstyrelsen.se/register/dodsorsaksregistret: National Board of Health and Welfare; [
31. Wallace MB, Kiesslich R. Advances in endoscopic imaging of colorectal neoplasia. Gastroenterology. 2010;138(6):2140-50.
32. Hamilton SR AL. Pathology and Genetics. Tumours of the Digestive System, WHO Classification of tumours, vol. 2. IARC WHO, Classification of tumours, No 2; 20.

33. Wallerstedt SM, Wettermark B, Hoffmann M. The first decade with the Swedish prescribed drug register–a systematic review of the output in the scientific literature. Basic & clinical pharmacology & toxicology. 2016;119(5):464-9.
34. Forsberg A, Westerberg M, Metcalfe C, Steele R, Blom J, Engstrand L, et al. Once-only colonoscopy or two rounds of faecal immunochemical testing 2 years apart for colorectal cancer screening (SCREESCO): preliminary report of a randomised controlled trial. Lancet Gastroenterol Hepatol. 2022;7(6):513-21.
35. Fritzell K, Forsberg A, Wangmar J, Wengström Y, Bottai M, Hultcrantz R. Gender, having a positive FIT and type of hospital are important factors for colonoscopy experience in colorectal cancer screening–findings from the SCREESCO study. Scandinavian Journal of Gastroenterology. 2020;55(11):1354-62.
36. Sekiguchi M, Westerberg M, Ekbom A, Hultcrantz R, Forsberg A. Endoscopist Characteristics and Polyp Detection in Colonoscopy: Cross-Sectional Analyses of Screening of Swedish Colons. Gastroenterology. 2023;164(2):293-5 e4.

13. Summary of changes to the SCREESCO study protocol and statistical analysis plan 2013-2024

Changes in the study protocol version 2.0

Study protocol was amended after new power-calculation due to anticipated 35% participation in the colonoscopy arm. In Swedish 2017-03-10, translated to English 2021-04-29. List of members Scientific Committee was updated.

Changes in the study protocol version 3.0

Study protocol was amended after the Scientific Committee decided on a last date of follow-up based on new power calculations. It was also decided that the previously described interim analysis will not be performed. The list of main publications was updated, and so was the list of members of the Scientific Committee. A summary of changes of the protocol and statistical analysis plan was added. Weblinks under *Head Secretariat* were updated.

Changes in the statistical analysis plan 2.0

Statistical analysis plan was amended after new power-calculation due to anticipated 35% participation in the colonoscopy arm.

Changes in the statistical analysis plan 3.0

Statistical analysis plan was amended on 2024-11-04 after the Scientific Committee decided on a last date of follow-up based on new power calculations. It was also decided that the previously described interim analysis will not be performed. Details and clarifications regarding the initial and modified power calculations were added. The analysis of incidence of colorectal cancer was changed and is now based on cumulative incidence curves instead of the log-rank test.

SCREESCO statistical analysis plan

This charter defines the main analyses on which conclusions regarding main study aims 1-3 as defined in the protocol February 19 2012. Compliance as a secondary endpoint is included here since it is anticipated that compliance will be an indicator of feasibility that the health authorities want reported at the earliest point in time when data can be deemed informative.

Study design

SCREESCO is a population based randomized screening trial for colorectal cancer with three arms: Invitation to direct colonoscopy, invitation to FIT test or control. All counties and regions in Sweden except the Stockholm region and Västernorrland's county enroll participants. Eligible are persons in the Swedish Population register for the calendar year they turn 60. Follow-up is at least 15 years.

Interventions

The three study arms are: written invitation to direct colonoscopy and within 8 weeks an offer to participation with a booked appointment; invitation to FIT test and test equipment sent for sampling at home at inclusion and at 2 years after inclusion, offering a colonoscopy after a positive test; control group given standard diagnostic work-up and care for colorectal cancer.

Study aims concerned in the statistical analysis plan

Main aims

1/Analyze the effect of invitation to colorectal cancer screening on colorectal cancer mortality

2/ Analyze the effect of invitation to colorectal cancer screening on colorectal cancer incidence

3/ To supply evidence if either direct invitation to colonoscopy or invitation to FIT test should be the method of choice for colorectal cancer screening in Sweden, should a national screening program be initiated.

Secondary aims

Describe and quantify compliance to screening by study arm

Dimensioning of the study

Assumptions

SCREESCO assumes 1% cumulative colorectal cancer mortality for a follow-up between 60 and 75 years of age. In accordance with earlier international literature, a 30% relative reduction in colorectal cancer mortality is expected following screening with FIT and colonoscopy when FIT is positive, and a 50% relative reduction in colorectal cancer mortality following screening with direct colonoscopy. Compliance is expected to be 50% in the FIT arm, 10% to have a positive test and 90% of those to undergo colonoscopy. Compliance is expected to be 50% in the direct colonoscopy arm. Screening in the control group is assumed to be negligible.

Study size

For a 80% power at a significance level of 2.5% 20 100 persons should be randomized to be invited to direct colonoscopy, 60 186 to be invited to FIT and 120 372 to control in an analysis at 15 years after randomization. Each of the intervention arms will be compared to the control group.

Table 1 shows the association between study precision and compliance.

Definition of study population

A population sample will be drawn from the Swedish Population Register for the participating counties in 2014, 2015 and 2016 for persons born 1954, 1955 and 1966 respectively. In each sample 6 700 will be allocated to direct colonoscopy, for each of those three persons matched on county and gender will be allocated to FIT and six persons likewise matched on county and gender will be allocated to control.

Each sample will be matched to the Swedish Cancer Register and individuals with a colorectal and/or anal cancer before or at the date the sample was drawn will be excluded. In regions participating in the NordiCC trial, study subjects of NordiCC will be excluded.

Follow-up starts at date of randomization. A study participant is lost to follow-up at date of emigration or at date of actively withdrawing consent to be followed through registers.

Definition of exposure, co-variates and endpoints

Follow-up

Follow-up for the main endpoints is register-based. The following registers will be used: The National Cancer Register, the National In-patient Register, The National Clinical database for Colorectal cancer, the National Causes of Death register.

Compliance is registered by the study log of invitations and procedures undertaken. Currently there is no full register coverage of use of FIT or colonoscopy in the general population and thus not for the control group. The colonoscopy activity in the control arm and the intervention arm outside the study will be estimated by using data from regions where there is a well-functioning out-patient register.

Exposure

The exposure of interest is *invitation* to either screening arm; the control group serves as comparator separately for each arm.

Endpoints

For study aim 1 as above, death from colorectal cancer as defined in the Swedish Cause of Death Register as cause of death or as contributing cause of death is the endpoint of interest. Deaths due to complications of diagnostic or therapeutic interventions directed to colorectal cancer will be counted as deaths from colorectal cancer.

For study aim 2 as above, incident cases of invasive colorectal cancer as registered to Swedish Cancer Register is the endpoint of interest. Stage of invasive disease as reported to the national Swedish clinical database for colorectal cancer will be reported.

For study aim 3 as above, the study will provide a broad spectrum of data for a cost-effectiveness analysis with reduction of colon cancer mortality as the measure of effect.

Compliance is defined as being adherent to colonoscopy either by direct invitation or being adherent to colonoscopy or other diagnostic procedure after a positive FIT test. The time-window considered will be six months after invitation.

Co-variates

Age, gender and county of residence are accounted for in the study design. For the main analyses, no further co-variates will be adjusted for.

Analyses of compliance will be stratified on age, gender and county of residence.

Validation of endpoints

A random sample of full medical case records for in total 100 deaths of colorectal cancer will be drawn to validate death certificates as reported to the causes of death registry. The deaths will be sampled prospectively during the study, drawing 20 cases each year starting year 5 of study follow-up. The Data Monitoring and Safety Committee (se below) will review the validation and recommend changes in endpoint ascertainment or suggest sensitivity analyses of the main outcomes if needed.

Statistical methods

Mortality

As the main analysis, net probability of death from colorectal cancer will be estimated. The events of interest will be death from colorectal cancer or by complications to the screening procedure and/or treatment of colorectal cancer. The date of randomization will be used as start of follow-up and the log rank test will be used as the test of significance at a 2.5% level (two-sided).

In the main analysis the two screening arms will each separately be compared to the control arm.

All analyses to address the main aims 1-3 above will be based on random allocation to study arm, i.e. an intention to screen analysis.

As a subsidiary analysis, net probability of mortality of all causes will be estimated.

Incidence

As the main analysis, net probability of invasive colorectal cancer will be estimated. The date of randomization will be used as start of follow-up and the log rank test will be used as the test of significance at a 5% level.

The two screening arms will each separately be compared to the control arm.

A subsidiary analysis will estimate the net probability of advanced colorectal cancer (Duke's stage B and higher).

Compliance

Compliance in screening as a whole and by screening intervention arm will be estimated within one year after completion of the second FIT round for the last included individuals. The result will be

presented as a proportion with 95% confidence interval based on that the study population is a random sample of the whole base population in the participating counties as present July 1 2014.

Usage of colonoscopy in the control arm and in the intervention arms outside the study protocol will be estimated as described under follow-up and will be presented with – but not adjust – the main analysis.

Supporting a choice of screening method

As for study aim 3 as above, the analyses will be based on data from both arms on mortality, incidence, compliance, participant's experience, side-effects and further supported by health economics analyses and modelling. Thus, any recommendation will be based on a broad set of criteria of health effects and public health considerations.

Timing of the analyses

SCREESCO is powered for a main analysis 15 years after randomization. An analysis of compliance as described above is planned within one year after completion of the second FIT test for the last included cohort. Development of serious adverse events will be followed continuously, but will also be summarized at 15 years.

An interim analysis of the main outcome colorectal cancer mortality will be performed at 10 years of follow-up using the O'Brien-Fleming alpha spending function in order to keep the overall type I error for each of the comparisons of the screening arms to the control arm at 2.5% (see also below under Safety).

Presentation in main report

The following data will be presented in the main analysis: CONSORT diagram of study design; baseline characteristics by study arm; compliance to invitation and diagnostic work-up; estimate of colonoscopies in control arm (see above under follow-up); serious adverse events; characteristics of polyps detected in screening program; characteristics of precancerous lesions and cancers by all three study arms; net probability of invasive colorectal cancer in all three study arms; net probability of death from colorectal cancer and overall mortality in all three study arms.

Safety

The trial steering committee will appoint a Data Monitoring and Safety Committee (DMSC), which will define a DMSC charter and need for analyses. The charter will be presented to and approved by the trial steering committee.

The trial steering committee will follow serious adverse events on a continuous basis and act promptly on any signs of threat to safety within centers or for the trial as a whole. Additional unblinded analyses of the main endpoint will be taken into account regarding the usage of the O'Brien-Fleming alpha spending function.

Table 1, association between study precision and compliance

**Arm A (colonoscopy N=20 100)<br>(assumed CRC mortality reduction<br>at 100% compliance = 50%)**		**Arm B (FIT N=60 186)<br>(assumed CRC mortality reduction<br>at 100% compliance = 30%)**

Compliance	Efficacy	Power (α=2.5%)		Compliance	Efficacy	Power (α=2.5%)
60%	30,0%	96,3%		60%	18,0%	93,0%
55%	27,5%	92,5%		55%	16,5%	87,7%
50%	25,0%	86,4%		**50%**	**15,0%**	**80,0%**
45%	22,5%	77,6%		45%	13,5%	70,1%
40%	20,0%	66,3%		40%	12,0%	58,4%
35%	17,5%	53,4%		35%	10,5%	46,0%
30%	15,0%	40,1%		30%	9,0%	34,1%
25%	12,5%	27,9%		25%	7,5%	23,6%
20%	10,0%	17,9%		20%	6,0%	15,2%

Table 1 shows efficacy in an intention to screen analysis and the corresponding power for different levels of compliance by study arm. The power is calculated based on the study design to compare each arm separately to the control group (N=120 372).

SCREESCO statistical analysis plan 2.0

This charter defines the main analyses on which conclusions regarding main study aims 1-3 as defined in the protocol February 19 2012. Compliance as a secondary endpoint is included here since it is anticipated that compliance will be an indicator of feasibility that the health authorities want reported at the earliest point in time when data can be deemed informative.

Study design

SCREESCO is a population based randomized screening trial for colorectal cancer with three arms: Invitation to direct colonoscopy, invitation to FIT test or control. All counties and regions in Sweden except the Stockholm region and Västernorrland's county enroll participants. Eligible are persons in the Swedish Population register for the calendar year they turn 60. Follow-up is at least 15 years.

Interventions

The three study arms are: written invitation to direct colonoscopy and within 8 weeks an offer to participation with a booked appointment; invitation to FIT test and test equipment sent for sampling at home at inclusion and at 2 years after inclusion, offering a colonoscopy after a positive test; control group given standard diagnostic work-up and care for colorectal cancer.

Study aims concerned in the statistical analysis plan

Main aims

1/Analyze the effect of invitation to colorectal cancer screening on colorectal cancer mortality.

2/ Analyze the effect of invitation to colorectal cancer screening on colorectal cancer incidence.

3/ To supply evidence if either direct invitation to colonoscopy or invitation to FIT test should be the method of choice for colorectal cancer screening in Sweden, should a national screening program be initiated.

Secondary aims

Describe and quantify compliance to screening by study arm.

Dimensioning of the study

Assumptions

SCREESCO assumes 1% cumulative colorectal cancer mortality for a follow-up between 60 and 75 years of age. In accordance with earlier international literature, a 30% relative reduction in colorectal cancer mortality is expected following screening with FIT and colonoscopy when FIT is positive, and a 50% relative reduction in colorectal cancer mortality following screening with direct colonoscopy. Compliance is expected to be 50% in the FIT arm, 10% to have a positive test and 90% of those to undergo colonoscopy. Compliance is expected to be 50% in the direct colonoscopy arm. Screening in the control group is assumed to be negligible.

Study size

For a 80% power at a significance level of 2.5% 20 100 persons should be randomized to be invited to direct colonoscopy, 60 186 to be invited to FIT and 120 372 to control in an analysis at 15 years after randomization. Each of the intervention arms will be compared to the control group.

Table 1 shows the association between study precision and compliance.

Definition of study population

A population sample will be drawn from the Swedish Population Register for the participating counties in 2014, 2015 and 2016 for persons born 1954, 1955 and 1956 respectively. In each sample 6 700 will be allocated to direct colonoscopy, for each of those three persons matched on county and gender will be allocated to FIT and six persons likewise matched on county and gender will be allocated to control.

Each sample will be matched to the Swedish Cancer Register and individuals with a colorectal and/or anal cancer before or at the date the sample was drawn will be excluded. In regions participating in the NordiCC trial, study subjects of NordiCC will be excluded.

Follow-up starts at date of randomization. A study participant is lost to follow-up at date of emigration or at date of actively withdrawing consent to be followed through registers.

Definition of exposure, co-variates and endpoints

Follow-up

Follow-up for the main endpoints is register-based. The following registers will be used: The National Cancer Register, the National In-patient Register, The National Clinical database for Colorectal cancer, the National Causes of Death register.

Compliance is registered by the study log of invitations and procedures undertaken. Currently there is no full register coverage of use of FIT or colonoscopy in the general population and thus not for the control group. The colonoscopy activity in the control arm and the intervention arm outside the study will be estimated by using data from regions where there is a well-functioning out-patient register.

Exposure

The exposure of interest is *invitation* to either screening arm; the control group serves as comparator separately for each arm.

Endpoints

For study aim 1 as above, death from colorectal cancer as defined in the Swedish Cause of Death Register as cause of death or as contributing cause of death is the endpoint of interest. Deaths due to complications of diagnostic or therapeutic interventions directed to colorectal cancer will be counted as deaths from colorectal cancer.

For study aim 2 as above, incident cases of invasive colorectal cancer as registered to Swedish Cancer Register is the endpoint of interest. Stage of invasive disease as reported to the national Swedish clinical database for colorectal cancer will be reported.

For study aim 3 as above, the study will provide a broad spectrum of data for a cost-effectiveness analysis with reduction of colon cancer mortality as the measure of effect.

Compliance is defined as being adherent to colonoscopy either by direct invitation (colonoscopy arm) or returning a FIT test (FIT arm). The time-window considered will be six months after invitation.

Co-variates

Age, gender and county of residence are accounted for in the study design. For the main analyses, no further co-variates will be adjusted for.

Analyses of compliance will be stratified on age, gender and county of residence.

Validation of endpoints

A random sample of full medical case records for in total 100 deaths of colorectal cancer will be drawn to validate death certificates as reported to the causes of death registry. The deaths will be sampled prospectively during the study, drawing 20 cases each year starting year 5 of study follow-up. The Data Monitoring and Safety Committee (se below) will review the validation and recommend changes in endpoint ascertainment or suggest sensitivity analyses of the main outcomes if needed.

Statistical methods

All main analyses to address the aims 1-3 above will be based on random allocation to study arm, i.e. an intention to screen analysis. Other analyses are subsidiary.

Mortality

As the main analysis, net probability of death from colorectal cancer will be estimated. The events of interest will be death from colorectal cancer or by complications to the screening procedure and/or treatment of colorectal cancer. The date of randomization will be used as start of follow-up and the log rank test will be used as the test of significance at a 2.5% level (two-sided).

In the main analysis the two screening arms will each separately be compared to the control arm.

As a subsidiary analysis, net probability of mortality of all causes will be estimated.

Incidence

As the main analysis, net probability of invasive colorectal cancer will be estimated. The date of randomization will be used as start of follow-up and the log rank test will be used as the test of significance at a 5% level.

The two screening arms will each separately be compared to the control arm.

A subsidiary analysis will estimate the net probability of advanced colorectal cancer (Duke's stage B and higher).

Compliance

Compliance in screening as a whole and by screening intervention arm will be estimated within one year after completion of the second FIT round for the last included individuals. The result will be presented as a proportion with 95% confidence interval based on that the study population is a random sample of the whole base population in the participating counties as present July 1 2014.

Usage of colonoscopy in the control arm and in the intervention arms outside the study protocol will be estimated as described under follow-up and will be presented with – but not adjust – the main analysis.

Supporting a choice of screening method

As for study aim 3 as above, the analyses will be based on data from both arms on mortality, incidence, compliance, participant's experience, side-effects and further supported by health economics analyses and modelling. Thus, any recommendation will be based on a broad set of criteria of health effects and public health considerations.

Timing of the analyses

SCREESCO is powered for a main analysis 15 years after randomization. An analysis of compliance as described above is planned within one year after completion of the second FIT test for the last included cohort. Development of serious adverse events will be followed continuously, but will also be summarized at 15 years.

An interim analysis of the main outcome colorectal cancer mortality will be performed at 10 years of follow-up using the O'Brien-Fleming alpha spending function in order to keep the overall type I error for each of the comparisons of the screening arms to the control arm at 2.5% (see also below under Safety).

Presentation in main report

The following data will be presented in the main analysis: CONSORT diagram of study design; baseline characteristics by study arm; compliance to invitation and diagnostic work-up; estimate of colonoscopies in control arm (see above under follow-up); serious adverse events; characteristics of polyps detected in screening program; characteristics of precancerous lesions and cancers by all three study arms; net probability of invasive colorectal cancer in all three study arms; net probability of death from colorectal cancer and overall mortality in all three study arms.

Safety

The trial steering committee will appoint a Data Monitoring and Safety Committee (DMSC), which will define a DMSC charter and need for analyses. The charter will be presented to and approved by the trial steering committee.

The trial steering committee will follow serious adverse events on a continuous basis and act promptly on any signs of threat to safety within centers or for the trial as a whole. Additional unblinded analyses of the main endpoint will be taken into account regarding the usage of the O'Brien-Fleming alpha spending function.

Table 1, association between study precision and compliance

**Arm A (colonoscopy N=20 100) (assumed CRC mortality reduction at 100% compliance = 50%)**				**Arm B (FIT N=60 186) (assumed CRC mortality reduction at 100% compliance = 30%)**		
Compliance	Efficacy	Power (α=2.5%)		Compliance	Efficacy	Power (α=2.5%)
60%	30,0%	96,3%		60%	18,0%	93,0%

55%	27,5%	92,5%		55%	16,5%	87,7%
50%	25,0%	86,4%		**50%**	**15,0%**	**80,0%**
45%	22,5%	77,6%		45%	13,5%	70,1%
40%	20,0%	66,3%		40%	12,0%	58,4%
35%	17,5%	53,4%		35%	10,5%	46,0%
30%	15,0%	40,1%		30%	9,0%	34,1%
25%	12,5%	27,9%		25%	7,5%	23,6%
20%	10,0%	17,9%		20%	6,0%	15,2%

Table 1 shows efficacy in an intention to screen analysis and the corresponding power for different levels of compliance by study arm. The power is calculated based on the study design to compare each arm separately to the control group (N=120 372).

Addendum Sept 22 2016

In the original sample size calculation, 50% compliance was assumed in both Arm A (colonoscopy) and arm B (FIT). With an assumed CRC mortality reduction of 30% in Arm B (FIT) at 100% compliance (15% reduction at 50% compliance), and a 1:2 ratio (Arm B : controls) randomization, 60 300 individuals were to be randomized to Arm B (FIT) and 120 600 to the control arm in order to achieve 80% power with alpha = 0.025. If a third of the number of patients randomized to Arm B (FIT), i.e. 20 100, were randomized to Arm A (colonoscopy) we would have more than 80% power (~86%) to detect a CRC mortality reduction of 50% at 100% compliance (25% reduction at 50% compliance) in Arm A (colonoscopy).

Recent analyses of compliance in Arm A has suggested a compliance of 35%. This results in the power dropping to 53%.

The number of colonoscopies before increasing the size of the colonoscopy arm is assumed to be roughly 7 035 (20 100*35%), i.e. 3 015 less than the 10 050 initially planned for.

SCREESCO will increase the size of arm A by 10 000 (and increasing the control arm C correspondingly by 6*10 000) and thus increase the power to 73%, assuming compliance of 35%. This will result in 3 500 more colonoscopies (10 000*35%) but will only increase the number of expected colonoscopies originally planned for by 485 (20 100*35% + 10000*35% = 10 535; 10 535 – 10 050 = 485).

SCREESCO
Statistical Analysis Plan 3.0

Prepared by

Marcus Westerberg, PhD

Chris Metcalfe, PhD

Anders Ekbom, MD, PhD

Christian Löwbeer, MD, PhD

Robert Steele, MD, PhD

Ulf Strömberg, PhD

Jonas F. Ludvigsson, MD, PhD

Lars Holmberg, MD, PhD

Anna Forsberg, MD, PhD

The randomized controlled trial

Screening of Swedish Colons (SCREESCO)

About the Statistical Analysis Plan version 3.0

This charter defines the main analyses on which conclusions regarding main study aims 1-3 as defined in the protocol February 19 2012. Compliance as a secondary endpoint is included here since compliance is an indicator of feasibility to report to the health authorities as soon as data are informative.

Motivation for version 3.0

On February 8 2024, the SCREESCO scientific committee assessed the impact of choice of calendar time of end of follow-up on the trade-off between timeliness of reporting results and statistical precision in the analysis of primary endpoints.

Key changes

- Added details and clarifications regarding the initial and modified power calculations.
- An assessment of optimal time for evaluation of the primary endpoints based on new thorough power calculations.

Study design

SCREESCO is a randomized screening trial for colorectal cancer with three arms: Invitation to direct colonoscopy, invitation to FIT test, or control. All counties and regions in Sweden except the Stockholm region and Västernorrland's county enroll participants. Eligible are persons in the Swedish Population register for the calendar year they turn 60. Follow-up is at least 15 years.

Interventions

The three study arms are: written invitation to direct colonoscopy and within 8 weeks an offer to participate with a booked appointment; invitation to FIT test and test equipment sent for sampling at home at inclusion and at 2 years after inclusion, offering a colonoscopy after a positive test; control group with standard diagnostic work-up and care for colorectal cancer.

Study aims concerned in the statistical analysis plan

Main aims

1/Analyze the effect of invitation to colorectal cancer screening on colorectal cancer mortality.

2/ Analyze the effect of invitation to colorectal cancer screening on colorectal cancer incidence.

3/ To supply evidence if either direct invitation to colonoscopy or invitation to FIT test should be the method of choice for colorectal cancer screening in Sweden, should a national screening program be initiated.

Secondary aims

Describe and quantify compliance to screening by study arm.

Dimensioning of the study

Assumptions

SCREESCO assumes 1% cumulative colorectal cancer mortality for a follow-up between 60 and 75 years of age. This estimate was based on publicly available historical data (until 2012) on causes of death (1). In accordance with earlier international literature on fecal occult-blood testing (2, 3, 4, 5), a 30% relative reduction in colorectal cancer mortality following screening with FIT and colonoscopy when FIT is positive was expected. A 50% relative reduction in colorectal cancer mortality following screening with direct colonoscopy was similarly expected based on previous trials on sigmoidoscopy (6, 7, 8).

Initially, compliance was expected to be 50% in the FIT arm, and 10% were expected to have a positive test and 90% of those were expected to undergo colonoscopy. Compliance was expected to be 50% in the direct colonoscopy arm. Screening in the control group was assumed to be negligible.

Initial calculation of study size

For an 80% power at a two-sided significance level of 2.5% for each pairwise comparison with the control group, 20 100 persons should be randomized to be invited to direct colonoscopy, 60 186 to be invited to FIT and 120 372 to control in an analysis at 15 years after randomization. Each of the intervention arms will be compared to the control group. Table 1 shows the association between study precision and compliance.

FIT vs control: For FIT vs control, 60 300 were randomized to FIT and 120 600 to control in order to obtain 80% power. We used alpha = 0.025, assumed 1 % mortality rate in arm C after 15 years, a ratio of 1:2, 50 % compliance, hazard ratio = 0.85 (15 % reduced mortality vs control: 30% effect and 50% compliance).

STATA command**:** stpower logrank 0.990, alpha(0.025) power(0.8) hratio(0.85) nratio(0.5)

PCOL vs control: For direct colonoscopy vs control, we used alpha = 0.025, 1 % mortality rate in the control arm after 15 years, a ratio 1:6 vs control, and 50 % compliance, hazard ratio = 0.75 (25% reduced mortality vs control: 50% effect and 50% compliance), and size of control arm of 120 600. Randomizing 1/3 of the size of the FIT arm to the direct colonoscopy arm, i.e. 20 100, gave power above 80 % (approximately 86%) when comparing to the control arm.

STATA command: stpower logrank 0.990, alpha(0.025) n(140700) hratio(0.75) nratio(0.1666)

Modified study size (2016)

In 2016, an analysis found that compliance was 35% in the direct colonoscopy arm implying that the power dropped to 53%. To increase power for the comparison of the direct colonoscopy arm vs control, the size of these arms were increased by extending the study to include birthyears 1957-1958. The ratio was fixed to 1:6, so that adding 10 000 extra individuals to the direct colonoscopy arm would result in approximately the same number of performed colonoscopies as originally planned, which was a tolerable amount for the colonoscopy sites to handle.

PCOL vs control: With an additional 60 000 to the control arm, hazard ratio = 0.825 (17.5 % reduced mortality vs control: 50% effect and 35% compliance), and n=120 600 + 60 000 + 20 100 + 10 000=210 700, then the power became 73%.

STATA command: stpower logrank 0.990, alpha(0.025) n(210700) hratio(0.825) nratio(0.16667)

Definition of study population

A population sample was drawn from the Swedish Population Register for the participating counties in 2014, 2015 and 2016 for persons born 1954, 1955 and 1956 respectively. In each sample 6 700 were allocated to direct colonoscopy, for each of those three persons matched on county and gender were allocated to FIT, and six persons likewise matched on county and gender were allocated to control. All individuals within each block defined by calendar year, county and gender were sampled and individually allocated to one of the three arms simultaneously. In 2016 we decided to randomize additional individuals born 1957 and 1958 to the direct colonoscopy arm and to the control arm during 2017 and 2018 using the same principles (**Table 2**).

Each sample was matched to the Swedish Cancer Register and individuals with a colorectal and/or anal cancer before or at the date the sample was drawn were excluded. In regions participating in the NordiCC trial, study subjects of NordiCC were excluded.

Follow-up starts at date of randomization. A study participant is lost to follow-up at date of emigration or at date of actively withdrawing consent to be followed through registers.

Definition of exposure, covariates and endpoints

Follow-up

Follow-up for the main endpoints is register-based. The following registers will be used: The National Cancer Register, the National In-patient Register, the National Out-patient Register, The National Clinical database for Colorectal cancer, the National Causes of Death register.

Compliance is registered by the study log of invitations and procedures undertaken. Currently there is no full register coverage of use of FIT or colonoscopy in the general population and thus not for the control group. The colonoscopy activity in the control arm and the intervention arm outside the study will be estimated by using data from The National Patient Registry.

Exposure

The exposure of interest is *invitation* to either screening arm; the control group serves as comparator separately for each arm.

Endpoints

For study main aim 1 as above, death from colorectal cancer as defined in the Swedish Cause of Death Register as cause of death or as contributing cause of death is the endpoint of interest. Deaths due to complications of diagnostic or therapeutic interventions directed to colorectal cancer will be counted as deaths from colorectal cancer.

For study main aim 2 as above, stage-specific colorectal cancer incidence is the endpoint of interest. Stage at diagnosis in the Swedish Cancer Register and/or the national Swedish colorectal cancer quality register will be reported.

For study main aim 3 as above, the study will provide a broad spectrum of data for a cost-effectiveness analysis with reduction of colon cancer mortality as the measure of effect.

Compliance is defined as being adherent to colonoscopy either by performing a screening colonoscopy after invitation (colonoscopy arm) or returning a FIT test (FIT arm), and has been assessed previously (9, 10).

Covariates

For the main analyses (intention-to-screen) no covariates will be adjusted for. A subgroup analysis in men and women will be performed.

Validation of endpoints

It was initially planned to perform a validation of death certificates using medical records. It is not clear however, that the effort to obtain medical records would be worthwhile given ethical, GDPR and administrative aspects. The scientific committee has instead decided to perform a register-based validation of colorectal cancer deaths as registered as the underlying cause of death in the cause of death register. We will assess evidence for (e.g. a diagnosis of advanced colorectal cancer close to date of death) and against death by colorectal cancer (e.g. low stage or no colorectal cancer diagnosis and high comorbidity) using data from other Swedish healthcare registries.

Statistical methods

All main analyses to address the aims 1-3 above will be based on random allocation to study arms, i.e. an intention to screen analysis. Other analyses are subsidiary.

Mortality

As the main analysis, net probability of death from colorectal cancer (in absence of competing causes) will be estimated using 1 minus the estimated survival curve when censoring for other causes of death, and presented with corresponding 95% confidence interval. The events of interest will be death from colorectal cancer or by complications to the screening procedure and/or treatment of colorectal cancer.

In the main analysis the two screening arms will each separately be compared to the control arm. The date of randomization will be used as start of follow-up and the log rank test will be used as the test of significance at a 2.5% level (two-sided).

As a subsidiary analysis, probability of mortality of all causes will be estimated.

Incidence

As the main analysis, cumulative incidence of colorectal cancer will be estimated, treating death from any cause as a competing event. The date of randomization will be used as start of follow-up. We will estimate the cumulative incidence of colorectal cancer and death from other causes in the primary colonoscopy arm vs control arm and in the FIT arm vs control arm non-parametrically, with 95% confidence intervals.

The log-rank test, as initially described, will not be used since we will compare cumulative incidence functions in a competing risk setting. We will also report absolute differences in cumulative incidence functions with 95% confidence intervals (11, 12).

A subsidiary analysis will similarly estimate the competing risk cumulative incidence of colorectal cancer by stage.

Use of colonoscopy outside of the study

Usage of colonoscopy (any purpose) in the control arm and in the intervention arms outside the study protocol will be estimated as described under follow-up and will be presented with the main analysis.

Supporting a choice of screening method

As for study main aim 3 as above, the analyses will be based on data from both arms on mortality, incidence, compliance, participant's experience, side-effects and further supported by health economics analyses and modelling. Thus, provision of evidence for the health authorities' future recommendation will be based on a broad set of criteria of health effects and public health considerations.

Timing of the analyses

SCREESCO was initially powered for a main analysis 15 years after randomization. Development of serious adverse events will be followed continuously, but will also be summarized at 15 years. The previous versions of the SAP and Study Protocol did not however specify when in calendar time the analysis should be performed.

Last date of follow-up for evaluation of primary endpoint

The Scientific Committee assessed the impact of choice of date for the end of follow-up on the statistical precision for comparing colorectal cancer specific mortality between each of the two active screening arms and the control arm. A conservative choice of date of end of follow-up is 15 years after the last invitation to screening (up to and including 2033-05-24). Statistical power did not meaningfully decrease by ending the follow-up somewhat earlier, already at 2030-12-31, so the scientific committee proposes this date as the last date of follow-up. We briefly describe the calculations underlying this decision in the following:

Aggregated and publicly available data from the National Board of Health and Welfare (Socialstyrelsen; SoS) and Statistics Sweden (SCB) between 2010 and 2014 (1, 13), before SCREESCO could have had any meaningful impact on mortality, was used to estimate separately for men and women the competing risk cumulative incidence functions of death by colorectal cancer and of other causes from age 60 to 80 years. These were computed using estimates of the competing risk hazard functions for time steps equal to 1 day. The hazard functions were used in combination with the assumed hazard ratios to define cumulative incidence functions for the direct colonoscopy and FIT arms.

The expected net risk of death from colorectal cancer in the control arm according to the model was 0.766% (0.877% in men and 0.654% in women) after 15 years of follow-up. This is lower than the 1% that was originally assumed for the power calculations. The risk of death from colorectal cancer has decreased over calendar time, both before and during SCREESCO, and the 1% estimate was based on more historical data than the new estimate. The corresponding risk of death from any cause was 16.1% (19.1% in men and 13.0% in women).

We therefore performed a sensitivity analysis (**Sensitivity Analysis 1**) where we increased the competing risk of death from colorectal cancer in the control arm by multiplying the cause-specific hazard with 1.3 to obtain an expected net risk of death from colorectal cancer of 1.002% (1.148% in

men and 0.856% in women) and corresponding risk of death from any cause of 16.3% (19.3% in men and 13.2% in women).

The cumulative incidence functions for the direct colonoscopy and FIT arms were in each scenario similarly obtained from the estimated hazard functions in the control arm after multiplication by the corresponding hazard ratios (0.825 and 0.85, respectively). We simulated time until death and cause of death for all randomized individuals in SCREESCO. Individuals were right censored at the first of 15 years from date of randomization, date of death from other causes and a date **X** indicating the last date of follow-up in calendar time. The dates **X** considered were 31st of December between 2024 and 2032, and in addition also the 24th of May from 2025 to 2033. In a second sensitivity analysis (**Sensitivity Analysis 2**), we instead allowed all individuals to contribute with follow-up until the end of the study, meaning that some may have been followed for more than 15 years.

The results of the simulation study are summarised in Table 3. For the comparison FITx2 vs CONTROL the power increased rapidly with later dates for the end of follow-up, from 35.4% when using 2024-12-31, to 64.4% at 2030-12-31, and remained around this percentage from this date onward, and the number of CRC deaths increased correspondingly from 641 to 1204. For the comparison of PCOL vs CONTROL the power was 28% and increased to 54.9% at the corresponding dates, and increased further to 58.7% at 2033-05-24. The corresponding number of CRC deaths were 692, 1392 and 1501.

Although the power in **Sensitivity Analysis 1** was higher, e.g. 77.5% at 2030-12-31 for FITx2 vs CONTROL and 67.8% for PCOL vs CONTROL, it only increased slightly after this date. The subgroup analyses had in all analyses much lower power (<40%) but the effect of choice of last date of follow-up was similar to that in the primary analysis.

In **Sensitivity Analysis 2** the power was comparable to the primary analysis at 2029-05-24 and was increasingly higher compared to in the primary analysis at later dates. At 2030-12-31 it increased to 68.4% for FITx2 vs CONTROL and 57.6% for PCOL vs CONTROL, and at 2033-05-24 it was 79.1% and 70.1% respectively.

Our estimates of power based on a lower net risk of death from colorectal cancer (0.766%) compared to previous power calculations (1%) produced slightly lower power but as a function of last date of follow-up the power did not materially change after 2030-12-31. This date allows almost all individuals to be followed for 15 years in the FITx2 arm, and the number of CRC deaths after this date is negligible in all arms. If follow-up may extend beyond 15 years from date of randomization, as in **Sensitivity Analysis 2**, there is a potential to gain some additional power at 2030-12-31 and even more at later dates.

The choice of date must balance the decrease in power against the potential value of providing evidence from the study and the potential impact of the findings on clinical practice. For this reason, we propose 2030-12-31 as the last date of follow-up rather than 2033-05-24, allowing publication of the primary endpoint almost 2.5 years earlier than 2033-05-24 with minimal impact on power.

Interim analysis

An interim analysis of the main outcome colorectal cancer mortality was previously specified to be performed at 10 years of follow-up using the O'Brien-Fleming alpha spending function in order to keep the overall type I error for each of the comparisons of the screening arms to the control arm at

2.5% (see also below under Safety). Based on the above calculations and the low power at 10 years, the scientific committee decided that this interim analysis should not be performed.

Presentation in main report

The following data will be presented in the main analysis: CONSORT diagram of study design; baseline characteristics by study arm; estimate of colonoscopies in control arm; serious adverse events; cumulative incidence of colorectal cancer (in total and by stage) in all three study arms; net probability of death from colorectal cancer and overall mortality in all three study arms.

Safety

In February 2024 it was obvious that the scientific committee has ensured the quality of the study regarding the database, and the adherence to the study protocol as well as the statistical analysis plan. Due to the long study time, the conditions have slightly changed and therefor updates have been required. The work of a Data Monitoring and Safety Committee has been performed by the study secretariat and scientific committee, taking responsibility for integrity and safety during the intervention phase of SCREESCO (between 2014 and 2020). Serious adverse events have been reported continuously and analyses have been undertaken of safety as well as participant experiences (9, 14). The study secretariat has with support from the sponsoring part – the Council of Regional Cancer Centers in Sweden – continuously updated the study database and checked it for accuracy. The integrity of the database has been checked in analyses of early aspects of the study (15, 16). The Scientific committee is not aware of and cannot access main outcomes by study arm. Only the study statistician can access the entire study database.

From 2020 and onward follow-up data are only based on registers, i.e., without any contact with or intervention directed to any randomized individuals. As a step towards transparency, the Study Protocol and the Statistical Analysis Plan will be published on clinicaltrials.gov, where the SCREESCO study is registered.

Table 1, association between study precision and compliance

**Arm A (colonoscopy N=20 100) (assumed CRC mortality reduction at 100% compliance = 50%)**				**Arm B (FIT N=60 186) (assumed CRC mortality reduction at 100% compliance = 30%)**		
Compliance (%)	Efficacy (%)	Power (%) at α=2.5%		Compliance (%)	Efficacy (%)	Power (%) at α=2.5%
60%	30.0%	96.3%		60%	18.0%	93.0%
55%	27.5%	92.5%		55%	16.5%	87.7%
50%	25.0%	86.4%		**50%**	**15.0%**	**80.0%**
45%	22.5%	77.6%		45%	13.5%	70.1%
40%	20.0%	66.3%		40%	12.0%	58.4%
35%	17.5%	53.4%		35%	10.5%	46.0%
30%	15.0%	40.1%		30%	9.0%	34.1%
25%	12.5%	27.9%		25%	7.5%	23.6%
20%	10.0%	17.9%		20%	6.0%	15.2%

Table 1 shows efficacy in an intention to screen analysis and the corresponding power for different levels of compliance by study arm. The power is calculated based on the study design to compare each arm separately to the control group (N=120 372).

Table 2, randomization

	**Birthyear**	**Date of randomization**	**Arms**
**Initial randomization**	1954<br>1955<br>1956	2014-02-11<br>2015-03-31<br>2016-03-01	All
**Extra randomization**	1957<br>1957<br>1958	2017-05-30*<br>2017-06-29**<br>2018-05-25	Primary colonoscopy and control

Table 2 shows the dates of randomization per year and which arms individuals were randomized to.

* All regions except Gävleborg ** Gävleborg only

Table 3, power and last date of follow-up

Comparison	Date	Primary analysis: Deaths from CRC	Primary analysis: Power (%)	Sensitivity analysis 1: Deaths from CRC	Sensitivity analysis 1: Power (%)	Sensitivity analysis 2: Deaths from CRC	Sensitivity analysis 2: Power (%)
**Direct colonoscopy vs control**	2024-12-31	692	28.0	900	35.7	-	-
	2025-05-24	735	29.6	956	37.7	-	-
	2025-12-31	804	32.2	1044	41.6	-	-
	2026-05-24	850	34.3	1104	44.1	-	-
	2026-12-31	923	36.7	1199	47.9	-	-
	2027-05-24	973	38.2	1264	50.5	-	-
	2027-12-31	1051	41.6	1365	53.9	-	-
	2028-05-24	1105	43.6	1435	56.1	-	-
	2028-12-31	1188	46.3	1543	59.4	-	-
	2029-05-24	1234	48.1	1603	62.2	1244	49.1
	2029-12-31	1302	51.5	1690	65.0	1332	52.8
	2030-05-24	1342	52.8	1742	66.3	1391	54.4
	**2030-12-31**	1392	54.9	1807	67.8	1482	57.6
	2031-05-24	1420	55.8	1844	68.6	1543	60.1
	2031-12-31	1452	57.4	1885	69.7	1638	63.3
	2032-05-24	1473	57.7	1913	70.6	1701	64.9
	2032-12-31	1490	58.4	1935	70.8	1798	67.6
	2033-05-24	1501	58.7	1949	71.3	1861	70.1
**FIT vs control**	2024-12-31	641	35.4	832	46.5	-	-
	2025-05-24	678	37.3	881	49.4	-	-
	2025-12-31	737	40.7	958	53.4	-	-
	2026-05-24	777	42.9	1010	55.6	-	-
	2026-12-31	840	47.0	1092	59.8	-	-
	2027-05-24	883	50.2	1147	61.8	-	-
	2027-12-31	951	53.0	1235	65.5	-	-
	2028-05-24	997	54.6	1294	67.7	-	-
	2028-12-31	1068	58.2	1387	71.5	-	-
	2029-05-24	1104	59.8	1433	73.4	1116	60.3
	2029-12-31	1152	61.9	1496	74.9	1190	63.5
	2030-05-24	1179	63.7	1530	76.6	1240	65.8
	**2030-12-31**	1204	64.4	1563	77.5	1317	68.4
	2031-05-24	1214	64.8	1576	77.8	1368	70.3
	2031-12-31	1214	64.8	1576	77.8	1447	72.6
	2032-05-24	1214	64.8	1576	77.8	1500	74.6
	2032-12-31	1214	64.8	1576	77.8	1579	77.6
	2033-05-24	1214	64.8	1576	77.8	1631	79.1

Table 3 shows the power and number of deaths from colorectal cancer (CRC) as a function of the last date of follow-up in the primary analysis and two sensitivity analyses. Results were based on 5000 simulations and the Monte-Carlo Standard Error was between 0.57-0.71%. In the first sensitivity analysis the risk of death from CRC was higher in all arms (HR=1.3 compared to primary analysis), and in the second sensitivity analysis the follow-up extended until the date of end of follow-up or death from other causes (i.e. potentially extending beyond 15 years from date of randomization).

References

1. Socialstyrelsen. Dödsorsaker [internet]: Stockholm: Socialstyrelsen; 2024 [2024-10-09]. Available from: https://www.socialstyrelsen.se/statistik-och-data/statistik/statistikdatabasen/.
2. Mandel JS, Bond JH, Church TR, Snover DC, Bradley GM, Schuman LM, et al. Reducing mortality from colorectal cancer by screening for fecal occult blood. New England Journal of Medicine. 1993;328(19):1365-71.
3. Kronborg O, Fenger C, Olsen J, Jørgensen OD, Søndergaard O. Randomised study of screening for colorectal cancer with faecal-occult-blood test. The Lancet. 1996;348(9040):1467-71.
4. Hardcastle JD, Chamberlain JO, Robinson MH, Moss SM, Amar SS, Balfour TW, et al. Randomised controlled trial of faecal-occult-blood screening for colorectal cancer. The Lancet. 1996;348(9040):1472-7.
5. Lindholm E, Brevinge H, Haglind E. Survival benefit in a randomized clinical trial of faecal occult blood screening for colorectal cancer. Journal of British Surgery. 2008;95(8):1029-36.
6. Atkin WS, Edwards R, Kralj-Hans I, Wooldrage K, Hart AR, Northover JM, et al. Once-only flexible sigmoidoscopy screening in prevention of colorectal cancer: a multicentre randomised controlled trial. The Lancet. 2010;375(9726):1624-33.
7. Segnan N, Armaroli P, Bonelli L, Risio M, Sciallero S, Zappa M, et al. Once-only sigmoidoscopy in colorectal cancer screening: follow-up findings of the Italian Randomized Controlled Trial—SCORE. Journal of the National Cancer Institute. 2011;103(17):1310-22.
8. Schoen RE, Pinsky PF, Weissfeld JL, Yokochi LA, Church T, Laiyemo AO, et al. Colorectal-cancer incidence and mortality with screening flexible sigmoidoscopy. New England Journal of Medicine. 2012;366(25):2345-57.
9. Forsberg A, Westerberg M, Metcalfe C, Steele R, Blom J, Engstrand L, et al. Once-only colonoscopy or two rounds of faecal immunochemical testing 2 years apart for colorectal cancer screening (SCREESCO): preliminary report of a randomised controlled trial. Lancet Gastroenterol Hepatol. 2022;7(6):513-21.
10. Stromberg U, Bonander C, Westerberg M, Levin LA, Metcalfe C, Steele R, et al. Colorectal cancer screening with fecal immunochemical testing or primary colonoscopy: An analysis of health equity based on a randomised trial. EClinicalMedicine. 2022;47:101398.
11. Gray RJ. A class of K-sample tests for comparing the cumulative incidence of a competing risk. The Annals of statistics. 1988:1141-54.
12. Zhang MJ, Fine J. Summarizing differences in cumulative incidence functions. Statistics in Medicine. 2008;27(24):4939-49.
13. Statistics Sweden Statistikdatabasen 2024 [2024-10-09]. Available from: www.statistikdatabasen.scb.se.
14. Fritzell K, Forsberg A, Wangmar J, Wengstrom Y, Bottai M, Hultcrantz R. Gender, having a positive FIT and type of hospital are important factors for colonoscopy experience in colorectal cancer screening - findings from the SCREESCO study. Scand J Gastroenterol. 2020;55(11):1354-62.
15. Aronsson M, Carlsson P, Levin LA, Hager J, Hultcrantz R. Cost-effectiveness of high-sensitivity faecal immunochemical test and colonoscopy screening for colorectal cancer. Br J Surg. 2017;104(8):1078-86.
16. Ribbing Wilen H, Blom J, Hoijer J, Andersson G, Lowbeer C, Hultcrantz R. Fecal immunochemical test in cancer screening - colonoscopy outcome in FIT positives and negatives. Scand J Gastroenterol. 2019;54(3):303-10.
