# Supplementary material for: Colonoscopy and fecal immunochemical testing versus usual care in diagnostic colorectal cancer screening: the SCREESCO randomized controlled trial
Source: Nat Med. 2026 Feb 20;32(4):1278–85. doi: 10.1038/s41591-026-04225-9 (PMC13099434; doi:10.1038/s41591-026-04225-9)
Supplement: Supplementary file 2 — Reporting Summary [file 41591_2026_4225_MOESM2_ESM.pdf]

nature portfolio

Corresponding author(s): Marcus Westerberg

Last updated by author(s): 12/15/2025

Reporting Summary

Nature Portfolio wishes to improve the reproducibility of the work that we publish. This form provides structure for consistency and transparency in reporting. For further information on Nature Portfolio policies, see our Editorial Policies and the Editorial Policy Checklist.

Statistics

For all statistical analyses, confirm that the following items are present in the figure legend, table legend, main text, or Methods section.

n/a	Confirmed	
☐	☒	The exact sample size (*n*) for each experimental group/condition, given as a discrete number and unit of measurement
☒	☐	A statement on whether measurements were taken from distinct samples or whether the same sample was measured repeatedly
☒	☐	The statistical test(s) used AND whether they are one- or two-sided<br>*Only common tests should be described solely by name; describe more complex techniques in the Methods section.*
☐	☒	A description of all covariates tested
☐	☐	A description of any assumptions or corrections, such as tests of normality and adjustment for multiple comparisons
☐	☒	A full description of the statistical parameters including central tendency (e.g. means) or other basic estimates (e.g. regression coefficient) AND variation (e.g. standard deviation) or associated estimates of uncertainty (e.g. confidence intervals)
☒	☐	For null hypothesis testing, the test statistic (e.g. *F*, *t*, *r*) with confidence intervals, effect sizes, degrees of freedom and *P* value noted<br>*Give P values as exact values whenever suitable.*
☒	☐	For Bayesian analysis, information on the choice of priors and Markov chain Monte Carlo settings
☒	☐	For hierarchical and complex designs, identification of the appropriate level for tests and full reporting of outcomes
☐	☒	Estimates of effect sizes (e.g. Cohen's *d*, Pearson's *r*), indicating how they were calculated

Our web collection on statistics for biologists contains articles on many of the points above.

Software and code

Policy information about availability of computer code

Data collection	Stata (version 13.1) and R version 4.0.2 were used for power calculations.
Data analysis	Analyses were performed using R version 4.0.2. Code is available at https://github.com/MarcusWesterberg/CRCs-and-AEs-during-diagnostic-phase-of-SCREESCO

For manuscripts utilizing custom algorithms or software that are central to the research but not yet described in published literature, software must be made available to editors and reviewers. We strongly encourage code deposition in a community repository (e.g. GitHub). See the Nature Portfolio guidelines for submitting code & software for further information.

Data

Policy information about availability of data

All manuscripts must include a data availability statement. This statement should provide the following information, where applicable:

- Accession codes, unique identifiers, or web links for publicly available datasets
- A description of any restrictions on data availability
- For clinical datasets or third party data, please ensure that the statement adheres to our policy

The data cannot be shared publicly because the individual-level data contain potentially identifying and sensitive patient information and cannot be published due to legislation and ethical approval (https://etikprovningsmyndigheten.se). Use of the data from national health-data registers is further restricted by the Swedish Board of Health and Welfare (https://www.socialstyrelsen.se/en/) and Statistics Sweden (https://www.scb.se/en/) which are Government Agencies providing access

to the linked healthcare registers. Selected deidentified individual participant data that underlie the results reported in this Article (including in the supplement), can however be made available to researchers after request to the SCREESCO Steering Committee. Researchers must provide a methodologically sound proposal for a project that conforms with the Swedish Ethical Review Authority permit for the project and will need to sign a data access agreement. Data will be made available at a secure remote server to achieve the aims in the approved proposal. Data will be available from 3 months after publication and until 3 years after publication of the Article. Proposals regarding the data underlying this Article may be submitted up to 2 years after publication. The SCREESCO study will not carry the costs of external projects. The full trial protocol and statistical analysis plan (including original and revised versions) are available in Supplementary Information and the most recent version of each document is available at https://clinicaltrials.gov/study/NCT02078804.

Research involving human participants, their data, or biological material

Policy information about studies with human participants or human data. See also policy information about sex, gender (identity/presentation), and sexual orientation and race, ethnicity and racism.

Reporting on sex and gender	Sex-based analyses are included
Reporting on race, ethnicity, or other socially relevant groupings	Country of birth (Sweden and other) is reported in Table 1
Population characteristics	Sex, year of randomization, health care region, educational level, comorbidity
Recruitment	We did a randomized controlled trial with a study base population from 18 out of 21 regions in Sweden comprising 74.5% of the total national population where CRC screening had not previously been offered (Stockholm, Gotland, and Västernorrland were not included). Residents aged 60 years in the year of randomization (2014-2018) were identified through the Swedish Total Population Register. Individuals who had a previous diagnosis of CRC or anal cancer or who had participated in the ongoing NordICC trial were excluded.
Ethics oversight	The Stockholm Ethics Committee approved the study (2012/2058-31/3) and the review of medical charts (2015/1958-2). The Swedish Ethical Review Authority waived the need for informed consent for accessing pseudonymised register-based data (2022/01946-02 and 2022/06863-2).

Note that full information on the approval of the study protocol must also be provided in the manuscript.

Field-specific reporting

Please select the one below that is the best fit for your research. If you are not sure, read the appropriate sections before making your selection.

☒ Life sciences ☐ Behavioural & social sciences ☐ Ecological, evolutionary & environmental sciences

For a reference copy of the document with all sections, see nature.com/documents/nr-reporting-summary-flat.pdf

Life sciences study design

All studies must disclose on these points even when the disclosure is negative.

Sample size	A randomized block method was used to assign individuals born 1954-1956 without prior CRC diagnosis to once-only primary colonoscopy, two rounds of fecal immunochemical testing 2 years apart (FITx2), or a usual care control arm with no organised program of screening activity (controls). Masking was not possible due to the nature of the trial.<br><br>The sample size was calculated using the STATA function "stpower" on the basis of the primary endpoint of colorectal cancer mortality. The original sample size target was 201 000 individuals, based on an assumed 1% cumulative colorectal cancer mortality for a follow-up from age 60 to 75 years. Because of lower participation (35%) in the primary colonoscopy arm than expected (50%), two additional age cohorts (born 1957 and 1958) were randomized to primary colonoscopy or control. New power calculations were performed based on the lower participation rate in the primary colonoscopy arm to determine the additional number of randomized individuals.<br><br>In the revised study plan, we assumed a 15% disease-specific mortality reduction by 15 years of follow-up as a minimal clinically important effect in those invited to FITx2, based on a participation rate of 50%, and a 17.5% disease-specific mortality reduction in those invited to colonoscopy, based on a 35% participation rate. To allow differences to be detected at a two-sided 2.5% significance level using the log rank test with 80% power for the comparison of FITx2 versus control and 73% power for the comparison of primary colonoscopy versus control the revised target sample size was 278 280 participants. The significance level was adjusted for two comparisons according to the Bonferroni method. In total, 31 140 individuals were randomized to the primary colonoscopy arm, 60 300 to FITx2, and two control groups: 186 840 controls to the primary colonoscopy arm out of which 120 600 individuals also were controls to the FITx2 arm (FITx2 controls).
Data exclusions	Between February 11, 2014, and May 25, 2018, 278 280 individuals were randomized. Due to administrative lag in registration, 159 dead and 70 with prevalent CRC before the date of randomization were identified only after randomization and were excluded. Another two control individuals were excluded since they were not identifiable in any register.
Replication	RCT of individuals randomized to primary colonposcopy, FIT or usual care, so not replicable
Randomization	A randomized block method was used to assign individuals without prior CRC diagnosis to once-only primary colonoscopy, two rounds of fecal immunochemical testing 2 years apart (FITx2), or a usual care control arm with no organised program of screening activity (controls).

	We have not identified any reasons for self-selection bias or other biases in the randomization process.
Blinding	Masking/blinding was not possible due to the nature of the trial.

Reporting for specific materials, systems and methods

We require information from authors about some types of materials, experimental systems and methods used in many studies. Here, indicate whether each material, system or method listed is relevant to your study. If you are not sure if a list item applies to your research, read the appropriate section before selecting a response.

Materials & experimental systems

n/a	Involved in the study	
☒	☐	Antibodies
☒	☐	Eukaryotic cell lines
☒	☐	Palaeontology and archaeology
☒	☐	Animals and other organisms
☐	☒	Clinical data
☒	☐	Dual use research of concern
☒	☐	Plants

Methods

n/a	Involved in the study	
☒	☐	ChIP-seq
☒	☐	Flow cytometry
☒	☐	MRI-based neuroimaging

Clinical data

Policy information about clinical studies
All manuscripts should comply with the ICMJE guidelines for publication of clinical research and a completed CONSORT checklist must be included with all submissions.

Clinical trial registration	ClinicalTrials.gov NCT02078804
Study protocol	https://clinicaltrials.gov/study/NCT02078804 and in Supplementary Information
Data collection	We did a randomized controlled trial with a study base population from 18 out of 21 regions in Sweden comprising 74.5% of the total national population where CRC screening had not previously been offered (Stockholm, Gotland, and Västernorrland were not included). Residents aged 60 years in the year of randomization (2014-2018) were identified through the Swedish Total Population Register and randomly allocated.
Outcomes	The primary outcome of the trial is colorectal cancer mortality and secondary outcomes include incidence of colorectal cancer and adverse events, see the most recent version of the Study Protocol. In the current analysis we analyzed these secondary outcomes during the diagnostic phase of the trial, i.e. diagnostic yield of screening and usual care, in terms of CRC diagnoses (overall and by stage), and adverse events (cardiovascular and gastrointestinal and death from any cause). These were ascertained using national data registries and assessed using incidence rates and incidence rate ratios for comparison between intervention and control.

Plants

Seed stocks	*Report on the source of all seed stocks or other plant material used. If applicable, state the seed stock centre and catalogue number. If plant specimens were collected from the field, describe the collection location, date and sampling procedures.*
Novel plant genotypes	*Describe the methods by which all novel plant genotypes were produced. This includes those generated by transgenic approaches, gene editing, chemical/radiation-based mutagenesis and hybridization. For transgenic lines, describe the transformation method, the number of independent lines analyzed and the generation upon which experiments were performed. For gene-edited lines, describe the editor used, the endogenous sequence targeted for editing, the targeting guide RNA sequence (if applicable) and how the editor was applied.*
Authentication	*Describe any authentication procedures for each seed stock used or novel genotype generated. Describe any experiments used to assess the effect of a mutation and, where applicable, how potential secondary effects (e.g. second site T-DNA insertions, mosiacism, off-target gene editing) were examined.*
